# Design of a delivery vehicle chitosan-based self-assembling: controlled release, high hydrophobicity, and safe treatment of plant fungal diseases

**DOI:** 10.1186/s12951-024-02386-8

**Published:** 2024-03-19

**Authors:** Qing Zhou, Zhi Xia, Yu Zhang, Zhiling Sun, Wei Zeng, Nian Zhang, Chunmei Yuan, Chenyu Gong, Yuanxiang Zhou, Wei Xue

**Affiliations:** 1 National Key Laboratory of Green Pesticide, Key Laboratory of Green Pesticide and Agricultural Bioengineering, Ministry of Education, Center for R&D of Fine Chemicals of Guizhou University, Guiyang, 550025 China; 2 College of Chemistry and Chemical Engineering, Guizhou University of Engineering Science, Bijie, 551700 China

**Keywords:** *N*-succinyl chitosan, Nanopesticide, Bioactive molecule, Anti-fungal activity

## Abstract

**Background:**

Traditional pesticides are poorly water-soluble and suffer from low bioavailability. *N*-succinyl chitosan (NSCS) is a water-soluble chitosan derivative, has been recently used to encapsulate hydrophobic drugs to improve their bioavailability. However, it remains challenging to synthesize pesticides of a wide variety of water-soluble drugs and to scale up the production in a continuous manner.

**Results:**

A synthetic method for preparing water-soluble nanopesticides with a polymer carrier was applied. The bioactive molecule BTL-11 was loaded into hollow NSCS to promote drug delivery, improve solubility and anti-fungal activity. The synthesized nanopesticides had well controlled sizes of 606 nm and the encapsulation rate was 80%. The release kinetics, drug toxicity and drug activity were further evaluated. The inhibitory activity of nanopesticides against *Rhizoctonia solani* (*R. solani*) was tested in vivo and in vitro. In vivo against *R. solani* trials revealed that BTL-11 has excellent control efficiency for cultivated rice leaf and sheath was 79.6 and 76.5%, respectively. By contrast, for BTL-11@NSCS NPs, the anti-fungal ability was strongly released and afforded significant control efficiencies of 85.9 and 81.1%. Those effects were significantly better than that of the agricultural fungicide azoxystrobin (51.5 and 66.5%). The proposed mechanism was validated by successfully predicting the synthesis outcomes.

**Conclusions:**

This study demonstrates that NSCS is a promising biocompatible carrier, which can enhance the efficacy of pesticides, synergistically improve plant disease resistance, protect crop growth, and can be used for the delivery of more insoluble pesticides.

**Graphical Abstract:**

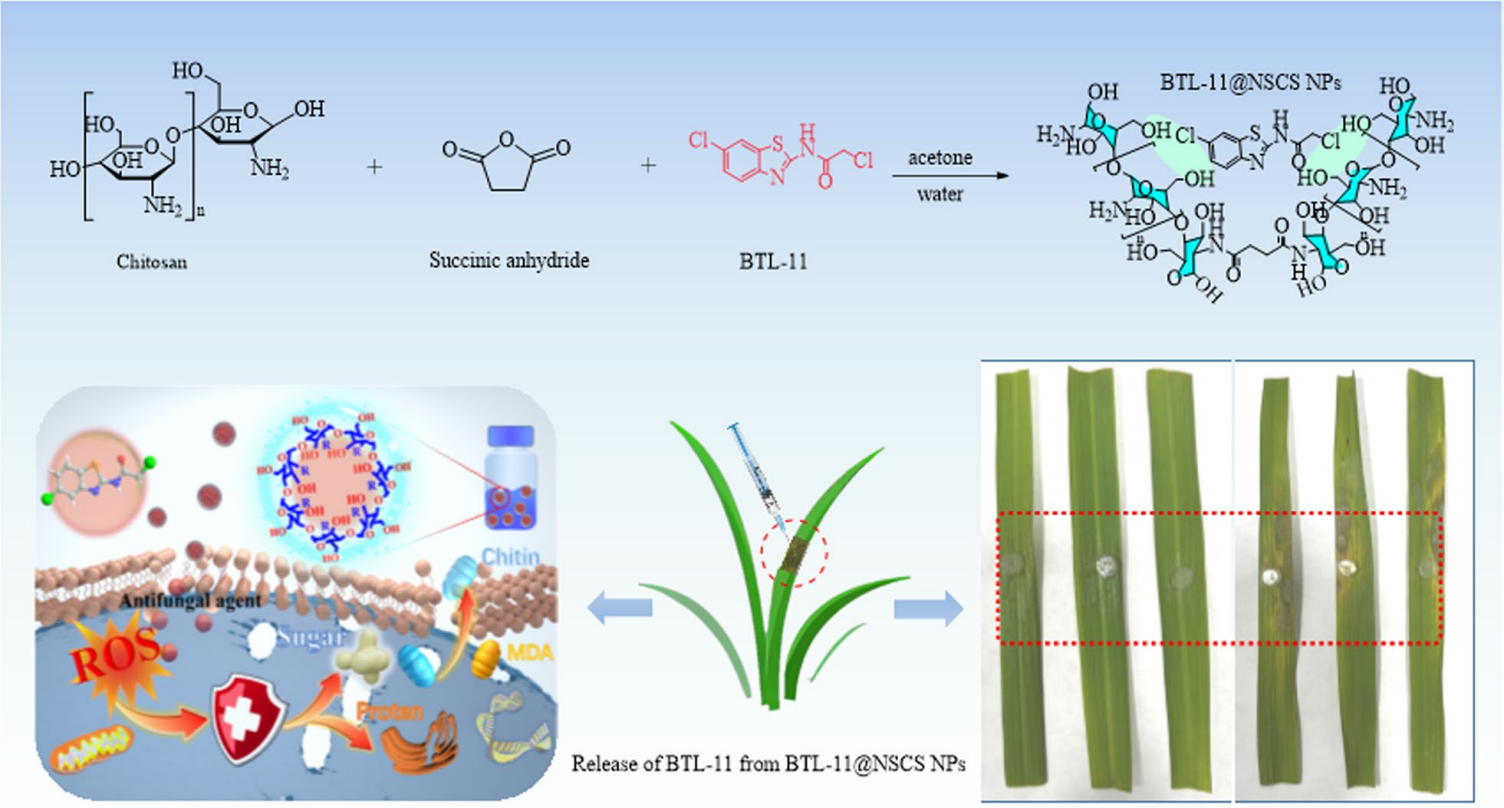

**Supplementary Information:**

The online version contains supplementary material available at 10.1186/s12951-024-02386-8.

## Highlights

- *N*-succinyl chitosan as a delivery vehicle material.
- Green, efficient, and safe BTL-11@NSCS NPs nanopesticides were prepared by utilizing NSCS in combination with the bioactive molecule BTL-11.
- BTL-11@NSCS NPs has a small, uniform, stable morphology feature, and outstanding physicochemical properties.
- Controlled release, high hydrophobicity, and safe treatment of plant fungal diseases.

**Supplementary Information:**

The online version contains supplementary material available at 10.1186/s12951-024-02386-8.

## Introduction

Rice is considered one of the most important plants globally, as it is the source of food for more than half of the world’s population [1–3]. However, fungal diseases are increasingly recognized as a global threat to food security, crop destruction and forest ecosystem dynamics [4–6]. Rice plants are susceptible to rice blast, leaf blight, and stripe blight, and the occurrence of multiple rice diseases can negatively affect crop growth and lead to substantial yield loss in all rice growing regions of the world [7–9]. Since 1960s, a multitude of cost effective fungicides have been used to protect crops from fungal infections. Those played an instrumental role in dealing with the demand for food as a result of rapid population growth [10–14]. Nevertheless, owing to the excessive use of fungicides with the same or similar mode of action, fungicide resistance is rapidly increasing, which resulting in the decrease of ability to control fungal diseases in crops [15–17]. Meanwhile, non-target and environmental hazards have emerged along with fungicide utilization [18, 19]. Therefore, it is an outstandingly meaningful project to continue to develop green, efficient, and environmentally friendly new fungicides to control fungal diseases in crops.

In recent decades, nanotechnology has been developing rapidly and widely used in many fields, and it is like a rising star for bio-agriculture, offering many strategies to address the drawbacks of traditional pesticides, agricultural design and manufacture of green pesticide formulations [20–22]. Nanopesticides is a sign of the technological development of pesticides, which has efficacy, durability, and reduces the amount of active ingredient required [23–25]. The development and characterization of green composites are based on natural fibers, especially chitosan, chitosan blends, and chitosan nanocomposites, which have attracted much attention due to their applications in the fields of bio-medicine, bio-industry, drug slow-release materials, and environmental protection [26–28]. Chitosan (CS) is an amino polysaccharide obtained from the partial or total deacetylation of natural polymer chitin. CS has rich amine, hydroxyl and other active functional groups [29–31], which attracted significant attention due to its bio-degradability, bio-compatibility and bio-activity, and has been applied to adsorbent tablets, nanoparticles, films, hydrogels, and so on [32–35]. Although chitosan itself is insoluble in water, a variety of chitosan derivatives with different properties can generate through chemical reactions under the premise of chemical modification of chitosan, thus expanding the scope of application of chitosan [36]. Among them, the inhibitory effect of chitosan on plant fungal diseases plays a bridging role in agricultural engineering [37–39]. However, there are few studies on the use of CS as a carrier to deliver active ingredients and make value of waste material. Considering the unique properties of CS, it is hypothesized that it could interact with NSCS polymers through non-covalent molecular recognition to form an assembled system, thereby building an efficient [40–42], multifunctional and sustainable pesticide delivery platform.

**Fig. 1:**
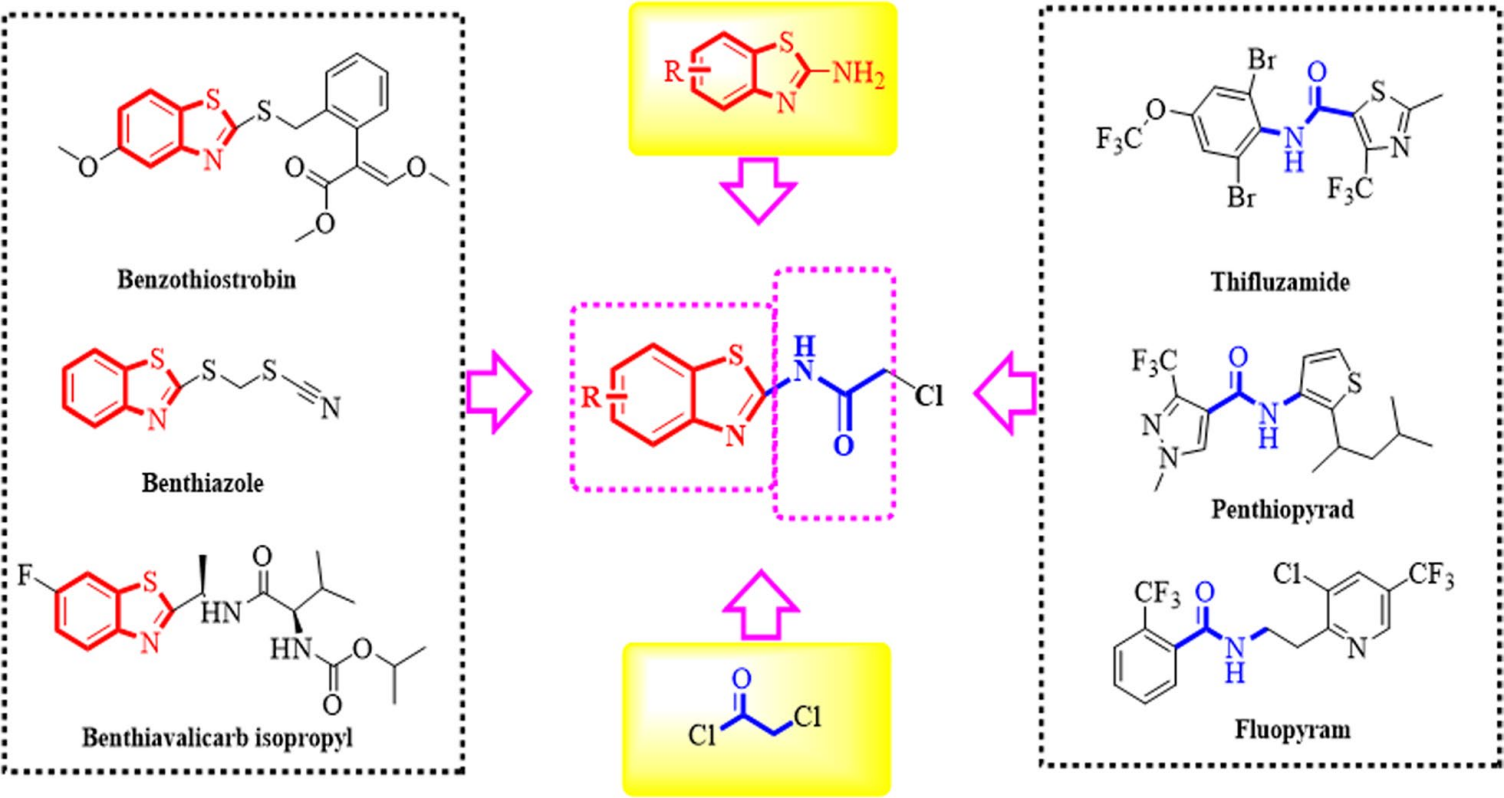
Design of target compounds by using active structure splicing

The succinate dehydrogenase inhibitors (SDHI) fungicides with amide bridge has been successfully developed and commercially utilized, which exhibited significant fungicidal efficacies and low cross-resistance [43]. By using active structure splicing (Fig. 1), 24 benzothiazole amides active small molecules were synthesized. The BTL-11 possessed a wide range of anti-fungal activities, especially significant in vitro inhibitory activity against rice blast fungus. However, since the BTL-11 is insoluble in water, it is difficult to be applied in production practice. Further, the bioactive molecule BTL-11 was encapsulated in an aqueous solution of NSCS to obtain novel BTL-11@NSCS NPs pesticides. Fourier transform infrared spectroscopy (FTIR), transmission electron microscopy (TEM), dynamic light scattering (DLS), and fluorescence spectroscopy (FS) were used to characterize the physicochemical properties of NSCS and BTL-11@NSCS NPs. Moreover, the BTL-1–BTL-24 and BTL-11@NSCS NPs complex would be evaluated with plant disease as well as in vitro and in vivo anti-fungal activities against phytopathogen *Rhizoctonia solani* (*R. solani*). Finally, the anti-fungal mechanism would be investigated from pathogen’ morphological studies, fluorescent staining results, and enzyme activity test experiments. The results displayed that BTL-11@NSCS NPs could interfere with the synthesis of the cell wall of *R. solani*, destroy the cell membrane, cause the separation of the cell wall, enhance the permeability of the cell, enter the cell and act on a variety of organelles, eventually leading to serious damage to the cell structure, causing the mycelium to wilt and fold, unable to grow normally or even die.

## Methods

### Instruments and chemicals

The NMR data ^1^H, ^13^C, and ^19^F of BTL-1–BTL-24 were determined on Bruker Biospin AG-400 NMR spectrometer (Bruker Optics, Switzerland). *X*-ray single crystal structure data were obtained on *X*-ray diffractometer (Bruker, Germany). Scanning electron microscopy (SEM) data were obtained on FEI Nova Nano 450 (Hillsboro, OR, USA). FTIR date were obtained on Thermo Fisher Scientific (USA). TEM and Energy dispersive *X*-ray spectroscopy (EDS) data were obtained on FEI Talos F200X (USA). The critical micelle concentration (CMC) was determined by F98 fluorescence spectrophotometer (Lengguang Technology, China). Particle size distribution and zeta potential (in liquid) were determined by Zetasizer Nano ZS90 (Malvern, UK).

The chitosan (deacetylation degree > 95% and viscosity of 100–200 mPa s), succinic anhydride, acetone, pyrene, ethyl alcohol, thiourea, substituted 2-chlorophenol, 2-amino-substituted benzothiazole, and ethyl acetate were purchased from Tansoole Chemicals Company (Adamas, Shanghai, China). Various assay kits were purchased from Beijing Solarbio Science & Technology Co., Ltd.

### The preparation method of the target compounds BTL-1–BTL-24

A variety of substituted amines reacted with chloroacetyl chloride (at molar ratio of 1:1.2) in CH_2_Cl_2_ system in ice bath for 1 h to obtain corresponding benzothiazole amides [44].

### Preparation of NSCS and BTL-11@NSCS NPs

**Scheme 1:**
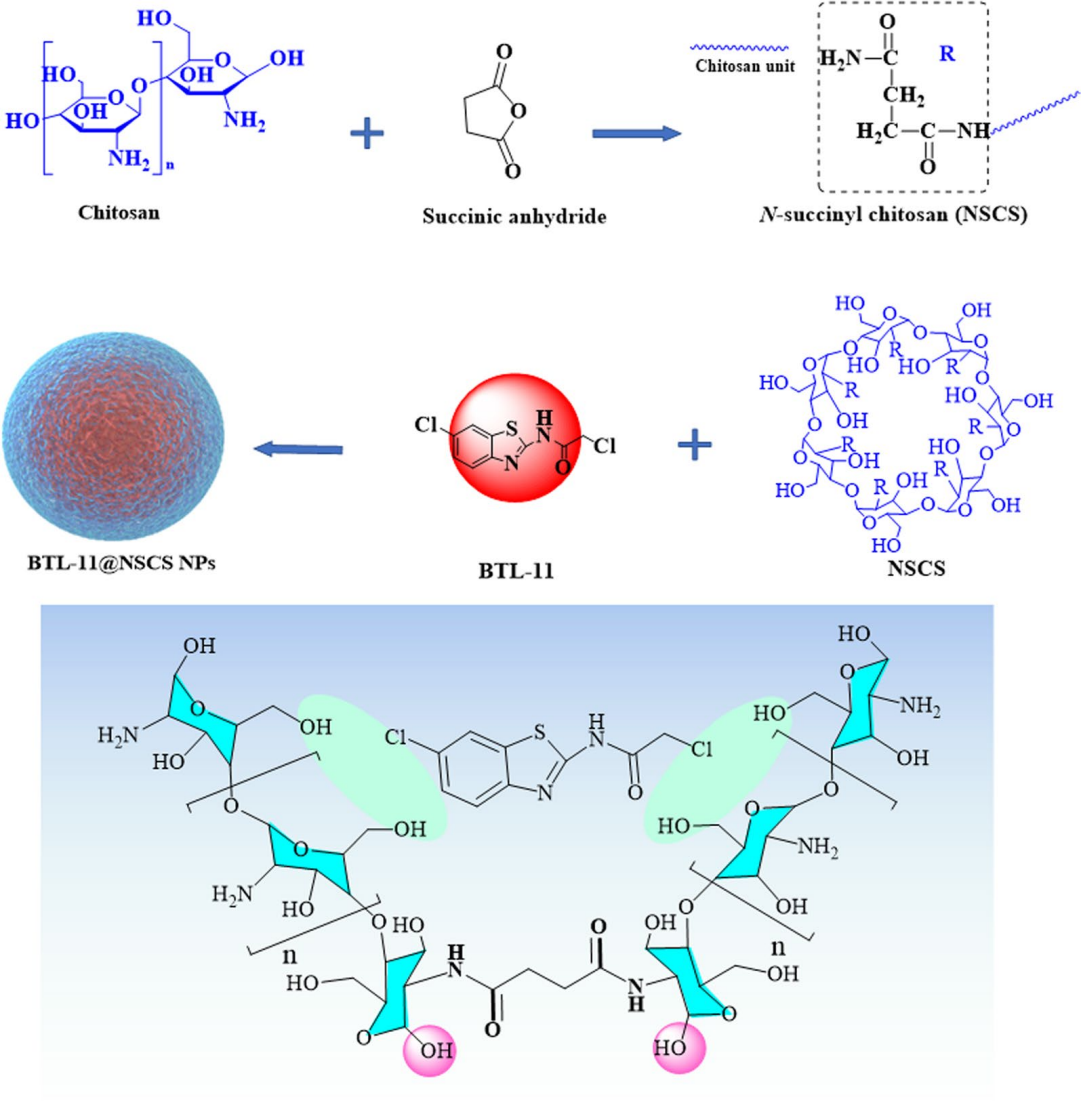
Schematic diagram of the synthesis principle of BTL-11@NSCS NPs based CS

CS (1.0 g) was dissolved in 200 mL of 1% acetic acid solution (in a three-neck flask) under stirring. Dissolve 0.2 g succinic anhydride in 20 mL acetone, slowly add it dropwise to the above chitosan acetic acid solution at room temperature, and then stir at 40 °C for 4 h. After cooling, add excessive acetone for precipitation. Remove solvent by vacuum suction filtration. Finally, the product was dried in vacuum at 40 °C, and the faint yellow substance obtained was NSCS. NSCS was ultrasonically dissolved in distilled water and prepared into aqueous solution (1.0 mg/mL). Next, different amounts of BTL-11 were added to the above solution to form an emulsion by ultrasonic dispersion to obtain BTL-11@NSCS nanoparticles (BTL-11@NSCS NPs) with different drug loading [45]. The synthesis principle was shown in Scheme 1.

The critical micelle concentration (CMC).

NSCS micellar water dispersions with a certain concentration were prepared. The diluted concentrations were 1.0, 0.8, 0.6, 0.4, 0.2, 10^–1^, 10^–2^, 10^–3^, 10^–4^, 10^–5^ mg/mL, and 10 μL of 0.6 μmol/L pyrene acetone solution was added respectively and it was left in a vacuum drying oven overnight at 50 °C. The fluorescence detection was carried out after pyrene molecules were stable in the system. Detection conditions of fluorescence spectrophotometer: scanning wavelength range: 350–450 nm, excitation wavelength: 337 nm, scanning speed: 60 nm/min, excitation and emission slit width: 5.0 nm [46].

### Loading content (LC) and encapsulation efficiency (EE)

BTL-11 (1.0, 2.0, and 5.0 mg) was ultrasonically dissolved in 10 mL of NSCS aqueous solution (1.0 mg/mL), respectively. Then the solution was centrifuged at high speed for 3 min, and the supernatant was taken and filtered through a filer membrane with 0.22 μm pore size. The amount of BTL-11 entrapped or adsorbed in the NSCS NPs was determined by HPLC. The operating conditions for HPLC determination were as follows: Agilent XDB-C18 reverse phase column (5 mm, 4.6 × 250 mm), column temperature (30 °C), acetonitrile and aqueous solution (*V/V* = 60:40) were used as mobile phase with a flow rate of 1.0 mL/min. The detection wavelength was set at 310 nm and the injection volume was 5 μL.

### Drug release and phytotoxicity tests

The influence of pH on NPs and especially on nanocapsules is significant factor [47, 48]. The mixture of NSCS and BTL-11 (NSCS:BTL-11 = 2:1) was ultrasonically dispersed in 50 mL distilled water. Five different pH values of 5.0, 6.0, 7.0, 8.0 and 9.0 were set to investigate the relationship between pH value and release behavior. These solutions were shaken in a constant temperature shaker (Shanghai Yiheng Scientifc Instrument Co., Ltd., Shanghai, China) with a speed of 200 rpm at 30 °C. The released solution of 3 mL was withdrawn at different time intervals for analysis. To keep the total solution volume as constant, an equal volume of phosphate buffer solution (PBS) with different pH values (3 mL) was further added. The concentration of BTL-11 in the solution was determined by UV spectrometry (209 nm). The accumulative BTL-11 released was calculated according to the following equation [49, 50].

$${\text{Release}}\,\left( \% \right) = \frac{{V_0 \times C_t + V \times \sum_{n - 1}^{t - 1} C }}{W}$$

where Release is the accumulative release of BTL-11 from the hydrogels; *V*_0_ is the volume of the released medium (50 mL); *C*_*t*_ is the concentration (mg/mL) of BTL-11 in the release medium at sampling time; *V* is the volume of each sampling (3 mL); *W* is the total quantity (mg) of BTL-11 entrapped in the NSCS hydrogels.

### In vitro and in vivo of target compounds against *R. solani, P. capsici, B. cinerea*, and *S. sclerotiorum*

The assessed effects of the BTL-1–BTL-24 on the mycelial growth against *Rhizoctonia solani* (*R. solani*), *Sclerotinia sclerotiorum* (*S. sclerotiorum*), *Botrytis cinerea* (*B. cinerea*), and *Phytophthora capsici (P. capsici)* [51, 52].

In vivo protective activity against *R. solani* had been explored. In order to evaluate the protective activity against rice sheath blight, the rice plants were inoculated with *R. solani*, which were treated of target compound solution, and azoxystrobin was used as a positive control [53, 54]. Each was treated with 12 plants, after 72 h the control effect is calculated by the formula:

$${\text{Inhibition ratio}}\,\left( \% \right) = \frac{C - T}{C} \times 100$$

*C* is the diameters of the lesion without treatment; *T* is the diameters of the lesion with treatment.

### Sclerotia formation and germination inhibiting tests

The mycelial disks (5 mm) of *R. solani* were inoculated into potato dextrose agar (PDA) containing (50, 25, 12.5, 6.25, 3.125, and 0 μg/mL) of BTL-11 and BTL-11@NSCS NPs and cultured at 28 °C in the dark for 21 d. The formed sclerotia were collected and dried at 60 °C for 24 h, and the number and weight of the sclerotia were calculated. *R. solani* were cultured for 21 d to obtain sclerotia. Different concentrations (50, 25, 12.5, 6.25, 3.125, and 0 μg/mL) of BTL-11 and BTL-11@NSCS NPs were prepared and then 5 sclerotia were placed on the culture, and each concentration consisted of three replicates. These dishes were incubated at 28 °C for 32 h, and then the inhibition rate of the compounds on the germination of sclerotia was calculated [55].

### Effect on *R. solani* morphology

The references describe detailed procedures for Light microscopy (LM), Fluorescence microscope (FM), and SEM measurements [56].

### Effects on the growth and respiratory energy metabolism, cell wall, and membrane permeability of *R. solani*

Chitinase, malondialdehyde (MDA), protein, and total sugar content were tested by Refs. [57–60].

### Molecular docking

The three-dimensional structures of BTL-11, fluopyram, and oxidoreductase (PDB code: 2FBW) were further treated by Discovery Studio 2019 Client to generate the docking input files and finish molecular docking studies. The obtained three-dimensional binding modes of bioactive molecules with oxidoreductase were shown by Discovery Studio 2019 Client to gain the corresponding two-dimensional binding modes [61].

## Results and discussion

### Characterization of target compounds BTL-1–BTL-24, NSCS, and BTL-11@NSCS NPs

**Scheme 2:**
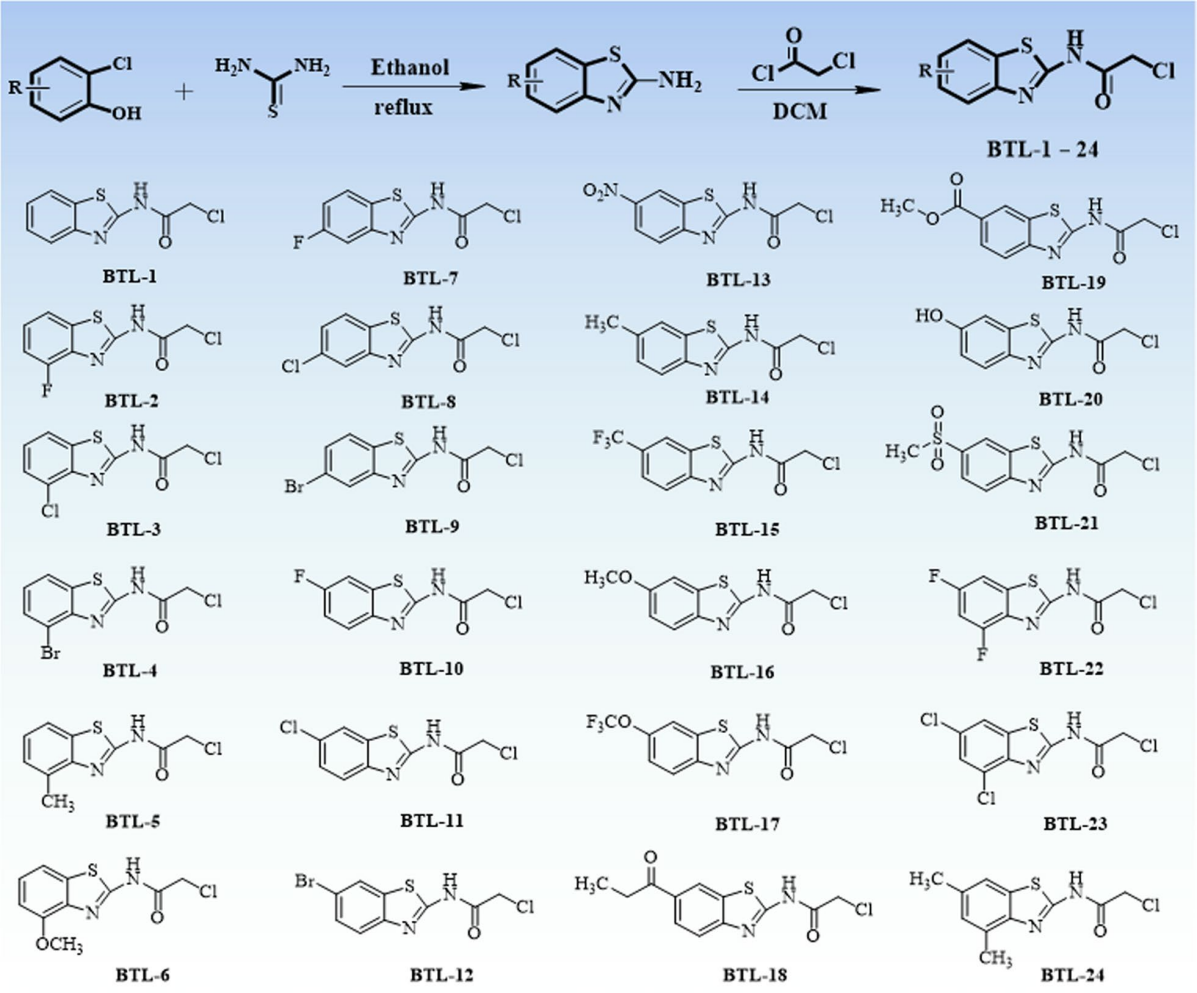
The synthetic rote and structure of target molecules BTL-1–BTL-24

As shown in Scheme 2, 24 benzothiazole amides derivatives were summarized. The NMR and cultured *X*-ray single crystal structure data (BTL-8: Additional file 1: Table S7 and Additional file 1: Fig. S3) of the target compounds were provided in Additional file 1.

**Fig. 2:**
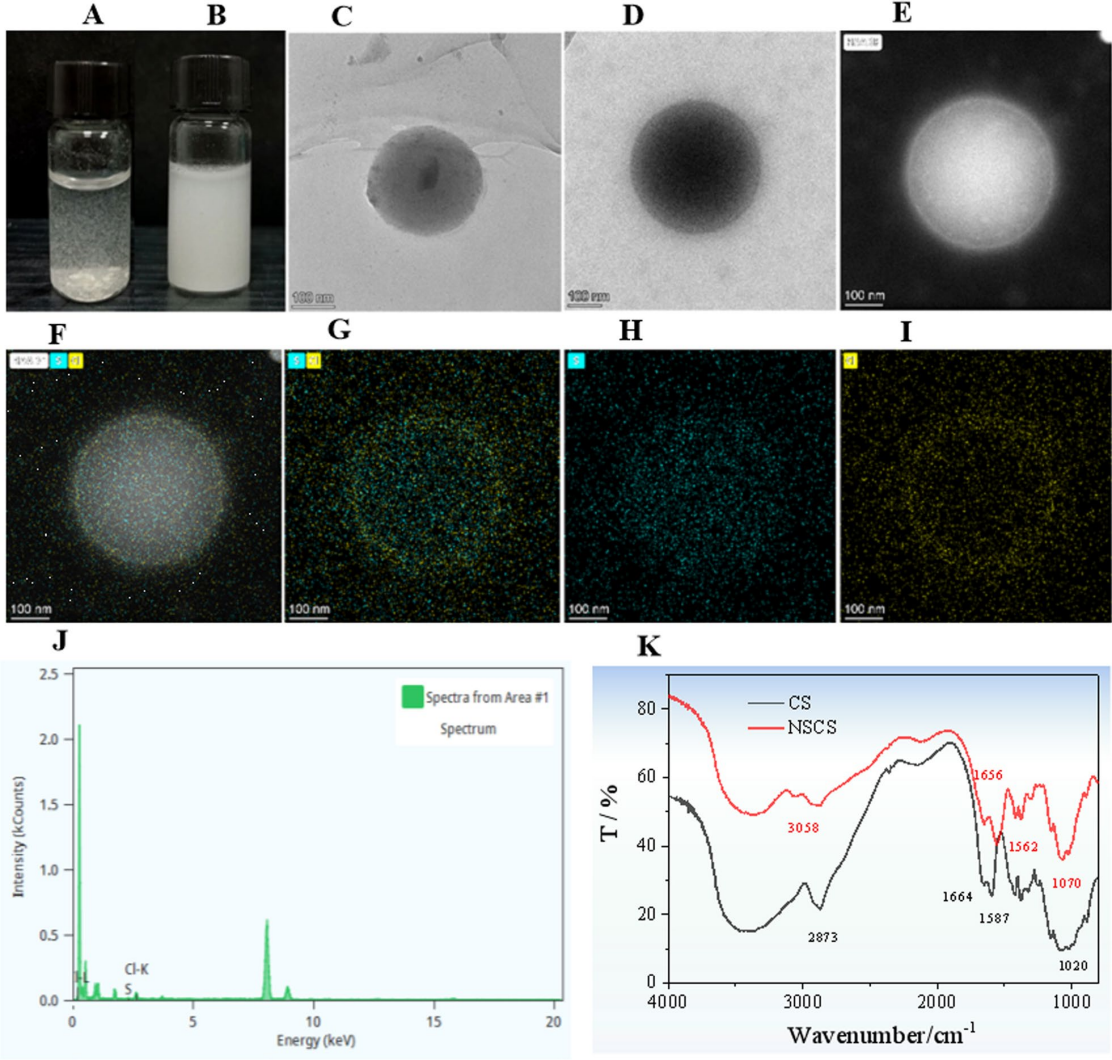
Photographs of solutions of BTL-11 and BTL-11@NSCS NPs 2 mg/mL in water (**A**) and NSCS aqueous solution (**B**); TEM images of NSCS (**C**) and BTL-11@NSCS NPs (**D**, **E**); Elemental mapping (**F–I**) and EDS (**J**) of BTL-11@NSCS NPs; FTIR spectra (**K**) of CS and NSCS

The photographs of solutions of BTL-11 and BTL-11@NSCS NPs 2 mg/mL in water were shown in Fig. 2A. It could be seen from the figure that BTL-11 was in suspension in water, mainly because of its poor water solubility. When it was added to NSCS aqueous solution, it was in white emulsion state, and the dispersion was significantly enhanced (Fig. 2B).

The morphology of the prepared NSCS colloid and BTL-11@NSCS NPs were characterized on TEM. The NSCS colloid exhibited a spherical shape without adsorption or adhesion, and have essentially the same color from the center to the periphery (Fig. 2C). This result indicated that NSCS can self-assemble in distilled water. The spherical color of BTL-11@NSCS NPs was darker, clearer, and more uniform than that of NSCS. This attributed to the fact that BTL-11 contains chlorine, sulfur elements, and has a higher density (Fig. 2D, E). The experiments revealed that BTL-11 could induce NSCS to package and assemble into spherical nanoparticles faster and better. The elemental mapping images of BTL-11@NSCS NPs (Fig. 2F–I) displayed the homogeneous spatial distribution of chlorine and sulphur elements on the BTL-11@NSCS NPs. The EDS measurement further proved the existence of chlorine and sulphur atoms (Fig. 2J).

The FTIR spectrums of NSCS and CS (Fig. 2K) were recorded on KBr pellet method [62]. The absorption bands of –OH and –NH_2_ in the CS at 3310–3500 cm^−1^ were narrowed after the succinylation reaction. 3058 cm^−1^ was the absorption band of –NH, which indicated that the CS was scandalized. 1020 and 1070 cm^−1^ were the absorption bands of the primary hydroxyl and secondary hydroxyl, which changed very little before and after the reaction, respectively. The absorption band at 1857 cm^−1^ disappeared after succinylation, and the amide I band at 1656 cm^−1^ and amide II band at 1562 cm^−1^ appeared in the CS, which further confirmed the formation of –NH–CO– structure in the chitosan molecule.

### Critical micelle concentration (CMC) of NSCS

**Fig. 3:**
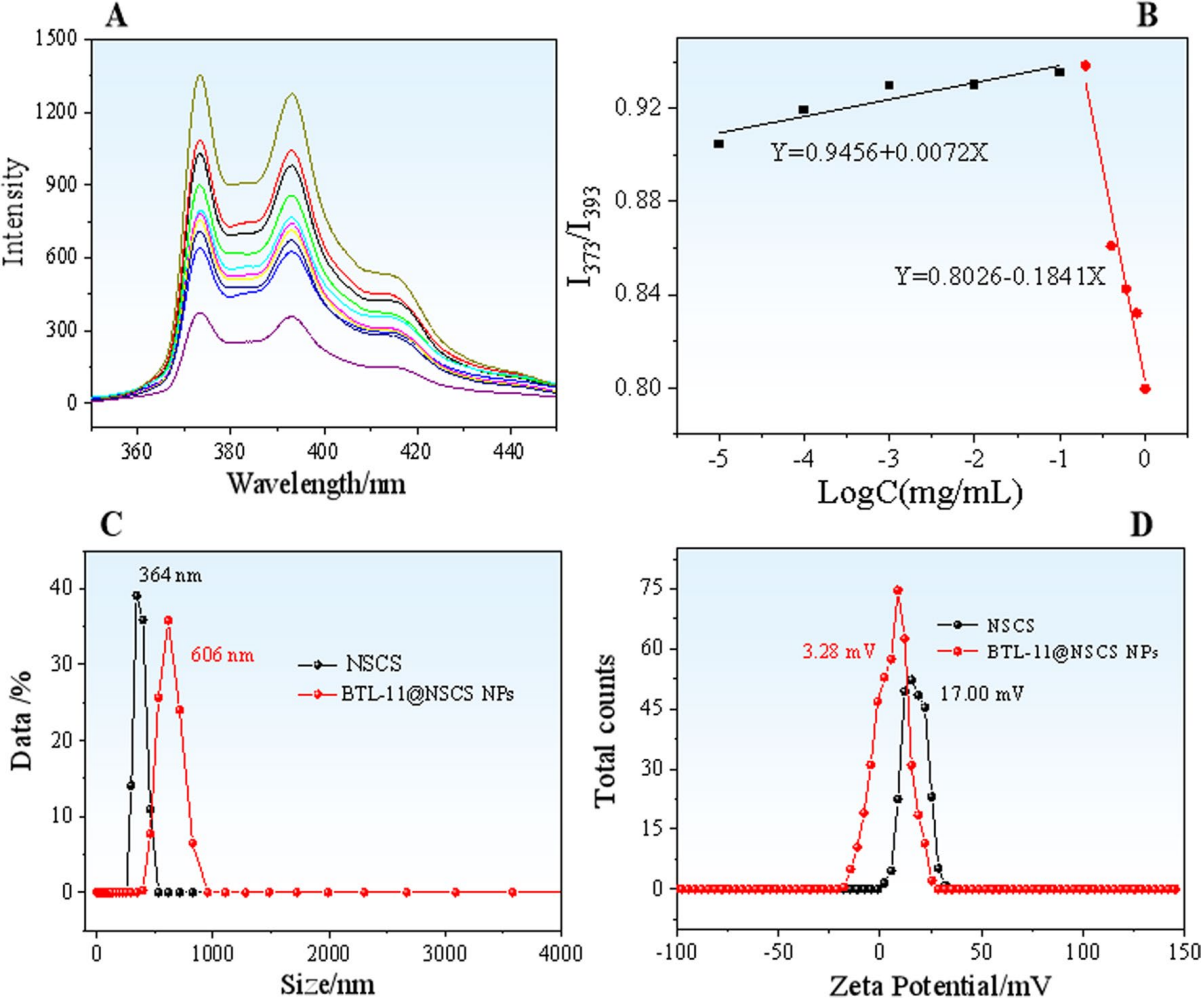
The fluorescence spectra of pyrene in aqueous NSCS solutions with different concentrations from 10 mg/mL to 1.0 mg/mL (**A**); The relationship between logarithm of concentration and peak ratio (**B**). Size distribution (**C**) and zeta potential (**D**) of NSCS and BTL-11@NSCS NPs (in liquid)

CMC is the lowest concentration of surfactant in water or other solvents to form micelles [63]. In this study, pyrene was used as a fluorescent probe to detect the CMC value of NSCS. The solubility of pyrene in water was very low. Amphiphilic copolymer NSCS had solubilizing effect on nonpolar organic compounds. With the continuous change of concentration gradient, the solubilizing ability of the copolymer NSCS to pyrene changes constantly. When the polymer forms micelles in water, pyrene could quickly transfer from the hydrophilic environment to the hydrophobic core of the micelles, resulting in the change of fluorescence absorption [64]. When the copolymer concentration increased to a certain value, the fluorescence peak ratio changed sharply, which proved the formation of copolymer micelles. The smaller the CMC value, the more stable the polymer was in aqueous solution [65]. Figure 3A showed the fluorescence spectra of pyrene with the same concentration in different concentrations of NSCS. The illustration demonstrated the relationship between the ratio of the fluorescence intensity of the first and third vibrational bands (I_373_/I_393_) in the pyrene emission spectrum and the concentration of NSCS. The curve showed that the ratio of I_373_/I_393_ increased slowly at first, and then decreased sharply (Fig. 3B). The CMC of NSCS in water was determined to be 0.1788 mg/mL.

### Particle size and zeta-potential analyses

The particle size of nanoparticles in aqueous solutions was analyzed by using dynamic light scattering technology, the particle size distribution curve was shown in Fig. 3C. The particle size range of NSCS was from 255 to 450 nm, with an average particle size of 364 nm. The particle size range of BTL-11@NSCS NPs was from 390 to 1000 nm, with an average particle size of 606 nm. Compared with NSCS, the nanoparticle distribution of BTL-11@NSCS NPs has widened, and the average particle size was also much larger, mainly due to the adsorption and embedding of BTL-11 in NSCS.

The zeta potential curves of NSCS and BTL-11@NSCS NPs were exhibited in Fig. 3D. It can be seen that the potentials of NSCS and BTL-11@NSCS NPs were 3.28 and 17.00 mV, respectively, indicated that both surfaces carry positive charges. This was mainly the result of NH_2_ ionization of CS, which was consistent with literature reports. These positively charged nanoparticles on the surface were conducive to the stable existence of nanoparticles over a longer period of time due to electrostatic repulsion. These positively charged nanoparticles on the surface were conducive to the stable existence of nanoparticles over a longer period of time due to electrostatic repulsion.

### Studies of loading content (LC) and encapsulation efficiency (EE)

LC and EE are two important indicators to evaluate the pesticide loading ability of the delivery system. In this study, the peak area was used for linear regression of pesticide concentration. The results showed that under this chromatographic condition, the linear relationship was good in the range of 0.002–0.2 mg/mL, and the LC and EE were shown in Additional file 1: Table S1. It was obvious that LC and EE increase with the decrease of the ratio of NSCS to BTL-11, and EE was better. However, compared with EE, the increase of LC was more obvious. This was mainly because there were many hydrophilic segments on the surface of NSCS, which could generate hydrogen bonds with BTL-11. In this way, BTL-11 will not only be embedded in the hydrophobic interior of NSCS, but also be dispersed on the surface of nanoparticles, and BTL-11 molecules will also be bound in its hydrophilic molecular chain. These results clearly illustrated the successful surface decoration of NSCS in BTL-11 and that NSCS does not impact drug loading of the final formulation.

### Drug controlled release and phytotoxicity tests

**Fig. 4:**
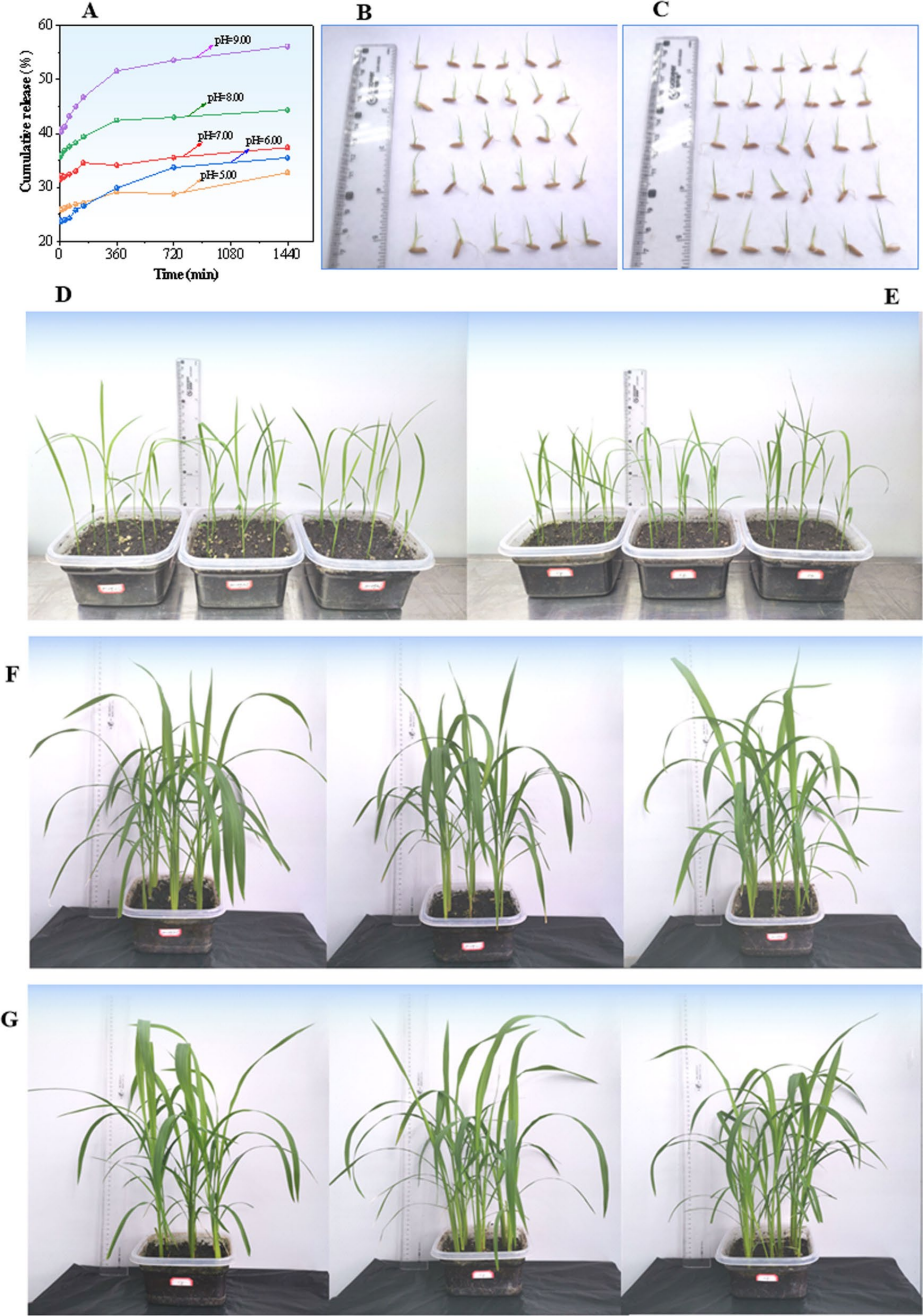
Cumulative release of BTL-11 from BTL-11@NSCS at different pH values (**A**); Phytotoxicity studies after the spraying of BTL-11@NSCS NPs; **B**, **D**, and **F** are after spraying of BTL-11@NSCS NPs, the rice plants were cultured for different periods; **C**, **E**, and **G** is the blank groups without drugs

In vitro drug release was studied for BTL-11@NSCS NPs by using the ultraviolet spectrophotometry. The release profiles as a function of time from the different formulations were shown in Fig. 4A. As expected, BTL-11@NSCS NPs showed rapid release over the initial 360 min followed by a slower release. The BTL-11@NSCS NPs released 44% of BTL-11 in the 1440 min at pH = 8.0 (Fig. 4A). The BTL-11@NSCS NPs released 56% of BTL-11 content over a period of 1440 min with 45% of the release occurring within the first 100 min at pH = 9.0 (Fig. 4A). This may be due to the significant influence of pH on nanoparticles. The effect phytotoxicity of BTL-11@NSCS NPs on rice can be clearly seen from Fig. 4. Interestingly, rice sprouts uniformly and seedlings grow vigorously after uniform spraying of BTL-11@NSCS NPs with concentration of 500 μg/mL, the potential phytotoxicity was not observed (Fig. 4B, D). Also, the repeated spraying operations for BTL-11@NSCS NPs could not contribute to the possible phytotoxicity (Fig. 4F), confirming that the designed nanoagricultural complex was safe during application.

### Biological assay against phytopathogenic fungi

**Fig. 5:**
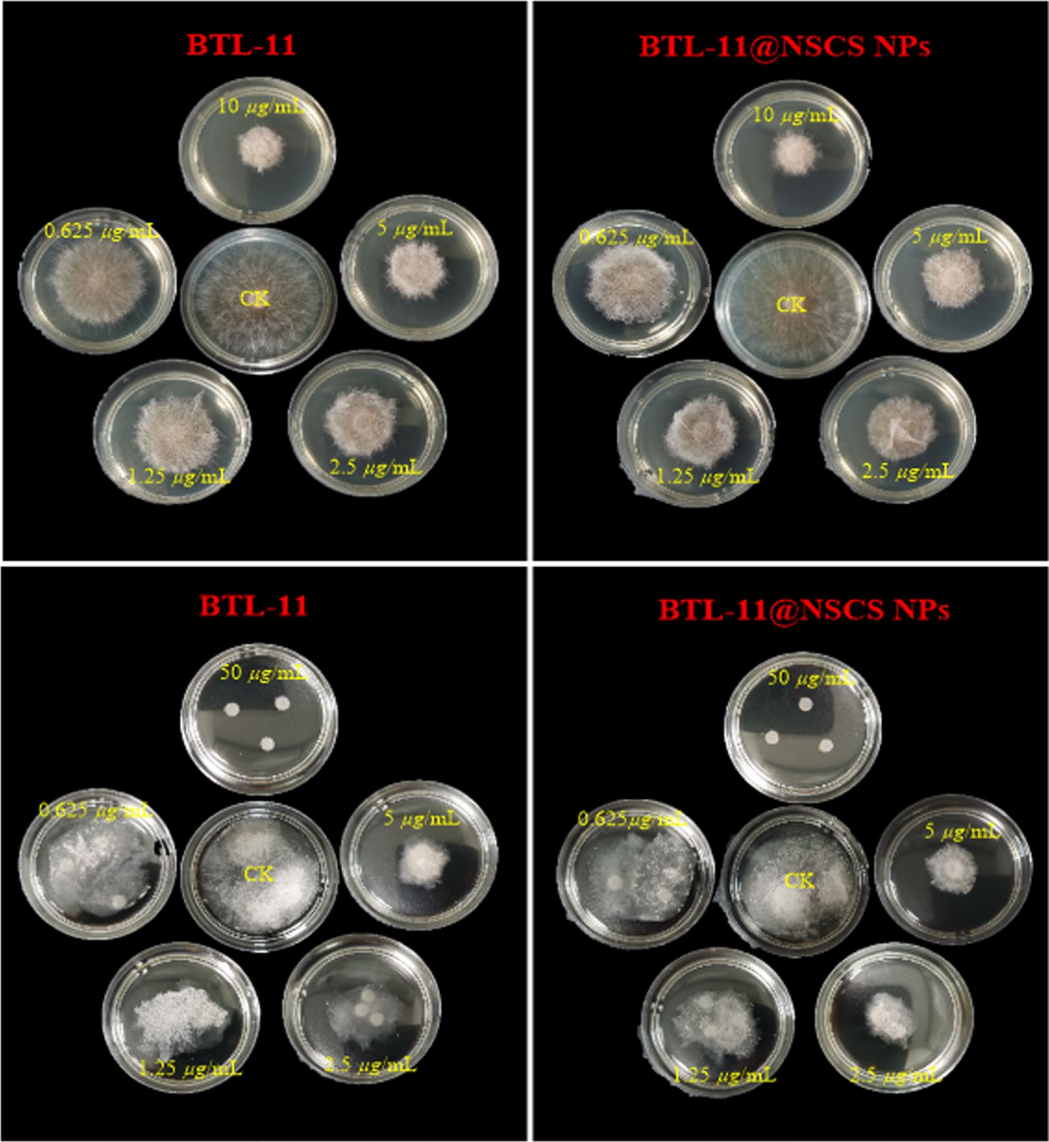
Anti-fungal effects of BTL-11 and BTL-11@NSCS NPs on *R. solani* under different media conditions

Preliminary bioassay results (Additional file 1: Table S2) revealed that most target compounds exhibited pretty good anti-fungal activities against *R. solani*, *Phytophthora capsici (P. capsici)*, *Botrytis cinerea* (*B. cinerea*), and *Sclerotinia sclerotiorum* (*S. sclerotiorum*). Significantly, BTL-11 showed the most prominent inhibitory activity against *R. solani* (Fig. 5) compared with *P. capsici*, *B. cinerea*, and *S. sclerotiorum* at 10 μg/mL. Consequently, the half maximal effective concentration (EC_50_) values of the target compounds against *R. solani* was further assessed by using serial dilution. As depicted in Table 1, it can be clearly visualized that the activity of BTL-11@NSCS NPs (EC_50_ = 0.7 μg/mL) was significantly improved, which was better than that of the agricultural fungicide azoxystrobin (EC_50_ = 11.1 μg/mL). It was therefore extrapolated that BTL-11@NSCS NPs can activate or increase the anti-fungal activity.

**Table 1:** The EC_50_ values of target compounds against *R. solani*^A^

Compounds	R	Regression equation	r^2^	EC_50_ (µg/mL)
BTL-1	H	y = 1.4403x + 3.3581	0.9932	13.8 i,j
BTL-2	4-F	y = 1.2104x + 3.6327	0.9630	13.4 j
BTL-3	4-Cl	y = 1.3063x + 3.8144	0.9856	7.3 p
BTL-4	4-Br	y = 1.2214x + 3.6116	0.9832	13.7 i,j
BTL-5	4-CH_3_	y = 1.1803x + 3.2980	0.9966	27.6 e
BTL-6	4-OCH_3_	y = 0.9718x + 3.7821	0.9646	17.9 h
BTL-7	5-F	y = 2.4766x + 3.9060	0.9951	2.7 r
BTL-8	5-Cl	y = 2.7841x + 2.6106	0.9671	7.2 p
BTL-9	5-Br	y = 1.3326x + 3.8441	0.9921	9.8 n
BTL-10	6-F	y = 1.1538x + 3.7625	0.9981	11.8 k,l
BTL-11	6-Cl	y = 0.7749x + 4.8997	0.9869	1.3 s
BTL-11@NSCS NPs	–	y = 0.4459x + 5.0719	0.9982	0.7 s
BTL-12	6-Br	y = 1.4780x + 3.6367	0.9801	8.3 o
BTL-13	6-NO_2_	y = 1.1031x + 3.5184	0.9849	22.0 g
BTL-14	6-CH_3_	y = 1.2208x + 3.6662	0.9966	12.3 k
BTL-15	6-CF_3_	y = 1.1606x + 3.8543	0.9940	9.7 n
BTL-16	6-OCH_3_	y = 0.9079x + 3.6190	0.9944	33.1 d
BTL-17	6-OCF_3_	y = 1.4334x + 3.5432	0.9527	10.3 m,n
BTL-18	6-COCH_2_CH_3_	y = 1.0012x + 3.5904	0.9657	25.5 f
BTL-19	6-COOCH_3_	y = 1.2274x + 3.2635	0.9671	25.9 f
BTL-20	6-OH	–	–	> 100 a
BTL-21	6-SOOCH_3_	y = 1.0350 x + 3.8088	0.9655	14.1 i
BTL-22	4,6-di-F	y = 0.8438x + 4.3754	0.9728	5.4 q
BTL-23	4,6-di-Cl	y = 2.4080x + 4.0733	0.9813	2.4 r
BTL-24	4,6-di-CH_3_	y = 0.7123x + 3.6862	0.9950	69.9 c
Fluopyram	–	y = 1.4736x + 2.1763	0.9508	82.4 b
Azoxystrobin	–	y = 0.7673x + 4.1994	0.9735	11.1 l,m

^A^The experiments were repeated 3 times, *P* < 0.05

### Inhibition of sclerotinia formation and germination

**Fig. 6:**
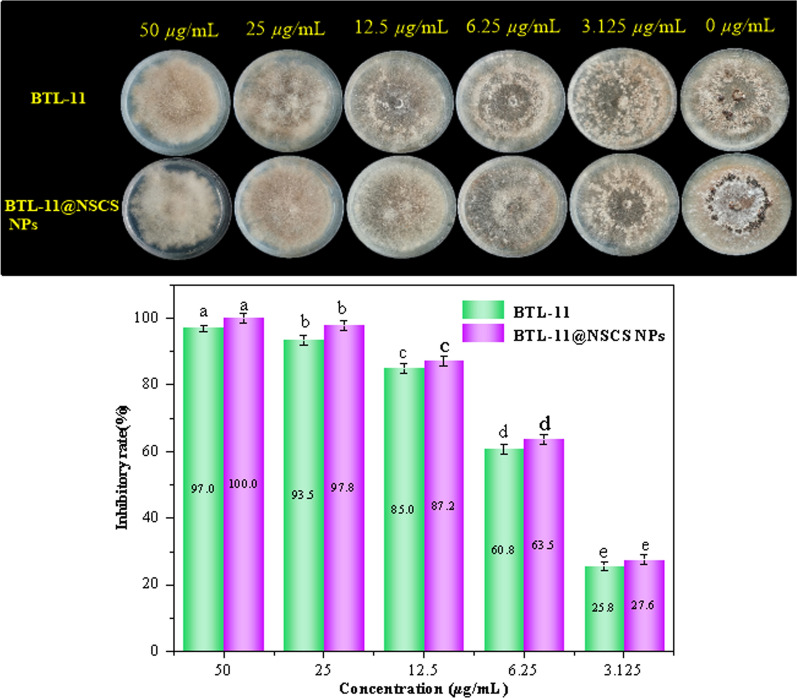
Inhibitory activity of BTL-11 and BTL-11@NSCS NPs on the formation of sclerotia of *R. solani*

**Fig. 7:**
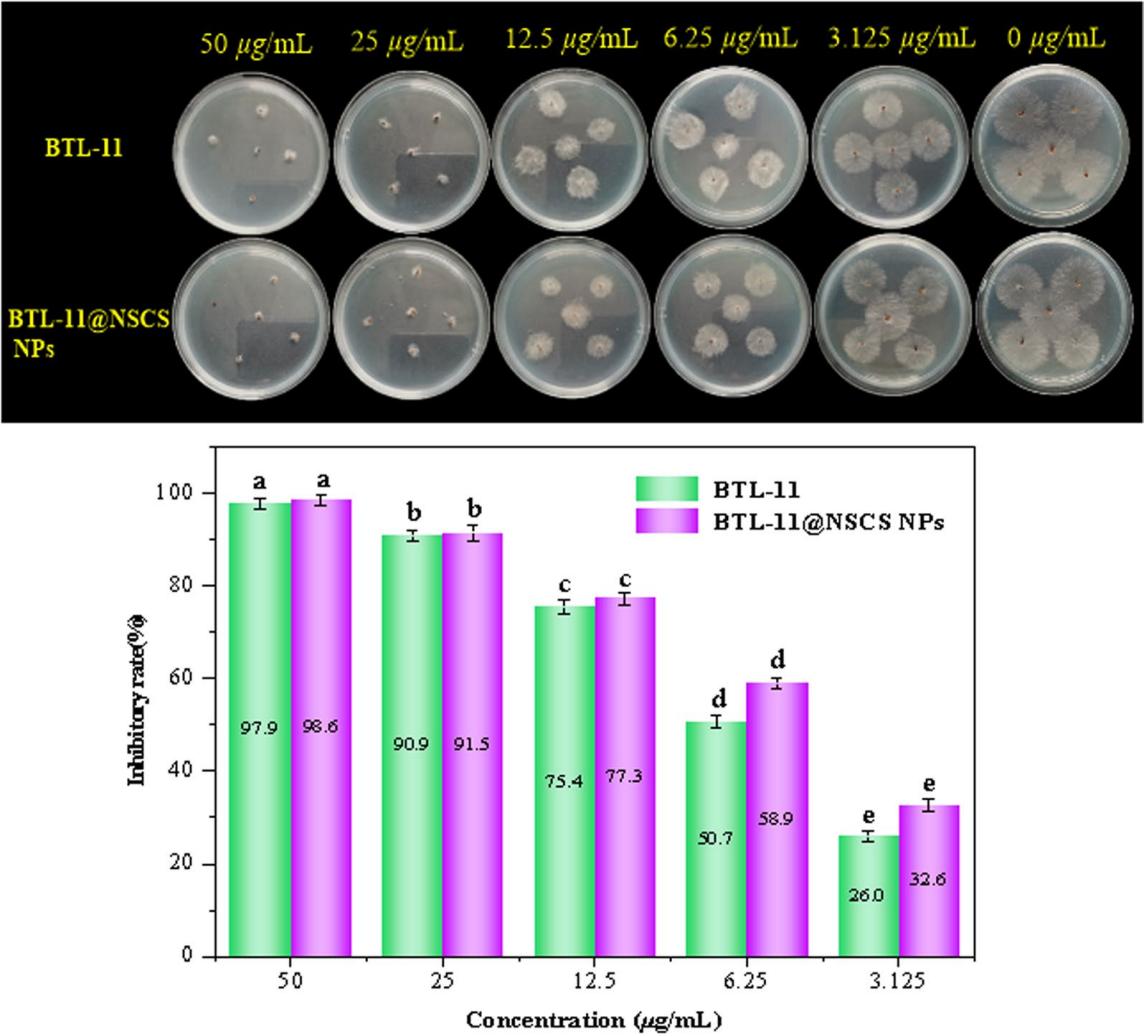
Inhibitory activity of BTL-11 and BTL-11@NSCS NPs on lycopene sclerotic germination. of *R. solani*

The effect on the sclerotinia formation and germination of *R. solani* were presented in Figs. 6 and 7. BTL-11@NSCS NPs showed excellent inhibitory effect on sclerotia formation of *R. solani* were 100.0 and 27.6% at 50 and 3.125 μg/mL. However, the BTL-11 showed a poor activity at the same concentration, and the values were 97.0 and 25.8%, respectively. Moreover, BTL-11@NSCS NPs effectively inhibited the germination of sclerotia were 98.6 and 32.6% at 50 and 3.125 μg/mL, which greatly exceeded the inhibitory activity of BTL-11 on germination of sclerotia. The above experimental testes displayed that BTL-11@NSCS NPs could effectively inhibit, reduce, and spread the sclerotia formation and germination of *R. solani* infection.

### Morphological testing of *R. solani*

**Fig. 8:**
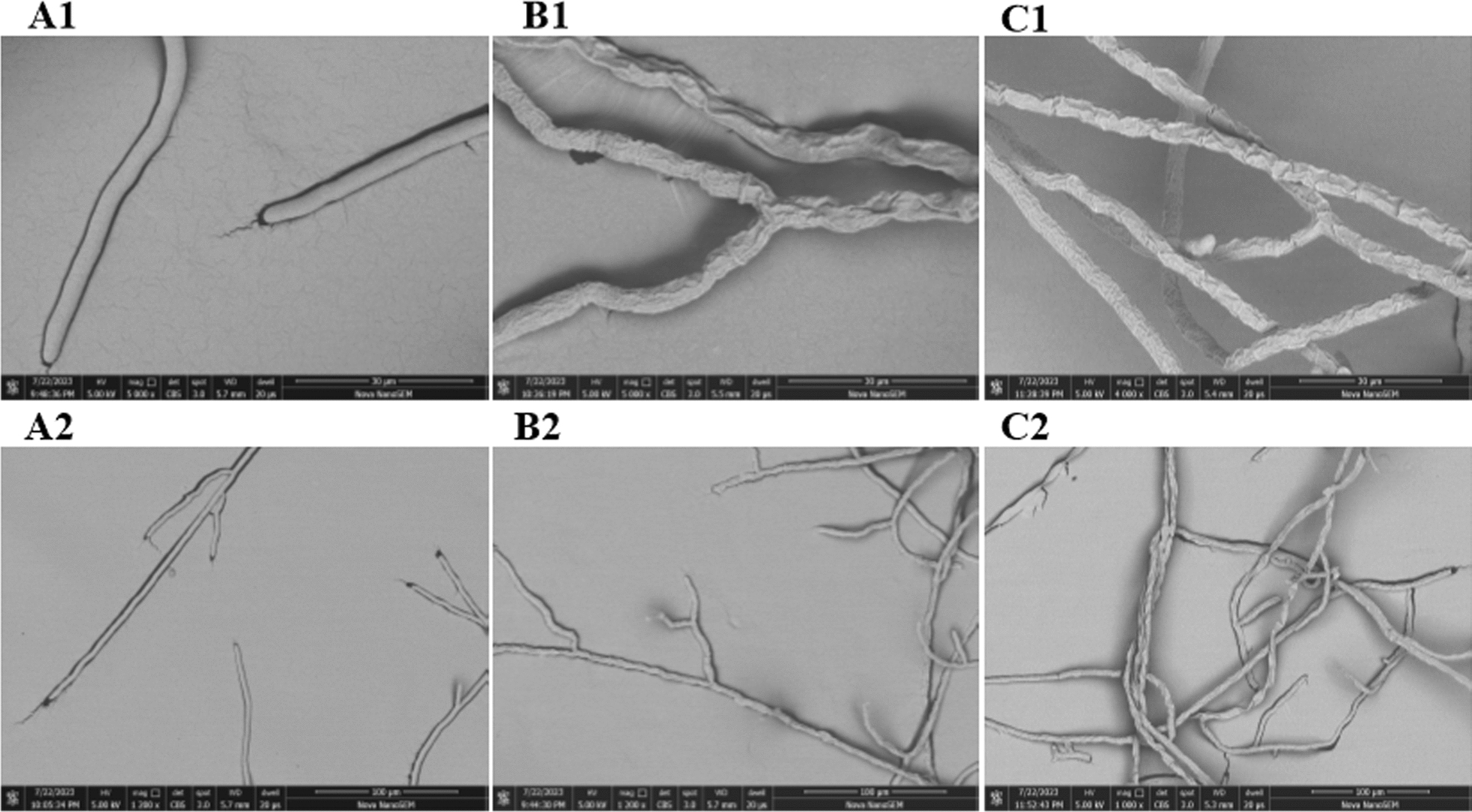
The morphological changes of *R. solani* under SEM (after 12 h). **A1**, **A2**: CK (0 μg/mL), **B1**: BTL-11 (20 μg/mL); **B2**: BTL-11 (5 μg/mL); **C1**: BTL-11@NSCS NPs (20 μg/mL); **C2**: BTL-11@NSCS NPs (5 μg/mL); scale for 30 and 100 µm

The changes of BTL-11 and BTL-11@NSCS NPs on the mycelic morphology of *R. solani* was examined on LM (Additional file 1: Fig. S1) and SEM (Fig. 8). Control check (CK) hyphae of *R. solani* (Additional file 1: Fig. S1 A-1, A-2; Fig. 8 A1, A2) were well grown, elongated, homogeneous, and well aligned. With BTL-11 (Additional file 1: Fig. S1 B-1, B-2; Fig. 8 B1, B2) and BTL-11@NSCS NPs (Additional file 1: Fig. S1 C-1, C-2; Fig. 8 C1, C2) treatment, there were an increased number of hyphal branches, short, thick, and the appearance of dry folds or even ulcers, respectively. Briefly, those finding suggested that BTL-11 and BTL-11@NSCS NPs may interfere with cell wall synthesis of *R. solani*, leading to severe disruption of the cell membrane structure.

### Disruption of the *R. solani* cell wall

**Fig. 9:**
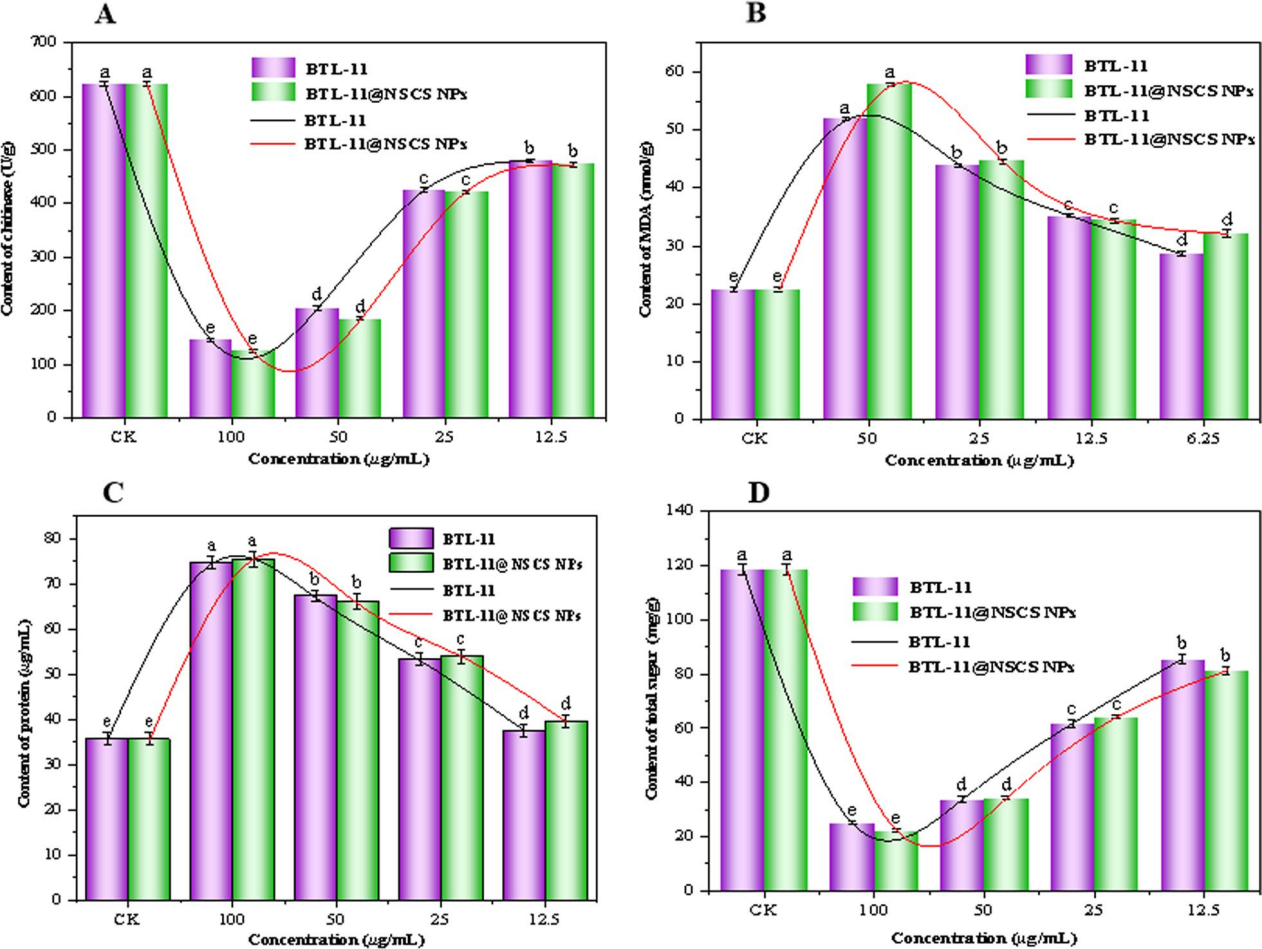
Effect of different concentrations of BTL-11 and BTL-11@NSCS NPs on chitinase (**A**), MDA (**B**), protein (**C**), and total sugar (**D**) content of *R. solani*

The Additional file 1: Table S3 and Fig. 9A revealed that the content of chitinase decreased when the concentration increased, which displayed that the compounds at high concentration hindered the normal development of the mycelium of *R. solani* and impeded mycelial growth. Moreover, the content of chitinase for BTL-11@NSCS NPs was 125.4 *U*/g at 100 μg/mL, which was much lower than that of BTL-11 (145.0 *U*/g) for chitinase activity. It could be speculated that the BTL-11@NSCS NPs could destroy the cell wall structure of *R. solani* by inducing the hydrolysis of chitin in the cell wall to produce *N*-acetylglucosamine.

### Impact on *R. solani* cell membrane permeability

As shown in Fig. 9B and Additional file 1: Table S4, the malondialdehyde (MDA) content of rice blast fungus showed a significant increase with the treatment concentration of medication. The findings indicated that BTL-11 and BTL-11@NSCS NPs were highly susceptible to causing increased lipid peroxidation of *R. solani* cell membrane, destroyed the cell membrane, and then inhibited or even killed the rice blast cells.

The protein content increased with the increase of agent concentration (Fig. 9C, Additional file 1: Table S5). This may be due to the fact that when subjected to adversities such as high concentrations of BTL-11 and BTL-11@NSCS NPs, rice blast fungi could increase the cellular defense against adverse environments by enhancing their own metabolisms, and synthesizing soluble proteins ability, respectively.

The change in total sugar content showed a highly significant decreasing trend (Fig. 9D, Additional file 1: Table S6), compared to the CK, which decreased by 78.8, 81.2% at 100 μg/mL, respectively. These consequences revealed that BTL-11 and BTL-11@NSCS NPs could inhibit sugar synthesis and metabolism, reduce the ability of fungal cells to utilize nutrients and ultimately affecting the normal growth of *R. solani*.

**Fig. 10:**
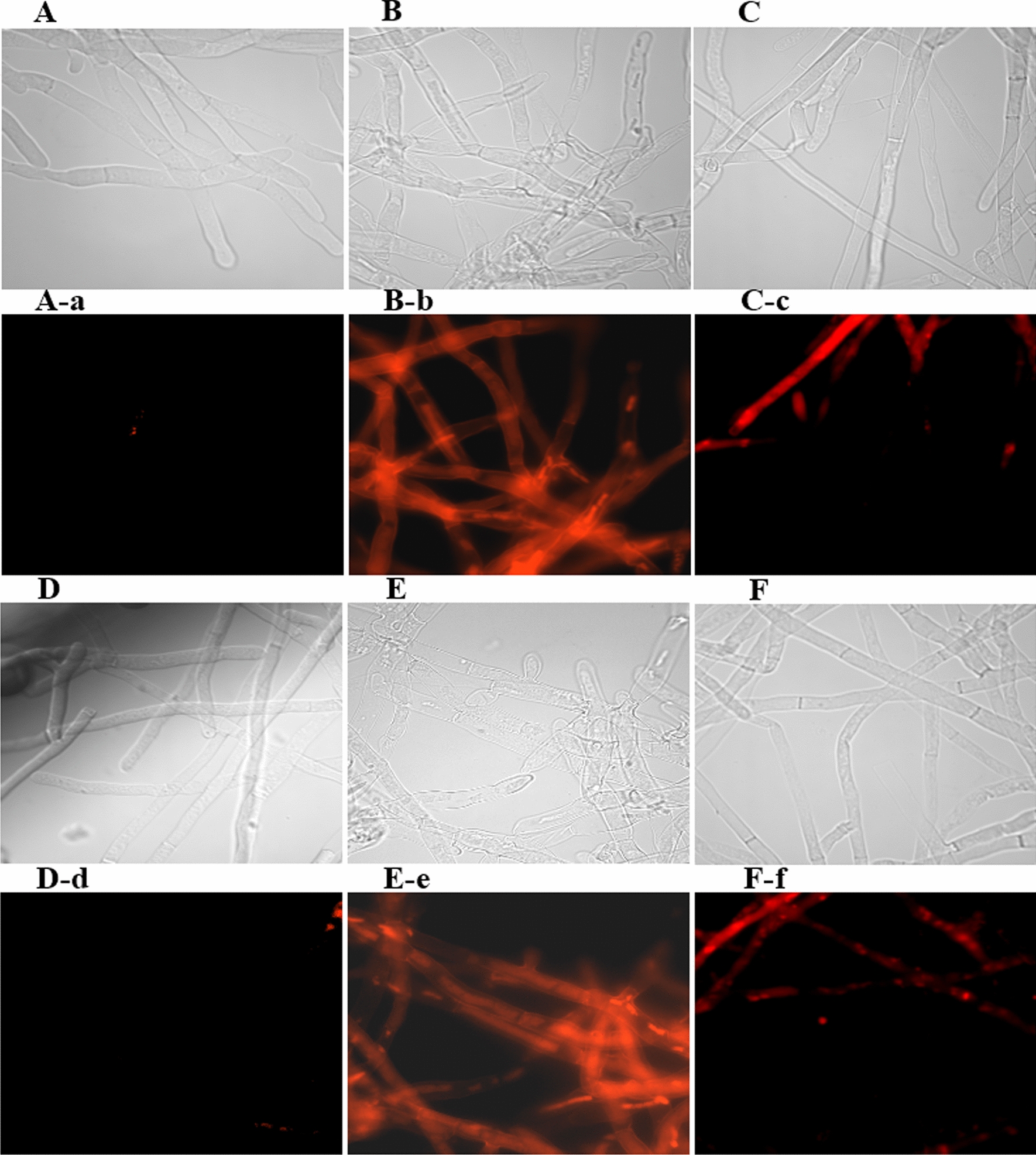
Effect of BTL-11 and BTL-11@NSCS NPs on membrane integrity of *R. solani*. **A, A-a**: CK; **B, B-b**: BTL-11 (20 μg/mL); **C, C–c**: BTL-11 (5 μg/mL); **D, D-d**: CK; **E, E-e**: BTL-11@NSCS NPs (20 μg/mL); **F, F-f**: BTL-11@NSCS NPs (5 μg/mL); magnification: 100 × 10; scale for 10 µm

In order to clarify the destructive effects of BTL-11 and BTL-11@NSCS NPs on the cell membrane of *R. solani* (Fig. 10), fluorescent dye staining was performed after the rice blast fungus was treated with different concentrations of compounds for 12 h. The findings revealed that the morphology of mycelium was severely changed after being treated with BTL-11 (Fig. 10B, B-b) and BTL-11@NSCS NPs (Fig. 10E, E-e) at 20 μg/mL, which exhibited severe changes in mycelial morphology, with pleated hyphae, and intracellular agglutination. Whereas, the hyphae were well-grown, and a small amount of cytoplasmic unevenness of the mycorrhizal body occurred (Fig. 10C, C-c, F, F-f) at 5 μg/mL. The above results indicated that the treatment of rice blast fungus with BTL-11 and BTL-11@NSCS NPs caused cell membrane disruption and increased cell membrane permeability.

### Effect on growth and respiratory energy metabolism in rice sheath blight

**Fig. 11:**
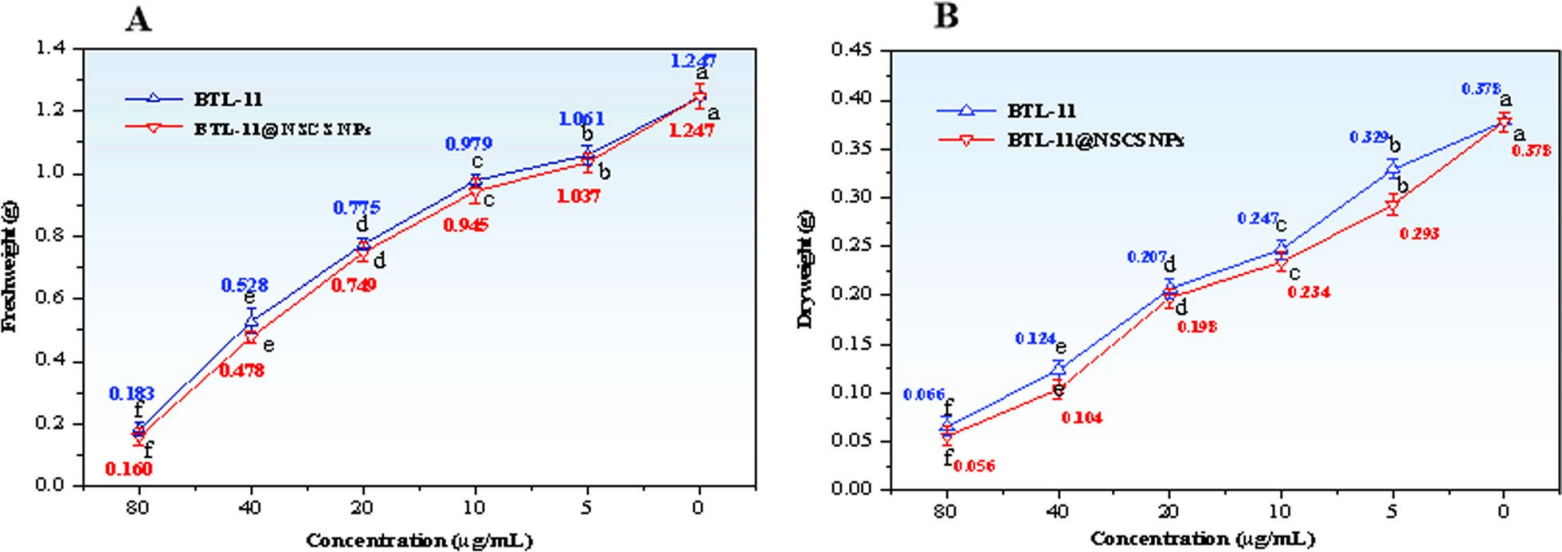
Effect of BTL-11 and BTL-11@NSCS NPs on the amount of mycelial growth of *R. solani*

After being treated with different concentrations of BTL-11 and BTL-11@NSCS NPs, the mycelial volume of *R. solani* was significantly different from that of the CK, and the inhibition rate increased with the increase of concentration (Fig. 11A, B). Those showed that BTL-11 and BTL-11@NSCS NPs could effectively enter into the intracellular to interfere with the growth of the fungus, which in turn affected the normal growth of rice blast fungus, thus achieving the effect of fungal inhibition.

### In vivo trials against rice sheath blight disease

**Fig. 12:**
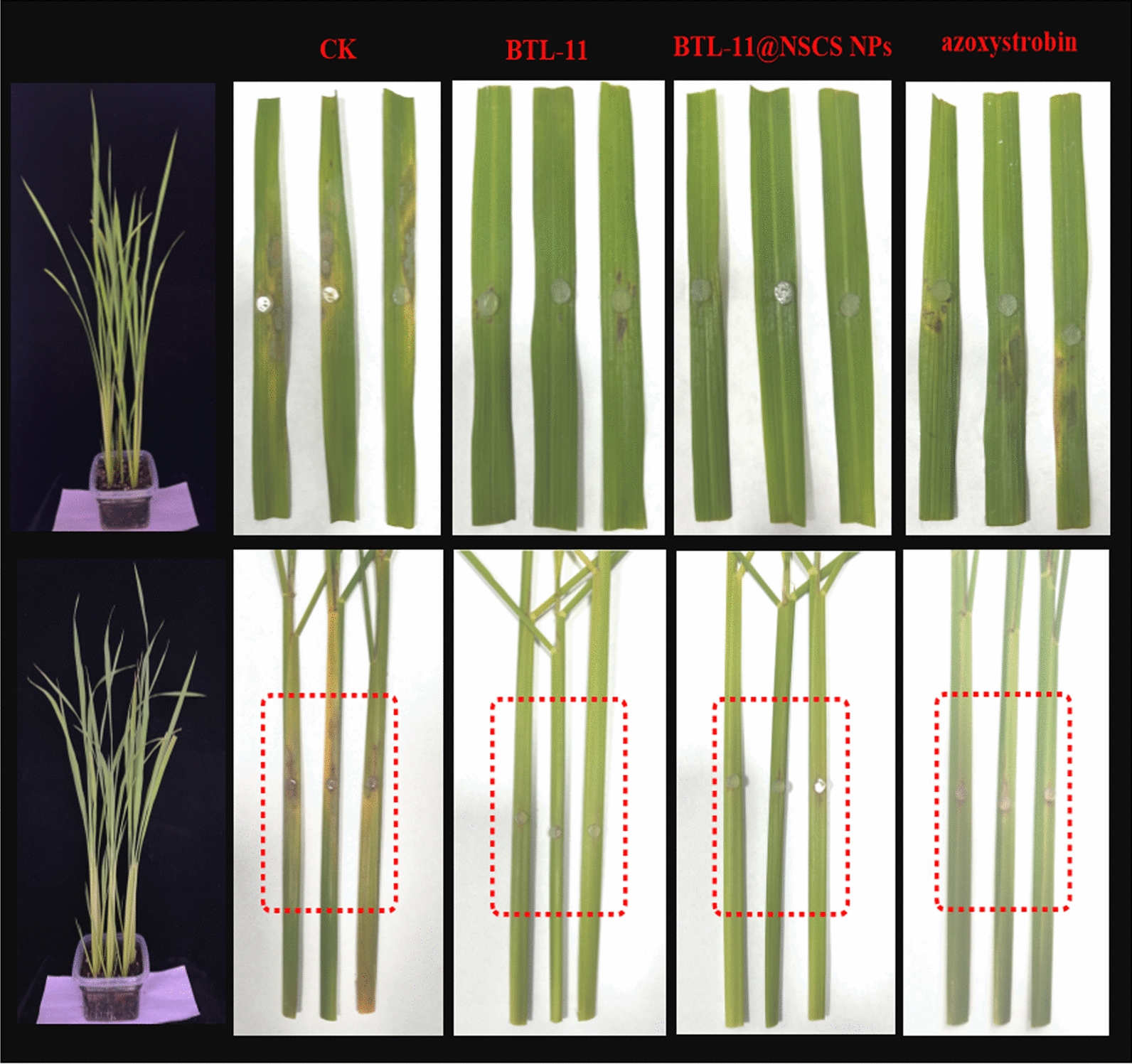
In vivo protective effect of BTL-11 and BTL-11@NSCS NPs against *R. solani* using leaves and sheathes of cultivated rice at 200 μg/mL

Preliminary in vitro inhibitory activity and mechanism of action experiments had demonstrated that BTL-11 and BTL-11@NSCS NPs exhibited the favorable inhibitory activity against *R. solani*, which was further explored to investigate their inhibitory activities against the pathogens in vivo. As displayed in Table 2 and Fig. 12, the protection efficiency of BTL-11 for cultivated rice leaf and sheath was 79.6 and 76.5%, respectively. By contrast, the BTL-11@NSCS NPs anti-fungal ability was strongly released and afforded significant control efficiencies of 85.9 and 81.1% at 200 μg/mL. Those effects were significantly better than those of the agricultural fungicide azoxystrobin (51.5 and 66.5%). Furthermore, the *R. solani* symptoms were distinctly alleviated from the whole perspective, manifesting that a nanoencapsulated pesticides fungicide for managing fungal infections was probably developed with improved safety.

**Table 2:** In vivo protective activity against *R. solani* at 200 µg/mL^A^

Treatment	leaf of rice^B^		sheath of rice^B^	
	Lesion length (mm)	Control efficacy (%)	Lesion length (mm)	Control efficacy (%)
BTL-11	8.7 ± 1.1c	79.6	10.7 ± 1.2c	76.5
BTL-11@NSCS NPs	6.0 ± 0.6d	85.9	8.6 ± 1.1d	81.1
azoxystrobin	20.7 ± 1.6b	51.5	15.3 ± 1.2b	66.5
CK	42.7 ± 1.3a	–	45.7 ± 2.3a	–

^A^The experiments were repeated 3 times, *P* < 0.05

^B^Values are the mean ± SD of 12 leaves

### Molecular docking of BTL-11 with oxidoreductase of *R. solani*

In the present work, the systematically structural optimizations focused on benzothiazolamide structures strikingly generated the promising candidate BTL-11 that exhibited the nonnegligible inhibitory effect against *R. solani *in vitro and in vivo. Subsequently, the molecular docking of the bioactive molecules BTL-11 and fluopyram with oxidoreductase was conducted to explore their differences in binding modes.

**Fig. 13:**
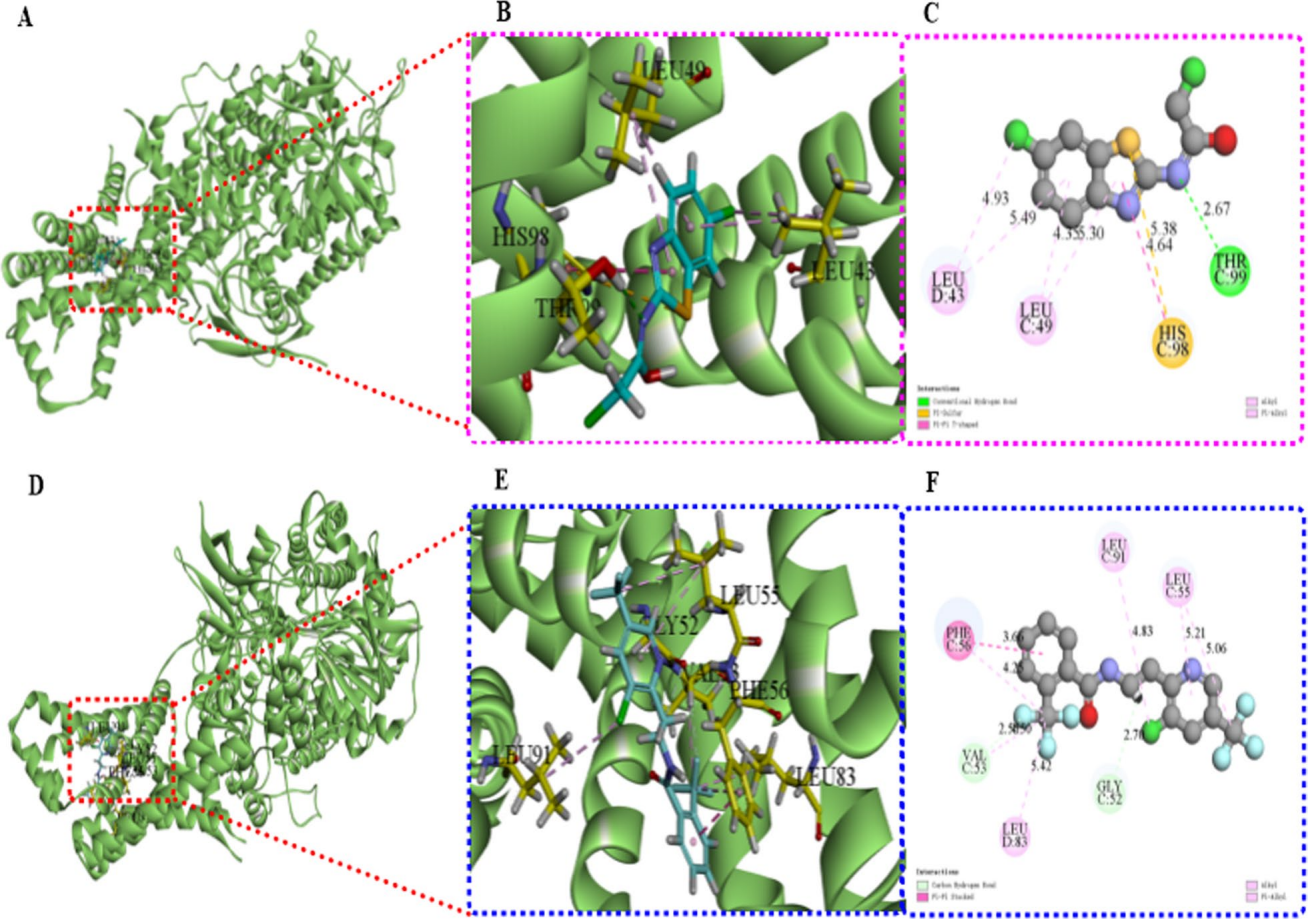
Docking analyses between BTL-11 (**A, B, C**) or fluopyram (**D, E, F**) and oxidoreductase of *R. solani*

As can be seen from Fig. 13, the bioactive molecules BTL-11 and fluopyram were well-embedded in the active protein pocket on oxidoreductase via approximately the same conformations that roughly gave them similar interactions with most surrounding residues [61]. For example, THR 99 formed a strong hydrogen bond with the emerged as hydrogen-bond donors in BTL-11 (distance = 2.67 Å), GLY formed a hydrogen bond with the emerged as hydrogen bond donors fluopyram (distance = 2.70 Å). Concurrently, the 6-chlorobenthiazole fragment of the title BTL-11 was linked to LEU 43 (distance = 4.93 and 5.49 Å), LEU 49 (distance = 4.35 and 5.30 Å), and HIS 98 (distance = 4.64 and 5.38 Å) residues to form multiple interactions, the trifluoromethyl pyrazole/benzene fragment of fluopyram connected with the LEU 55 (distance = 5.06 and 5.21 Å) and PHE 56 (distance = 3.66 and 4.25 Å) residues also formed multiple interactions. Among them, the HIS is one of the most important amino acid residues within the active pocket of the oxidoreductase, and it was not present in the interaction pattern of fluoropyran. To some extent, the above mentioned similar interactions of the bioactive molecule BTL-11 and fluopyram with oxidoreductase crucial residues might be the underlying factor that maintained their anti-fungal activities against phytopathogenic microorganisms. The above molecular docking results provided an important basis for the feasibility of the designed benzothiazole amides derivatives as potential biocides substitutes.

## Conclusions

Our study showed that BTL-11@NSCS NPs pesticides displayed good fluidity, solubility, and a high drug loading. In addition, the sustained release profile of BTL-11@NSCS NPs can enhance the drug’s protective effectiveness. No cytotoxic effects were observed for BTL-11@NSCS NPs suggesting their safety for using in crop growth. This suggests showed that a viable polymeric nanocarrier technology that can be utilized for the development of bio-compatible and safe agricultural fungicides. Notably, as depicted in Additional file 1: Table S1 and Additional file 1: Fig. S2, compared with BTL-11, BTL-11@NSCS NPs has a small, uniform, stable morphology feature, and outstanding physicochemical properties, thereby contributing to satisfactory anti-fungal activity. The solubility bio-activity of the bioactive molecule BTL-11 was enhanced by carrier encapsulation, which solves the problem of small molecules for bio-agricultural applications to a certain extent. The mechanism of action studies suggested that BTL-11@NSCS NPs could inhibit or even kill *R. solani* cells by changing mycelial morphology, destroying the cell wall, affecting the intensification of intracellular lipid peroxidation, and influencing protein synthesis and total sugar content, and then affecting rice sheath blight fungi lesion cells. According to a pot test on *R. solani*, BTL-11@NSCS NPs significantly reduced *R. solani* symptoms, with a control effect of 85.9% at 200 μg/mL, which was significantly better than that of azoxystrobin (51.5%). In the light of the foregoing investigations, we expected that green, safe, eco-friendly, and bio-compatible nanopesticides can be used for plant disease control.

### Supplementary information

Supplementary data including Additional file 1: Table S1–S7, Additional file 1: Fig. S1–S57 and characterization of target compounds BTL-1–BTL-24.

### Supplementary Information

**Additional file 1.** Table S1. Loading content (LC) and encapsulation efficiency (EE)^a^.Table S2. In vitro activity of BTL-1−BTL-24 at 10 μg/mL against *R. solani, P. capsici, B. cinerea, and S.sclerotiorum*^A^. Table S3. Effect of BTL-11 and BTL-11@NSCS NPs on chitinase content of *R. solani*^A^. Table S4. Effect of BTL-11 and BTL-11@NSCS NPs on MDA content of *R. solani*^A^. Table S5. Effect of BTL-11 and BTL-11@NSCS NPs on protein content of *R. solani*^A^. Table S6. Effect of BTL-11 and BTL-11@NSCS NPs on total sugar content of *R. solani*^A^. Table S7. X-ray single crystal data of compound BTL-8. Fig. S1 Morphology of mycelia *R. solani* treated with BTL-11 and BTL-11@NSCS NPs at 20 μg/mL. A-1, A-2: CK; B-1, B-2: BTL-11; C-1, C-2: BTL-11@NSCS NPs; magnification: 10×10; scale for 10 μm. Fig. S2 Photographs of solutions of NSCS 2 mg/mL in water, BTL-11@NSCS NPs: BTL-11 2 mg/mL in NSCS solution, BTL-11 2 mg/mL in water. Fig. S3 X-ray single crystal structure of compound BTL-8. Fig. S4 ^1^H NMR for compound BTL-1. Fig. S5 ^13^C NMR for compound BTL-1. Fig. S6 ^1^H NMR for compound BTL-2. Fig. S7 ^13^C NMR for compound BTL-2. Fig. S8 ^19^F NMR for compound BTL-2. Fig. S9 ^1^H NMR for compound BTL-3. Fig. S10 ^13^C NMR for compound BTL-3. Fig. S11 ^1^H NMR for compound BTL-4. Fig. S12 ^13^C NMR for compound BTL-4. Fig. S13 ^1^H NMR for compound BTL-5. Fig. S14 ^13^C NMR for compound BTL- Fig. S15 ^1^H NMR for compound BTL-6. Fig. S16 ^13^C NMR for compound BTL-6. Fig. S17 ^1^H NMR for compound BTL-7. Fig. S18 ^13^C NMR for compound BTL-7. Fig. S19 ^19^F NMR for compound BTL-7. Fig. S20 ^1^H NMR for compound BTL-8. Fig. S21 ^13^C NMR for compound BTL-8. Fig. S22 ^1^H NMR for compound BTL-9. Fig. S23 ^13^C NMR for compound BTL-9. Fig. S24 ^1^H NMR for compound BTL-10. Fig. S25 ^13^C NMR for compound BTL-10. Fig. S26 ^19^F NMR for compound BTL-10. Fig. S27 ^1^H NMR for compound BTL-11. Fig. S28 ^13^C NMR for compound BTL-11. Fig. S29 ^1^H NMR for compound BTL-12. Fig. S30 ^13^C NMR for compound BTL-12. Fig. S31 ^1^H NMR for compound BTL-13. Fig. S32 ^13^C NMR for compound BTL-13. Fig. S33 ^1^H NMR for compound BTL-14. Fig. S34 ^13^C NMR for compound BTL-14. Fig. S35 ^1^H NMR for compound BTL-15. Fig. S36 ^13^C NMR for compound BTL-15. Fig. S37 ^19^F NMR for compound BTL-15. Fig. S38 ^1^H NMR for compound BTL-16. Fig. S39 ^13^C NMR for compound BTL-16. Fig. S40 ^1^H NMR for compound BTL-17. Fig. S41 ^13^C NMR for compound BTL-17. Fig. S42 ^19^F NMR for compound BTL-17. Fig. S43 ^1^H NMR for compound BTL-18. Fig. S44 ^13^C NMR for compound BTL-18. Fig. S45 ^1^H NMR for compound BTL-19. Fig. S46 ^13^C NMR for compound BTL-19. Fig. S47 ^1^H NMR for compound BTL-20. Fig. S48 ^13^C NMR for compound BTL-20. Fig. S49 ^1^H NMR for compound BTL-21. Fig. S50 ^13^C NMR for compound BTL-21. Fig. S51 ^1^H NMR for compound BTL-22. Fig. S52 ^13^C NMR for compound BTL-22. Fig. S53 ^19^F NMR for compound BTL-22. Fig. S54 ^1^H NMR for compound BTL-23. Fig. S55 ^13^C NMR for compound BTL-23. Fig. S56 ^1^H NMR for compound BTL-24. Fig. S57 ^13^C NMR for compound BTL-24.

## Data Availability

All relevant data are available with the article and its Additional file 1, or available the corresponding authors upon reasonable requests.
