# Supplementary material for: Design of a delivery vehicle chitosan-based self-assembling: controlled release, high hydrophobicity, and safe treatment of plant fungal diseases
Source: J Nanobiotechnology. 2024 Mar 19;22:121. doi: 10.1186/s12951-024-02386-8 (PMC10949580; doi:10.1186/s12951-024-02386-8)
Supplement: Supplementary file 1 — Additional file 1. Table S1. Loading content (LC) and encapsulation efficiency (EE)a.Table S2. In vitro activity of BTL-1−BTL-24 at 10 μg/mL against R. solani, P. capsici, B. cinerea, and S.sclerotiorumA. Table S3. Effect of BTL-11 and BTL-11@NSCS NPs on chitinase content of R. solaniA. Table S4. Effect of BTL-11 and BTL-11@NSCS NPs on MDA content of R. solaniA. Table S5. Effect of BTL-11 and BTL-11@NSCS NPs on protein content of R. solaniA. Table S6. Effect of BTL-11 and BTL-11@NSCS NPs on total sugar content of R. solaniA. Table S7. X-ray single crystal data of compound BTL-8. Fig. S1 Morphology of mycelia R. solani treated with BTL-11 and BTL-11@NSCS NPs at 20 μg/mL. A-1, A-2: CK; B-1, B-2: BTL-11; C-1, C-2: BTL-11@NSCS NPs; magnification: 10×10; scale for 10 μm. Fig. S2 Photographs of solutions of NSCS 2 mg/mL in water, BTL-11@NSCS NPs: BTL-11 2 mg/mL in NSCS solution, BTL-11 2 mg/mL in water. Fig. S3 X-ray single crystal structure of compound BTL-8. Fig. S4 1H NMR for compound BTL-1. Fig. S5 13C NMR for compound BTL-1. Fig. S6 1H NMR for compound BTL-2. Fig. S7 13C NMR for compound BTL-2. Fig. S8 19F NMR for compound BTL-2. Fig. S9 1H NMR for compound BTL-3. Fig. S10 13C NMR for compound BTL-3. Fig. S11 1H NMR for compound BTL-4. Fig. S12 13C NMR for compound BTL-4. Fig. S13 1H NMR for compound BTL-5. Fig. S14 13C NMR for compound BTL- Fig. S15 1H NMR for compound BTL-6. Fig. S16 13C NMR for compound BTL-6. Fig. S17 1H NMR for compound BTL-7. Fig. S18 13C NMR for compound BTL-7. Fig. S19 19F NMR for compound BTL-7. Fig. S20 1H NMR for compound BTL-8. Fig. S21 13C NMR for compound BTL-8. Fig. S22 1H NMR for compound BTL-9. Fig. S23 13C NMR for compound BTL-9. Fig. S24 1H NMR for compound BTL-10. Fig. S25 13C NMR for compound BTL-10. Fig. S26 19F NMR for compound BTL-10. Fig. S27 1H NMR for compound BTL-11. Fig. S28 13C NMR for compound BTL-11. Fig. S29 1H NMR for compound BTL-12. Fig. S30 13C NMR for compound BTL-12. Fig. S31 1H NMR for compound BTL-13. Fig. S32 [file 12951_2024_2386_MOESM1_ESM.docx]

**Supplementary Information**

**Design of a delivery vehicle chitosan-based** **self-assembling:** **controlled release, high hydrophobicity, and safe treatment of plant fungal diseases**

Qing Zhou^1^†, Zhi Xia^1,2^†, Yu Zhang^2^, ZhilingSun^1^, Wei Zeng^1^, Nian Zhang^1^, Chunmei Yuan^1^, Chenyu Gong^1^, Yuanxiang Zhou^1^ and Wei Xue ^1^*

^1^ National Key Laboratory of Green Pesticide, Key Laboratory of Green Pesticide and Agricultural Bioengineering, Ministry of Education, Center for R&D of Fine Chemicals of Guizhou University, Guiyang, 550025, China

^2^ College of Chemistry and Chemical Engineering, Guizhou University of Engineering Science, Bijie 551700, China

†Co-first authors

*Corresponding author: Tel: 0086-851-88292090, Fax: 0086-851-88292090, E-mail: wxue@gzu.edu.cn

**Tables and Figs**

**Table S1** Loading content (LC) and encapsulation efficiency (EE).^a^

**Table S2** *In vitro* activity of BTL-1−BTL-24 at 10 *μ*g/mL against *R. solani*, *P. capsici*, *B. cinerea*, and *S. sclerotiorum*.^A^

**Table S3** Effect of BTL-11 and BTL-11@NSCS NPs on chitinase content of *R. solani*.^A^

**Table S4** Effect of BTL-11 and BTL-11@NSCS NPs on MDA content of *R. solani*.^A^

**Table S5** Effect of BTL-11 and BTL-11@NSCS NPs on protein content of *R. solani*.^A^

**Table S6** Effect of BTL-11 and BTL-11@NSCS NPs on total sugar content of *R. solani*.^A^

**Table S7** *X*-ray single crystal data of compound BTL-8.

**Fig. S1** Morphology of mycelia *R. solani* treated with BTL-11 and BTL-11@NSCS NPs at 20 *μ*g/mL. **A-1, A-2:** CK; **B-1, B-2**: BTL-11; **C-1, C-2**: BTL-11@NSCS NPs; magnification: 10×10; scale for 10 *µ*m.

**Fig. S2** Photographs of solutions of NSCS 2 mg/mL in water, BTL-11@NSCS NPs: BTL-11 2 mg/mL in NSCS solution, BTL-11 2 mg/mL in water.

**Fig. S3** *X*-ray single crystal structure of compound BTL-8.

**Fig. S4** ^1^H NMR for compound BTL-1

**Fig. S5** ^13^C NMR for compound BTL-1

**Fig. S6** ^1^H NMR for compound BTL-2

**Fig. S7** ^13^C NMR for compound BTL-2

**Fig. S8** ^19^F NMR for compound BTL-2

**Fig. S9** ^1^H NMR for compound BTL-3

**Fig. S10** ^13^C NMR for compound BTL-3

**Fig. S11** ^1^H NMR for compound BTL-4

**Fig. S12** ^13^C NMR for compound BTL-4

**Fig. S13** ^1^H NMR for compound BTL-5

**Fig. S14** ^13^C NMR for compound BTL-5

**Fig. S15** ^1^H NMR for compound BTL-6

**Fig. S16** ^13^C NMR for compound BTL-6

**Fig. S17** ^1^H NMR for compound BTL-7

**Fig. S18** ^13^C NMR for compound BTL-7

**Fig. S19** ^19^F NMR for compound BTL-7

**Fig. S20** ^1^H NMR for compound BTL-8

**Fig. S21** ^13^C NMR for compound BTL-8

**Fig. S22** ^1^H NMR for compound BTL-9

**Fig. S23** ^13^C NMR for compound BTL-9

**Fig. S24** ^1^H NMR for compound BTL-10

**Fig. S25** ^13^C NMR for compound BTL-10

**Fig. S26** ^19^F NMR for compound BTL-10

**Fig. S27** ^1^H NMR for compound BTL-11

**Fig. S28** ^13^C NMR for compound BTL-11

**Fig. S29** ^1^H NMR for compound BTL-12

**Fig. S30** ^13^C NMR for compound BTL-12

**Fig. S31** ^1^H NMR for compound BTL-13

**Fig. S32** ^13^C NMR for compound BTL-13

**Fig. S33** ^1^H NMR for compound BTL-14

**Fig. S34** ^13^C NMR for compound BTL-14

**Fig. S35** ^1^H NMR for compound BTL-15

**Fig. S36** ^13^C NMR for compound BTL-15

**Fig. S37** ^19^F NMR for compound BTL-15

**Fig. S38** ^1^H NMR for compound BTL-16

**Fig. S39** ^13^C NMR for compound BTL-16

**Fig. S40** ^1^H NMR for compound BTL-17

**Fig. S41** ^13^C NMR for compound BTL-17

**Fig. S42** ^19^F NMR for compound BTL-17

**Fig. S43** ^1^H NMR for compound BTL-18

**Fig. S44** ^13^C NMR for compound BTL-18

**Fig. S45** ^1^H NMR for compound BTL-19

**Fig. S46** ^13^C NMR for compound BTL-19

**Fig. S47** ^1^H NMR for compound BTL-20

**Fig. S48** ^13^C NMR for compound BTL-20

**Fig. S49** ^1^H NMR for compound BTL-21

**Fig. S50** ^13^C NMR for compound BTL-21

**Fig. S51** ^1^H NMR for compound BTL-22

**Fig. S52** ^13^C NMR for compound BTL-22

**Fig. S53** ^19^F NMR for compound BTL-22

**Fig. S54** ^1^H NMR for compound BTL-23

**Fig. S55** ^13^C NMR for compound BTL-23

**Fig. S56** ^1^H NMR for compound BTL-24

**Fig. S57** ^13^C NMR for compound BTL-24

**Tables and Figs**

**Table S1** Loading content (LC) and encapsulation efficiency (EE).^A^

| Regression equation | NSCS:BTL-11 | LC (%) | EE (%) |
| --- | --- | --- | --- |
| Y = 0.0318+0.0898X  (R^2^=0.9980) | 10:1 | 8.1±0.6 | 81.5±2.3 |
|  | 5:1 | 16.1±0.3 | 80.6±0.1 |
|  | 2:1 | 35.5±0.2 | 71.0±0.4 |

^A^ The experiments were repeated 3 times.

**Table S2** *In vitro* activity of BTL-1−BTL-24 at 10 *μ*g/mL against *R. solani*, *P. capsici*, *B. cinerea*, and *S. sclerotiorum*.^A^

| Compounds | Inhibition rate (%)  *R.solani* (%)  *S. Sclerotiorum* (%)  *B. Cinerea* (%) | | | | |
| --- | --- | --- | --- | --- | --- |
|  | R | *R. solani* ^B^ | *P. capsici* | *B. cinerea* | *S. sclerotiorum* |
| BTL-1 | H | 77.2±1.2b | 69.4±1.2d | 55.4±1.1g | 75.2±1.3c |
| BTL-2 | 4-F | 69.5±2.4m | 50.5±1.3p | -^C^ | 73.2±1.3e |
| BTL-3 | 4-Cl | 70.5±1.2k | 63.0±1.2h | -^C^ | 57.6±1.1k |
| BTL-4 | 4-Br | 75.2±2.0d | 69.4±1.2d | 64.7±1.1c | 45.2±1.1s |
| BTL-5 | 4-CH_3_ | 65.0±1.2q | 65.0±1.3g | -^C^ | 49.3±1.1q |
| BTL-6 | 4-OCH_3_ | 69.2±1.2n | 45.7±1.3r | -^C^ | 52.8±1.6n |
| BTL-7 | 5-F | 68.0±1.2o | 46.1±1.2q | 60.1±1.2e | 45.2±1.1s |
| BTL-8 | 5-Cl | 62.0±1.4s | 62.6±1.3i | 71.0±1.6b | 41.7±1.6t |
| BTL-9 | 5-Br | 56.7±1.2v | 43.3±1.3t | 78.1±1.2a | 65.9±1.1g |
| BTL-10 | 6-F | 67.8±1.7p | 68.2±1.8e | -^C^ | -^C^ |
| BTL-11 | 6-Cl | 76.1±1.1c | 67.7±1.3f | -^C^ | 62.4±1.6j |
| BTL-11@NSCS NPs | - | 80.2±1.1a | 74.4±1.0b | 60.1±1.2e | 72.8±2.1f |
| BTL-12 | 6-Br | 74.1±1.2e | 57.7±1.3n | 53.6±1.2h | 53.4±1.1m |
| BTL-13 | 6-NO_2_ | 63.9±2.2r | 61.4±1.2j | -^C^ | -^C^ |
| BTL-14 | 6-CH_3_ | 59.4±1.2u | 56.5±1.3o | 43.2±1.1j | 62.8±1.2i |
| BTL-15 | 6-CF_3_ | 75.0±1.1d | 60.2±1.3k | -^C^ | 51.0±1.1p |
| BTL-16 | 6-OCH_3_ | 71.8±1.2h | 43.3±1.3t | -^C^ | 57.2±1.3l |
| BTL-17 | 6-OCF_3_ | 72.7±1.2g | 45.7±1.3r | 61.7±3.2d | 74.5±1.1d |
| BTL-18 | 6-COCH_2_CH_3_ | 71.1±1.7i | 45.3±1.2s | -^C^ | 76.6±1.1b |
| BTL-19 | 6-COOCH_3_ | 60.5±1.2t | 83.9±1.2a | -^C^ | 53.4±1.2m |
| BTL-20 | 6-OH | -^C^ | -^C^ | -^C^ | -^C^ |
| BTL-21 | 6-SOOCH_3_ | 73.3±2.6f | 71.4±2.8c | 46.0±3.5i | 41.4±2.8u |
| BTL-22 | 4,6-di-F | 69.9±1.2l | 58.1±1.2m | -^C^ | 51.7±1.2o |
| BTL-23 | 4,6-di-Cl | 70.8±1.1j | 58.6±9.3l | 56.5±1.6f | 80.7±1.1a |
| BTL-24 | 4,6-di-CH_3_ | 53.4±1.2w | 23.9±1.3u | -^C^ | 63.8±2.8h |
| fluopyram ^D^ | - | -^C^ | -^C^ | -^C^ | -^C^ |
| azoxystrobin ^D^ | - | 51.4±1.0x | -^C^ | -^C^ | 48.6±1.1r |

^A^ The experiments were repeated 3 times; P<0.05, Values are the mean ± standard deviation (SD).

^B^ *R. solani: Rhizoctonia solani*; *P. capsici: Phytophthora capsici; B. cinerea: Botrytis cinerea, and S. sclerotiorum: Sclerotinia sclerotiorum.*

*^C^* “-”<30%.

^D^ azoxystrobin and fluopyram were used as control agents.

**Table S3** Effect of BTL-11 and BTL-11@NSCS NPs on chitinase content of *R. solani*.^A^

| Treatment | Content of chitinase (*U*/g) | | | | |
| --- | --- | --- | --- | --- | --- |
|  | CK (0 *μ*g/mL) | 100 *μ*g/mL | 50 *μ*g/mL | 25 *μ*g/mL | 12.5 *μ*g/mL |
| BTL-11 | 623.2 ± 3.6a | 145.0 ± 3.3e | 205.3 ± 3.5d | 421.3 ± 3.5c | 480.2 ±3.4b |
| BTL-11@NSCS NPs | 623.2 ± 3.6a | 125.4 ± 3.4e | 185.1 ± 3.1d | 421.7 ± 3.2c | 472.6 ±3.7b |

^A^ The experiments were repeated 3 times, P<0.05, values are the mean ± standard deviation (SD).

**Table S4** Effect of BTL-11 and BTL-11@NSCS NPs on MDA content of *R. solani*.^A^

| Treatment | Content of MDA (nmol/g) | | | | |
| --- | --- | --- | --- | --- | --- |
|  | CK (0 *μ*g/mL) | 50 *μ*g/mL | 25 *μ*g/mL | 12.5 *μ*g/mL | 6.25 *μ*g/mL |
| BTL-11 | 22.4 ± 0.4e | 51.8 ± 0.3a | 43.8 ± 0.2b | 35.1 ± 0.3c | 28.6 ±0.5d |
| BTL-11@NSCS NPs | 22.4 ± 0.4e | 57.7 ± 0.3a | 44.5 ± 0.4b | 34.3 ± 0.5c | 32.1 ±0.6d |

^A^ The experiments were repeated 3 times, P<0.05, values are the mean ± standard deviation (SD).

**Table S5** Effect of BTL-11 and BTL-11@NSCS NPs on protein content of *R. solani*.^A^

| Treatment | Content of protein (*μ*g/mL) | | | | |
| --- | --- | --- | --- | --- | --- |
|  | CK (0 *μ*g/mL) | 100 *μ*g/mL | 50 *μ*g/mL | 25 *μ*g/mL | 12.5 *μ*g/mL |
| BTL-11 | 35.7 ± 1.4e | 74.8 ± 1.3a | 67.4 ± 1.2b | 53.4 ± 1.4c | 37.6 ±1.4d |
| BTL-11@NSCS NPs | 35.7 ± 1.4e | 75.5 ± 1.6a | 66.2 ± 1.7b | 54.1 ± 1.5c | 39.6 ±1.4d |

^A^ The experiments were repeated 3 times, P<0.05, values are the mean ± standard deviation (SD).

**Table S6** Effect of BTL-11 and BTL-11@NSCS NPs on total sugar content of *R. solani*.^A^

| Treatment | Content of total sugar (mg/g) | | | | |
| --- | --- | --- | --- | --- | --- |
|  | CK (0 *μ*g/mL) | 100 *μ*g/mL | 50 *μ*g/mL | 25 *μ*g/mL | 12.5 *μ*g/mL |
| BTL-11 | 118.7 ± 1.8a | 25.1 ± 0.7e | 33.8 ± 1.1d | 61.6 ± 1.4c | 85.5 ±1.8b |
| BTL-11@NSCS NPs | 118.7 ± 1.8a | 22.3 ± 0.7e | 34.3 ± 0.6d | 64.4 ± 0.6c | 81.2 ±1.4b |

^A^ The experiments were repeated 3 times, P<0.05, values are the mean ± standard deviation (SD).

**Table S7** *X*-ray single crystal data of compound BTL-8.

| Compound | | BTL-8 |
| --- | --- | --- |
| Sum formula | | C_9_H_6_Cl_2_N_2_OS |
| CCDC numbers | | 2306285 |
| Formula weight | | 261.12 |
| Crystal system | | Monoclinic |
| Space group | | P 1 21/c 1 |
| Temperature / K | | 273.15 |
| Wavelength | | 1.54178 |
| Dx [g cm^-3^] | | 1.137 |
| Mu [mm^-1^] | | 4.957 |
| a [Å] | | 12.4825(8) |
| b [Å] | | 17.9489(11) |
| c [Å] | | 6.8380(4) |
| *α* [°] | | 90 |
| *β* [°] | | 95.032(3) |
| *γ* [°] | | 90 |
| *V* [Å^3^] | | 1526.13(16) |
| *Z* | | 4 |
| F [000] | | 528.0 |
| h, k, l | | 13, 19, 7 |
| Reflns number | | 8255 |
| Goodness of fit on F^2^ | | 1.090 |
| Theta min to max [°] | | 3.554 to 58.919 |
| Radiation type | | CuK / a |
| Final R indices [*I* > 2*σ* (*I*)] | | R_1_ = 0.0800, wR_2_ = 0.2572 |
| R indices [all data] | R_1_ = 0.0944, wR_2_ = 0.2781 |  |

**Fig. S1** Morphology of mycelia *R. solani* treated with BTL-11 and BTL-11@NSCS NPs at 20 *μ*g/mL. **A-1, A-2**: CK; **B-1, B-2**: BTL-11; **C-1, C-2**: BTL-11@NSCS NPs; magnification: 10×10; scale for 10 *µ*m.

**Fig. S2** Photographs of solutions of NSCS 2 mg/mL in water, BTL-11@NSCS NPs: BTL-11 2 mg/mL in NSCS solution, BTL-11 2 mg/mL in water.

**Fig. S3** *X*-ray single crystal structure of compound BTL-8.

**Characterization of target compounds** **BTL-1−BTL-24.**

**BTL-1**

***N*-(benzo[*d*]thiazol-2-yl)-2-chloroacetamide**: m.p.165-166℃; white solid; yield: 82%.

**^1^H NMR (400 MHz, DMSO-*d*_6_)** δ 12.47 (s, 1H, -NH), 8.01 (t, *J* = 8.1 Hz, 1H, Ph), 7.78 (t, *J* = 8.8 Hz, 1H, Ph), 7.51 – 7.44 (m, 1H, Ph), 7.38 – 7.32 (m, 1H, Ph), 4.51 (s, 2H, -CH_2_).

**^13^C NMR NMR (100 MHz, DMSO-*d*_6_)** δ 164.30 (s), 155.91 (s), 146.68 (s), 129.76 (s), 124.58 (s), 122.12 (s), 120.15 (s), 119.01 (s), 40.89 (s).

**BTL-2**

**2-chloro-*N*-(4-fluorobenzo[*d*]thiazol-2-yl)acetamide**: m.p.196-197℃; white solid; yield: 83%.

**^1^H NMR (400 MHz, DMSO-*d*_6_)** δ 13.12 (s, 1H, -NH), 7.90 – 7.83 (m, 1H, Ph), 7.68 (d, *J* = 7.8 Hz, 1H, Ph), 7.34 (dt, *J* = 5.6, 2.9 Hz, 1H, Ph), 4.54 (d, *J* = 1.5 Hz, 2H, -CH_2_).

**^13^C NMR NMR (100 MHz, DMSO-*d*_6_)** δ 169.21 (s), 166.62 (s), 158.64 (s), 125.13 (d, *J* = 6.8 Hz), 124.02 (d, *J* = 6.3 Hz), 118.86 (d, *J* = 3.2 Hz), 113.40 (d, *J* = 17.1 Hz), 112.48 (d, *J* = 17.6 Hz), 42.93 (s).

**^19^F NMR (376 MHz, DMSO-*d*_6_)** δ -127.32 (s).

**BTL-3**

**2-chloro-*N*-(4-chlorobenzo[*d*]thiazol-2-yl)acetamide**: m.p.175-177℃; white solid; yield: 88%.

**^1^H NMR (400 MHz, DMSO-*d*_6_)** δ 13.19 (s, 1H, -NH), 8.03 – 7.95 (m, 1H, Ph), 7.41 (dd, *J* = 9.9, 5.8 Hz, 1H, Ph), 7.16 (td, *J* = 7.7, 4.6 Hz, 1H, Ph), 4.49 (s, 2H, -CH_2_).

**^13^C NMR NMR (126 MHz, DMSO-*d*_6_)** δ 166.68 (s), 158.95 (s), 147.84 (s), 133.68 (s), 128.41 (s), 127.15 (s), 122.47 (s), 122.07 (s), 43.03 (s).

**BTL-4**

***N*-(4-bromobenzo[*d*]thiazol-2-yl)-2-chloroacetamide**: m.p.183-184℃; white solid; yield: 78%.

**^1^H NMR (400 MHz, DMSO-*d*_6_)** δ 13.20 (s, 1H, -NH), 8.05 (d, *J* = 7.9 Hz, 1H, Ph), 7.82 (d, *J* = 7.8 Hz, 1H, Ph), 7.71 (d, *J* = 7.8 Hz, 1H, Ph), 4.51 (s, 2H, -CH_2_).

**^13^C NMR NMR (100 MHz, DMSO-*d*_6_)** δ 168.94 (s), 166.69 (s), 158.82 (s), 132.97 (s), 129.91 (s), 125.54 (s), 122.04 (s), 114.13 (s), 42.92 (s).

**BTL-5**

**2-chloro-*N*-(4-methylbenzo[*d*]thiazol-2-yl)acetamide**: m.p.201-202℃; white solid; yield: 87%.

**^1^H NMR (400 MHz, DMSO-*d*_6_)** δ 9.73 (s, 1H, -NH), 7.76 (d, *J* = 7.6 Hz, 1H, Ph), 7.29 (d, *J* = 7.3 Hz, 1H, Ph), 7.24 (d, *J* = 7.7 Hz, 1H, Ph), 4.49 (s, 2H, -CH_2_), 2.48 (s, 3H, -CH_3_).

**^13^C NMR NMR (100 MHz, DMSO-*d*_6_)** δ 170.02 (s), 166.32 (s), 157.10 (s), 137.45 (s), 129.06 (s), 124.78 (s), 124.21 (s), 121.04 (s), 42.95 (s), 17.94 (s).

**BTL-6**

**2-chloro-*N*-(4-methoxybenzo[*d*]thiazol-2-yl)acetamide**: m.p.197-198℃; white solid; yield: 81%.

**^1^H NMR (400 MHz, DMSO-*d*_6_)** δ 9.85 (s, 1H, -NH), 7.56 (dd, *J* = 8.0, 0.6 Hz, 1H, Ph), 7.31 – 7.26 (m, 1H, Ph), 7.01 (t, *J* = 6.7 Hz, 1H, Ph), 4.50 (s, 2H, -CH_2_), 3.93 (s, 3H, -OCH_3_).

**^13^C NMR NMR (100 MHz, DMSO-*d*_6_)** δ 166.23 (s), 156.36 (s), 152.35 (s), 138.72 (s), 133.30 (s), 125.29 (s), 114.03 (s), 108.15 (s), 56.23 (s), 42.94 (s).

**BTL-7**

**2-chloro-*N*-(5-fluorobenzo[*d*]thiazol-2-yl)acetamide**: m.p.185-186℃; white solid; yield: 80%.

**^1^H NMR (400 MHz, DMSO-*d*_6_)** δ 10.21 (s, 1H, -NH), 7.97 – 7.95 (m, 1H, Ph), 7.42 (dd, *J* = 9.1, 2.2 Hz, 1H, Ph), 7.21 (dd, *J* = 6.4, 2.6 Hz, 1H, Ph), 4.54 (s, 2H, -CH_2_).

**^13^C NMR NMR (100 MHz, DMSO-*d*_6_)** δ 170.66 (s), 166.58 (s), 160.54 (d, *J* = 252.6 Hz), 140.67 (d, *J* = 12.6 Hz), 125.06 (d, *J* = 9.8 Hz), 112.04 (d, *J* = 3.2 Hz), 107.40 (d, *J* = 17.2 Hz), 102.29 (d, *J* = 17.6 Hz), 42.99 (s).

**^19^F NMR (376 MHz, DMSO-*d*_6_)** δ -114.00 (s).

**BTL-8**

**2-chloro-*N*-(5-chlorobenzo[*d*]thiazol-2-yl)acetamide**: m.p.196-197℃; white solid; yield: 84%.

**^1^H NMR (400 MHz, DMSO-*d*_6_)** δ 10.17 (s, 1H, -NH), 7.85 (d, *J* = 2.0 Hz, 1H, Ph), 7.61 (d, *J* = 2.0 Hz, 1H, Ph), 7.37 (d, *J* = 2.0 Hz, 1H, Ph), 4.53 (s, 2H, -CH_2_).

**^13^C NMR NMR (100 MHz, DMSO-*d*_6_)** δ 170.08 (s), 166.69 (s), 159.94 (s), 124.97 (s), 124.36 (s), 123.88 (s), 120.65 (s), 114.55 (s), 42.99 (s).

**BTL-9**

***N*-(5-bromobenzo[*d*]thiazol-2-yl)-2-chloroacetamide**: m.p.210-211℃; white solid; yield: 71%.

**^1^H NMR (400 MHz, DMSO-*d*_6_)** δ 12.88 (s, 1H, -NH), 8.29 (d, *J* = 2.0 Hz, 1H, Ph), 7.72 (d, *J* = 8.6 Hz, 1H, Ph), 7.60 (dd, *J* = 8.6, 2.0 Hz, 1H, Ph), 4.50 (s, 2H, -CH_2_).

**^13^C NMR NMR (100 MHz, DMSO-*d*_6_)** δ 166.64 (s), 158.86 (s), 148.07 (s), 134.11 (s), 129.75 (s), 124.86 (s), 122.80 (s), 116.24 (s), 43.00 (s).

**BTL-10**

**2-chloro-*N*-(6-fluorobenzo[*d*]thiazol-2-yl)acetamide**: m.p.206-207℃; white solid; yield: 70%.

**^1^H NMR (400 MHz, DMSO-*d*_6_)** δ 10.26 (s, 1H, -NH), 7.86 (dd, *J* = 8.5, 2.5 Hz, 1H, Ph), 7.56 (dd, *J* = 8.9, 4.5 Hz, 1H, Ph), 7.30 (s, 1H, Ph), 4.51 (s, 2H, -CH_2_).

**^13^C NMR NMR (100 MHz, DMSO-*d*_6_)** δ 169.59 (s), 166.56 (s), 157.90 (d, *J* = 250.6 Hz), 133.16 (d, *J* = 12.8 Hz), 122.31 (d, *J* = 8.7 Hz), 115.47 (d, *J* = 3.6 Hz), 110.79 (d, *J* = 8.6 Hz), 108.73 (d, *J* = 11.2 Hz), 42.95 (s).

**^19^F NMR (376 MHz, DMSO-*d*_6_)** δ -117.98 (s).

**BTL-11**

**2-chloro-*N*-(6-chlorobenzo[*d*]thiazol-2-yl)acetamide**: m.p.223-224℃; white solid; yield: 93%.

**^1^H NMR (400 MHz, DMSO-*d*_6_)** δ 12.78 (s, 1H, -NH), 8.09 (d, *J* = 2.2 Hz, 1H, Ph), 7.71 (d, *J* = 8.6 Hz, 1H, Ph), 7.42 (dd, *J* = 8.6, 2.2 Hz, 1H, Ph), 4.43 (s, 2H, -CH_2_).

**^13^C NMR NMR (100 MHz, DMSO-*d*_6_)** δ 166.68 (s), 158.95 (s), 147.84 (s), 133.68 (s), 128.41 (s), 127.15 (s), 122.47 (s), 122.07 (s), 43.03 (s).

**BTL-12**

***N*-(6-bromobenzo[*d*]thiazol-2-yl)-2-chloroacetamide**: m.p.188-189℃; white solid; yield: 89%.

**^1^H NMR (400 MHz, DMSO-*d*_6_)** δ 10.34 (s, 1H, -NH), 8.20 (d, *J* = 2.0 Hz, 1H, Ph), 7.71 (t, *J* = 5.7 Hz, 1H, Ph), 7.52 (d, *J* = 8.6 Hz, 1H, Ph), 4.55 (s, 2H, -CH_2_).

**^13^C NMR NMR (100 MHz, DMSO-*d*_6_)**δ 169.51 (s), 166.70 (s), 158.80 (s), 130.71 (s), 126.74 (s), 124.83 (s), 122.79 (s), 116.20 (s), 42.98 (s).

**BTL-13**

**2-chloro-*N*-(6-nitrobenzo[*d*]thiazol-2-yl)acetamide**: m.p.207-208℃; white solid; yield: 61%.

**^1^H NMR (400 MHz, DMSO-*d*_6_)** δ 9.92 (s, 1H, -NH), 8.86 (d, *J* = 2.4 Hz, 1H, Ph), 7.92 (t, *J* = 6.2 Hz, 1H, Ph), 7.63 (d, *J* = 8.9 Hz, 1H, Ph), 4.60 (s, 2H, -CH_2_).

**^13^C NMR NMR (100 MHz, DMSO-*d*_6_)** δ 171.71 (s), 167.20 (s), 163.51 (s), 132.64 (s), 128.09 (s), 123.39 (s), 119.51 (s), 115.55 (s), 43.04 (s).

**BTL-14**

**2-chloro-*N*-(6-methylbenzo[*d*]thiazol-2-yl)acetamide**: m.p.205-206℃; white solid; yield: 79%.

**^1^H NMR (400 MHz, DMSO-*d*_6_)** δ 10.17 (s, 1H, -NH), 7.78 (d, *J* = 6.8 Hz, 1H, Ph), 7.65 (t, *J* = 8.9 Hz, 1H, Ph), 7.27 (d, *J* = 8.3 Hz, 1H, Ph), 4.51 (s, 2H, -CH_2_), 2.42 (s, 3H, -CH_3_).

**^13^C NMR NMR (100 MHz, DMSO-*d*_6_)** δ 169.13 (s), 166.35 (s), 157.14 (s), 133.77 (s), 128.06 (s), 121.86 (s), 120.79 (s), 114.15 (s), 43.00 (s), 21.47 (s).

**BTL-15**

**2-chloro-*N*-(6-(trifluoromethyl)benzo[*d*]thiazol-2-yl)acetamide**: m.p.197-198℃; white solid; yield: 87%.

**^1^H NMR (400 MHz, DMSO-*d*_6_)** δ 10.22 (s, 1H, -NH), 8.40 (s, 1H, Ph), 7.95 (t, *J* = 6.2 Hz, 1H, Ph), 7.72 (t, *J* = 7.5 Hz, 1H, Ph), 4.57 (s, 2H, -CH_2_).

**^13^C NMR NMR (100 MHz, DMSO-*d*_6_)** δ 170.53 (s), 166.97 (s), 161.26 (s), 132.49 (s), 126.37 (s), 124.85 (s), 123.51 (d, *J* = 3.4 Hz), 121.63 (s), 115.36 (s), 43.02 (s).

**^19^F NMR (376 MHz, DMSO-*d*_6_)** δ -59.96 (s).

**BTL-16**

**2-chloro-*N*-(6-methoxybenzo[*d*]thiazol-2-yl)acetamide**: m.p.201-201℃; white solid; yield: 83%.

**^1^H NMR (400 MHz, DMSO-*d*_6_)** δ 10.13 (s, 1H, -NH), 7.68 (d, *J* = 8.8 Hz, 1H, Ph), 7.50 (d, *J* = 8.9 Hz, 1H, Ph), 7.08 – 7.05 (m, 1H, Ph), 4.52 (s, 2H, -CH_2_), 3.82 (s, 3H, -OCH_3_).

**^13^C NMR NMR (100 MHz, DMSO-*d*_6_)** δ 166.18 (s), 156.72 (s), 142.91 (s), 133.24 (s), 121.78 (s), 115.56 (s), 107.95 (s), 105.18 (s), 56.08 (s), 42.96 (s).

**BTL-17**

**2-chloro-*N*-(6-(trifluoromethoxy)benzo[*d*]thiazol-2-yl)acetamide**: m.p.188-189℃; white solid; yield: 82%.

**^1^H NMR (400 MHz, DMSO-*d*_6_)** δ 10.29 (s, 1H, -NH), 8.12 (d, *J* = 22.4 Hz, 1H, Ph), 7.84 (ddd, *J* = 12.8, 7.7, 5.1 Hz, 1H, Ph), 7.43 (dd, *J* = 10.7, 5.4 Hz, 1H, Ph), 4.53 (s, 2H, -CH_2_).

**^13^C NMR NMR (100 MHz, DMSO-*d*_6_)** δ 170.08 (s), 166.76 (s), 159.54 (s), 147.94 (s), 144.64 (s), 133.15 (s), 122.23 (s), 120.47 (s), 115.60 (s), 42.98 (s).

**^19^F NMR (376 MHz, DMSO-*d*_6_)** δ -57.04 (s).

**BTL-18**

**2-chloro-*N*-(6-propionylbenzo[*d*]thiazol-2-yl)acetamide**: m.p.156-157℃; white solid; yield: 69%.

**^1^H NMR (400 MHz, DMSO-*d*_6_)** δ 10.06 (s, 1H, -NH), 7.64 (d, *J* = 8.8 Hz, 1H, Ph), 7.55 (dd, *J* = 8.4, 2.2 Hz, 1H, Ph), 7.01 (dt, *J* = 7.6, 3.8 Hz, 1H, Ph), 4.48 (s, 2H, -CH_2_), 4.04 (q, *J* = 7.0 Hz, 2H, -CH_2_), 1.35 – 1.30 (m, 3H, -CH_3_).

**^13^C NMR NMR (100 MHz, DMSO-*d*_6_)** δ 166.15 (s), 155.96 (s), 155.87 (s), 142.82 (s), 133.22 (s), 121.78 (s), 115.88 (s), 105.79 (s), 64.04 (s), 42.96 (s), 15.15 (s).

**BTL-19**

**Methyl-2-(2-chloroacetamido)benzo[*d*]thiazole-6-carboxylate**: m.p.178-179℃; white solid; yield: 86%.

**^1^H NMR (400 MHz, DMSO-*d*_6_)** δ 10.33 (s, 1H, -NH), 8.66 (t, *J* = 4.6 Hz, 1H, Ph), 8.01 (ddd, *J* = 8.5, 4.8, 1.7 Hz, 2H, Ph), 7.64 (d, *J* = 8.5 Hz, 1H, Ph), 4.57 (s, 2H, -CH_2_), 3.88 (s, 3H, -OCH_3_).

**^13^C NMR NMR (100 MHz, DMSO-*d*_6_)** δ 170.67 (s), 165.87 (s), 161.46 (s), 152.44 (s), 132.21 (s), 129.02 (s), 125.11 (s), 120.97 (s), 114.62 (s), 52.79 (s), 43.03 (s).

**BTL-20**

**2-chloro-*N*-(6-hydroxybenzo[*d*]thiazol-2-yl)acetamide**: m.p.146-147℃; white solid; yield: 65%.

**^1^H NMR (400 MHz, DMSO-*d*_6_)** δ 9.91 (s, 2H, -NH, -OH), 7.38 (d, *J* = 4.2 Hz, 1H, Ph), 7.34 (s, 1H, Ph), 6.93 (d, *J* = 2.3 Hz, 1H, Ph), 4.87 (s, 2H, -CH_2_).

**^13^C NMR NMR (100 MHz, DMSO-*d*_6_)** δ 168.38 (s), 155.14 (s), 130.85 (s), 125.10 (s), 115.98 (s), 115.12 (s), 109.48 (s), 42.99 (s).

**BTL-21**

**2-chloro-*N*-(6-(methylsulfonyl)benzo[*d*]thiazol-2-yl)acetamide**: m.p.173-174℃; white solid; yield: 78%.

**^1^H NMR (400 MHz, DMSO-*d*_6_)** δ 9.40 (s, 1H, -NH), 8.42 (s, 1H, Ph), 7.96 (s, 1H, Ph), 7.65 – 7.61 (m, 1H, Ph), 4.54 (s, 2H, -CH_2_), 3.25 (s, 3H, -CH_3_).

**^13^C NMR NMR (100 MHz, DMSO-*d*_6_)** δ 170.86 (s), 167.06 (s), 162.11 (s), 136.07 (s), 132.47 (s), 125.44 (s), 122.72 (s), 121.57 (s), 44.50 (s), 43.04 (s).

**BTL-22**

**2-chloro-*N*-(4,6-difluorobenzo[*d*]thiazol-2-yl)acetamide**: m.p:182-183℃; white solid; yield: 76%.

**^1^H NMR (400 MHz, DMSO-*d*_6_)** δ 13.12 (s, 1H, -NH), 7.60 (d, *J* = 6.0 Hz, 1H, Ph), 7.28 – 7.24 (m, 1H, Ph), 4.51 (s, 2H, -CH_2_).

**^13^C NMR NMR (100 MHz, DMSO-*d*_6_)** δ 166.70 (s), 158.42 (s), 135.20 (s), 134.22 (s), 105.37 (d, *J* = 36.3 Hz), 104.92 (s), 102.69 (d, *J* = 28.1 Hz), 42.86 (s).

**^19^F NMR (376 MHz, DMSO-*d*_6_)** δ -114.76 (s), -123.65 (s).

**BTL-23**

**2-chloro-*N*-(4,6-dichlorobenzo[*d*]thiazol-2-yl)acetamide**: m.p.211-212℃; white solid; yield: 81%.

**^1^H NMR (400 MHz, DMSO-*d*_6_)** δ 13.30 (s, 1H, -NH), 8.17 (d, *J* = 2.0 Hz, 1H, Ph), 7.46 (d, *J* = 2.0 Hz, 1H, Ph), 4.52 (s, 2H, -CH_2_).

**^13^C NMR NMR (100 MHz, DMSO-*d*_6_)** δ 168.98 (s), 166.85 (s), 159.85 (s), 134.58 (s), 132.40 (s), 128.28 (s), 125.99 (s), 120.64 (s), 42.89 (s).

**BTL-24**

**2-chloro-*N*-(4,6-dimethylbenzo[*d*]thiazol-2-yl)acetamide**: m.p.215-216℃; white solid; yield: 84%.

**^1^H NMR (400 MHz, DMSO-*d*_6_)** δ 10.03 (s, 1H, -NH), 7.67 (d, *J* = 32.3 Hz, 1H, Ph), 7.59 (d, *J* = 32.5 Hz, 1H, Ph), 4.47 (s, 2H, -CH_2_), 2.29 (d, *J* = 10.9 Hz, 3H, -CH_3_), 2.26 (d, *J* = 9.6 Hz, 3H, -CH_3_).

**^13^C NMR NMR (100 MHz, DMSO-*d*_6_)** δ 169.22 (s), 166.22 (s), 147.34 (s), 135.45 (s), 129.23 (s), 121.99 (s), 121.47 (s), 114.92 (s), 42.99 (s), 20.06 (s), 19.86 (s).

**
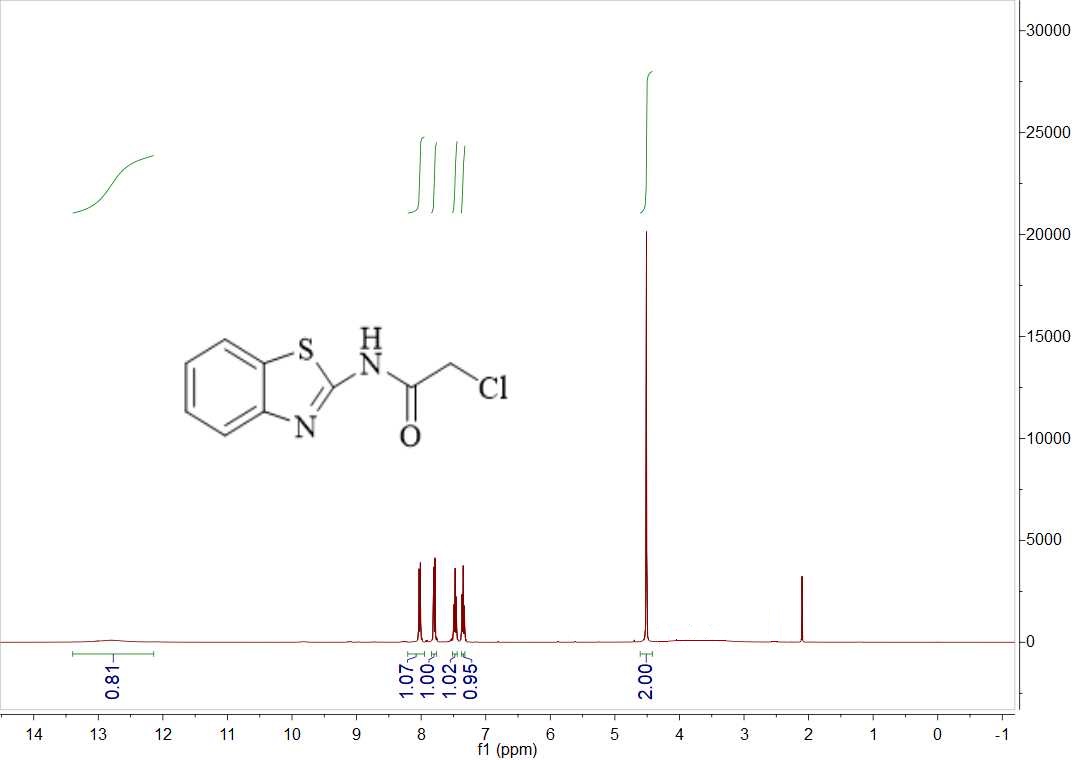
**

**Fig. S4** ^1^H NMR for compound **BTL-1**

**
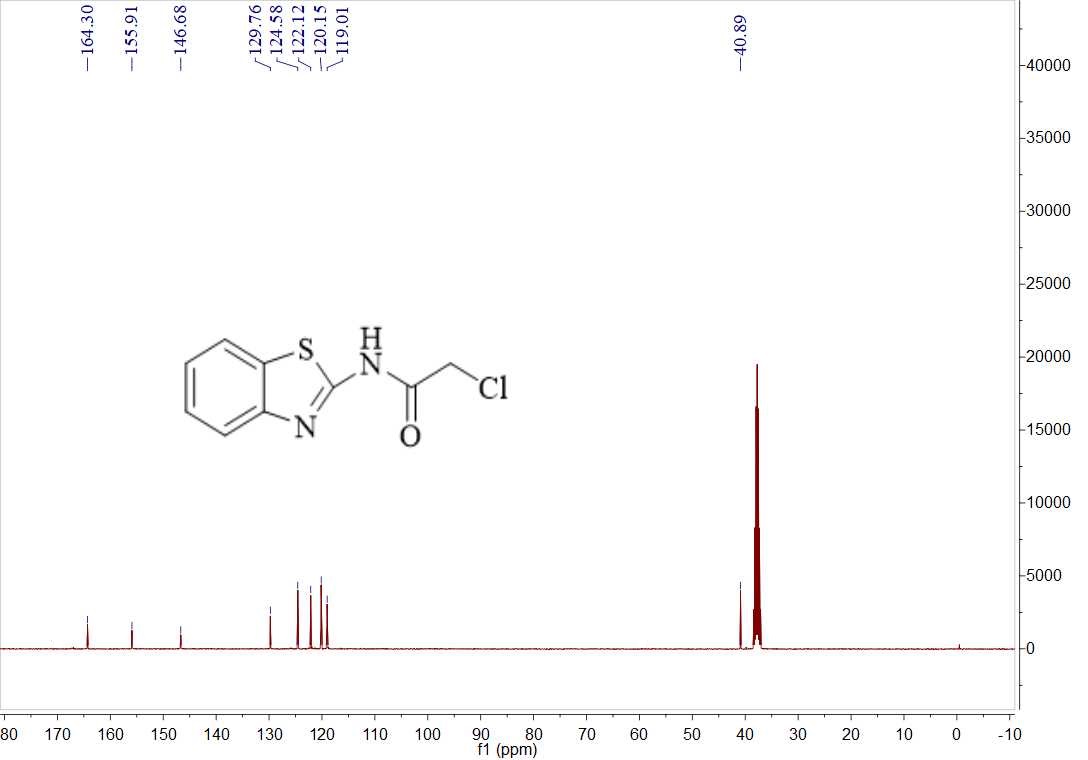
**

**Fig. S5** ^13^C NMR for compound **BTL-1**

**
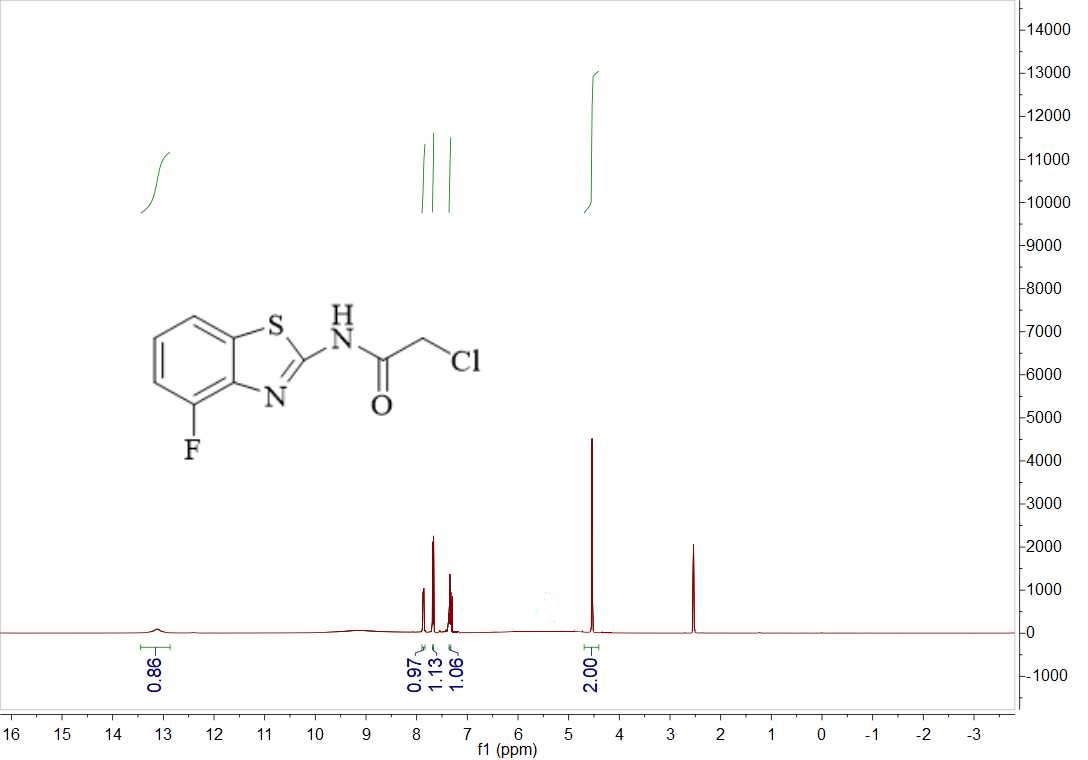
**

**Fig. S6** ^1^H NMR for compound **BTL-2**

**
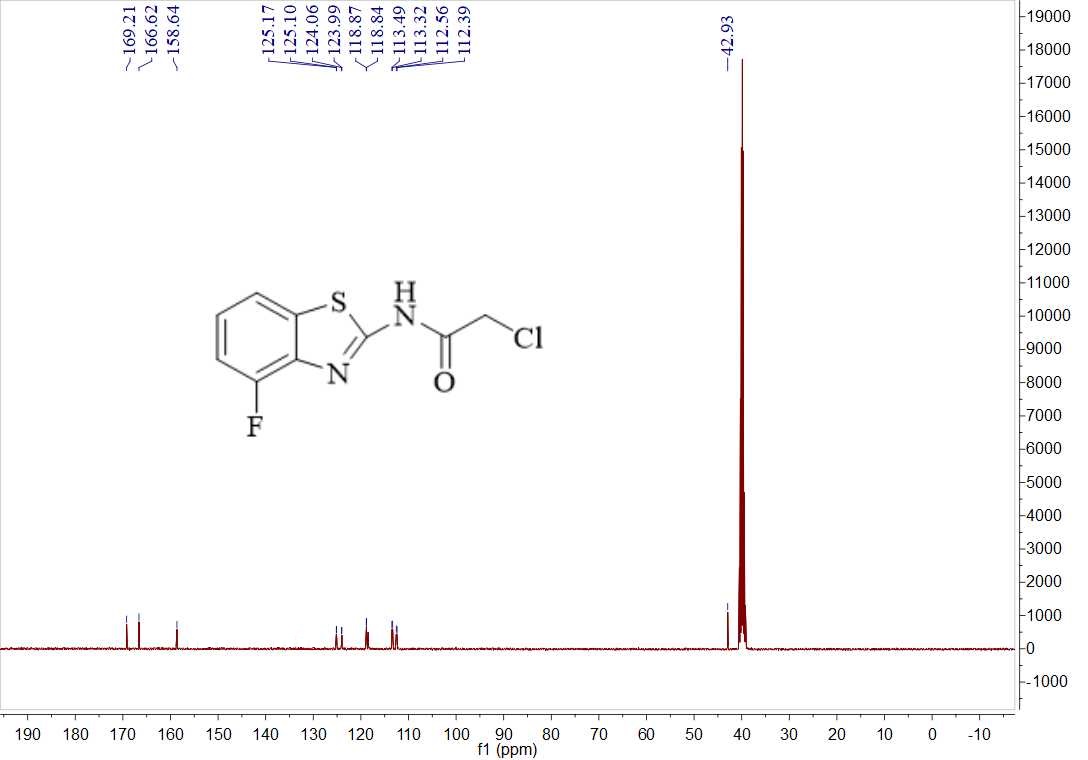
**

**Fig. S7** ^13^C NMR for compound **BTL-2**

**
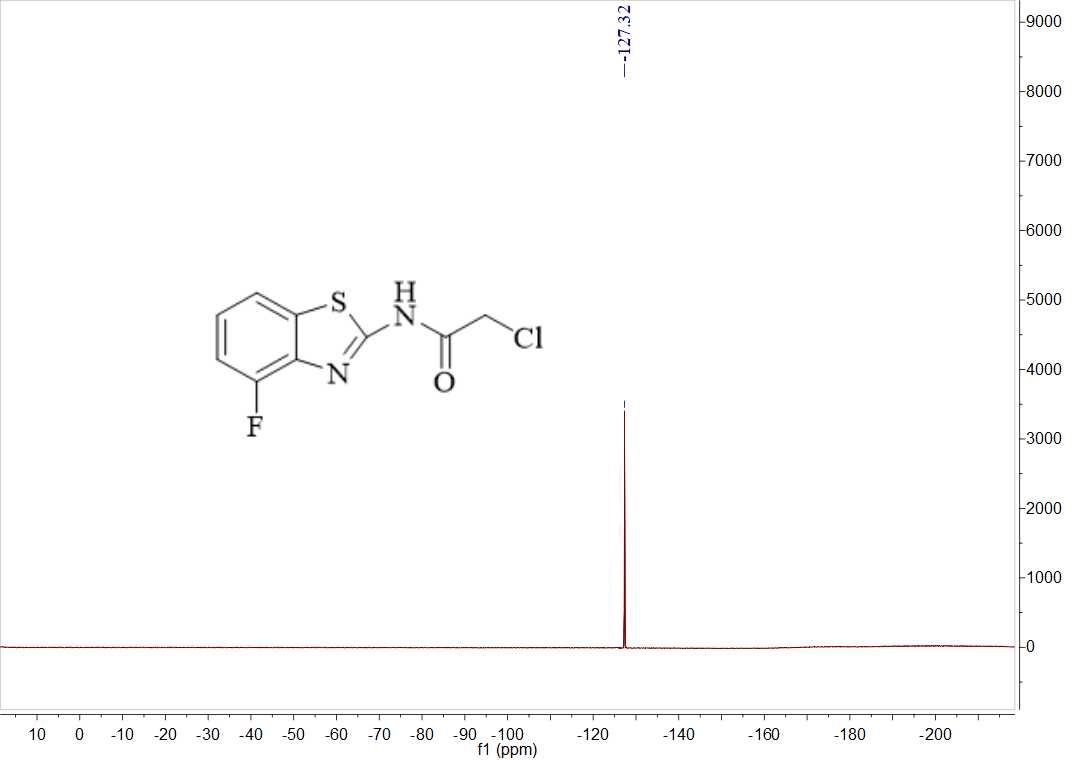
**

**Fig. S8** ^19^F NMR for compound **BTL-2**

**
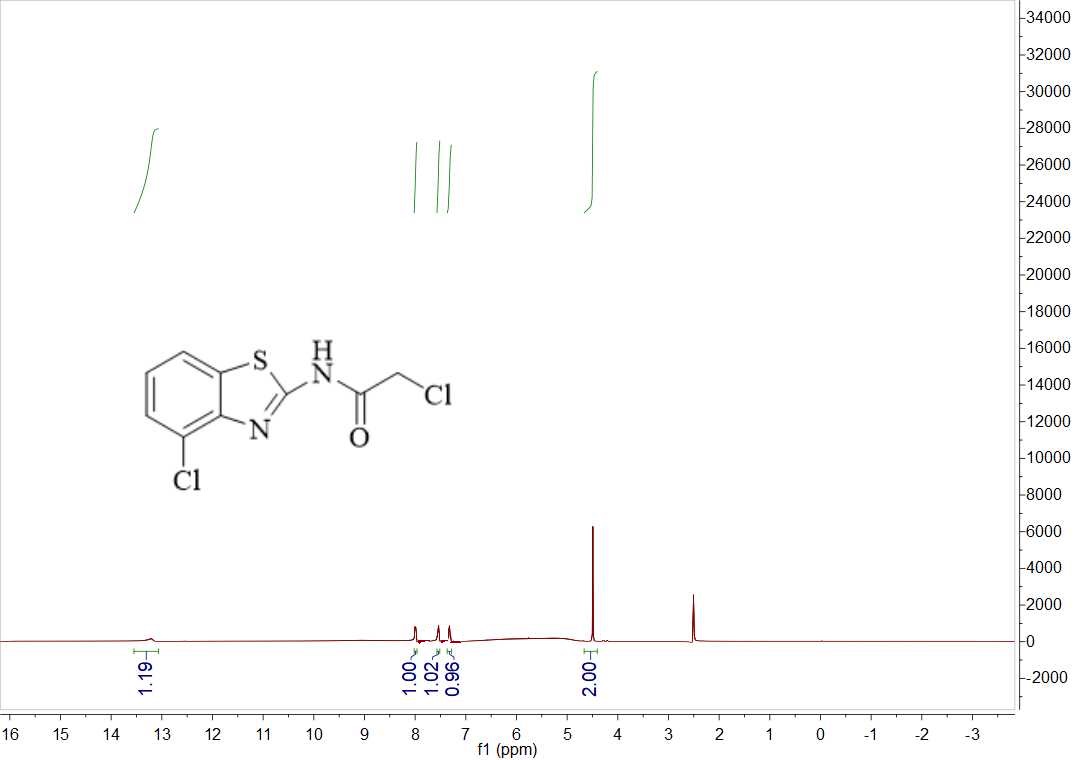
**

**Fig. S9** ^1^H NMR for compound **BTL-3**

**
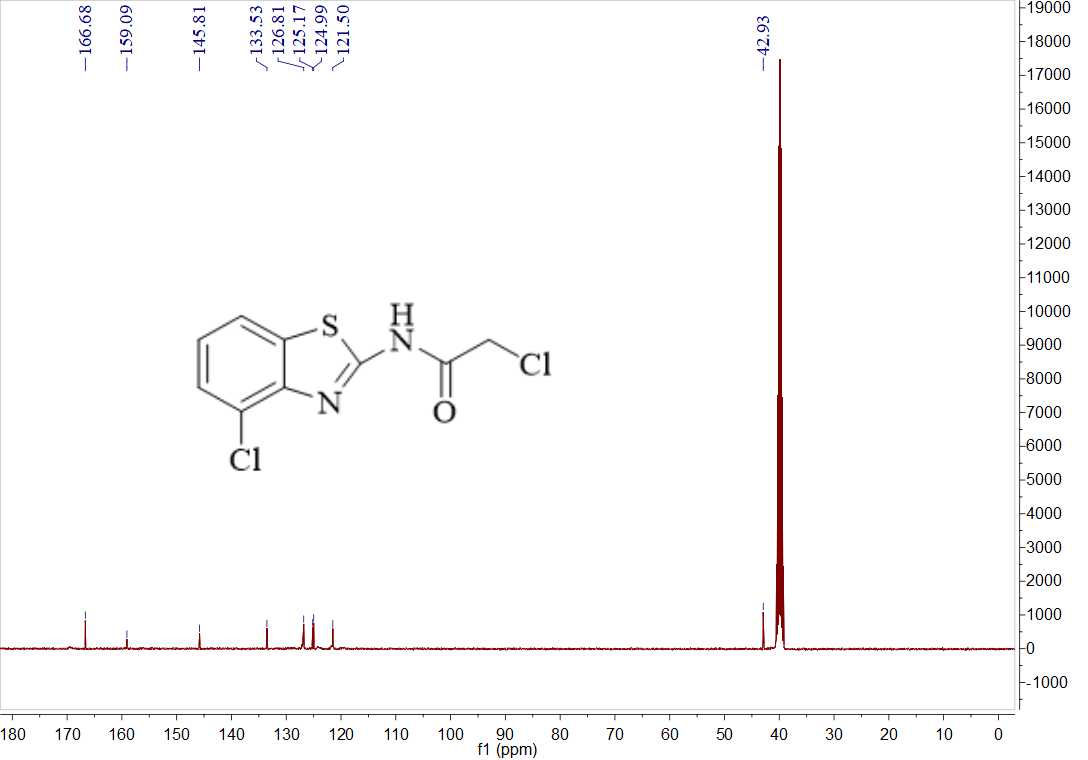
**

**Fig. S10** ^13^C NMR for compound **BTL-3**

**
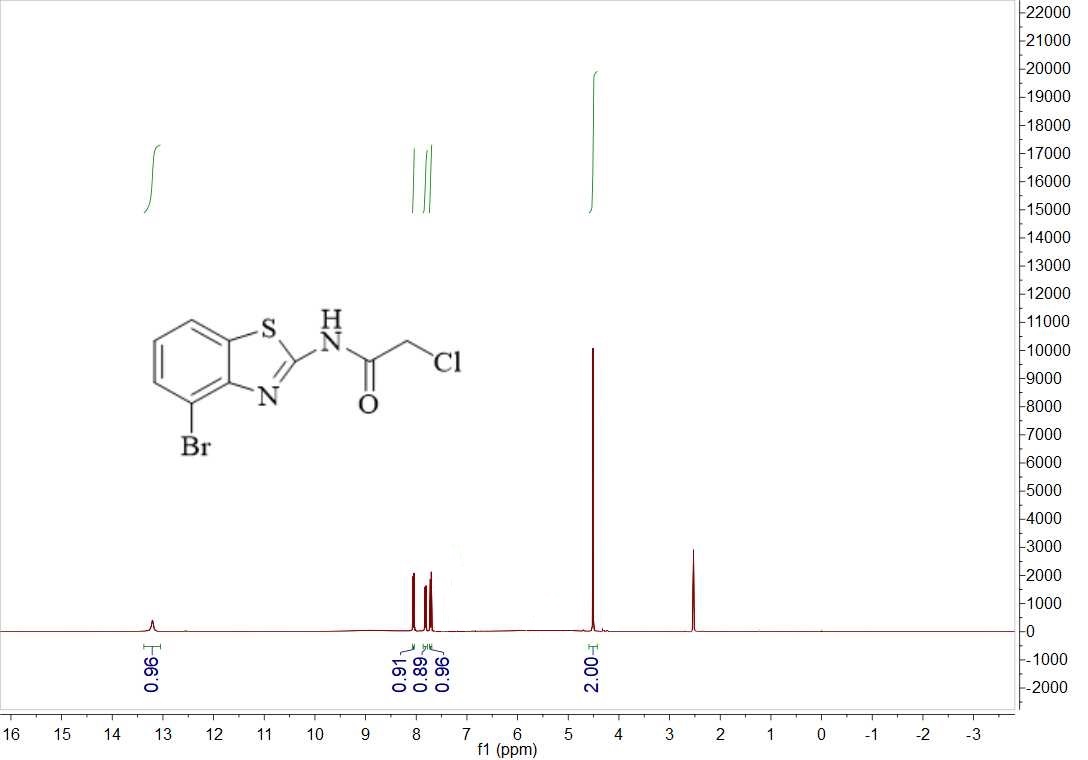
**

**Fig. S11** ^1^H NMR for compound **BTL-4**

**
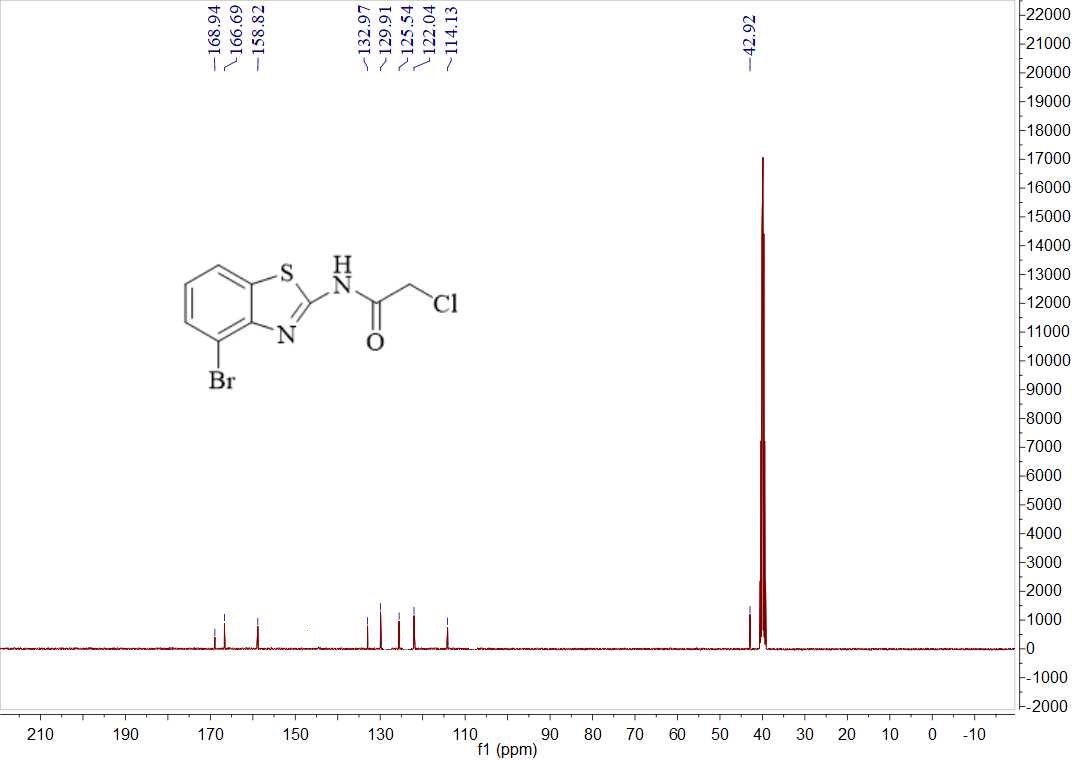
**

**Fig. S12** ^13^C NMR for compound **BTL-4**

**
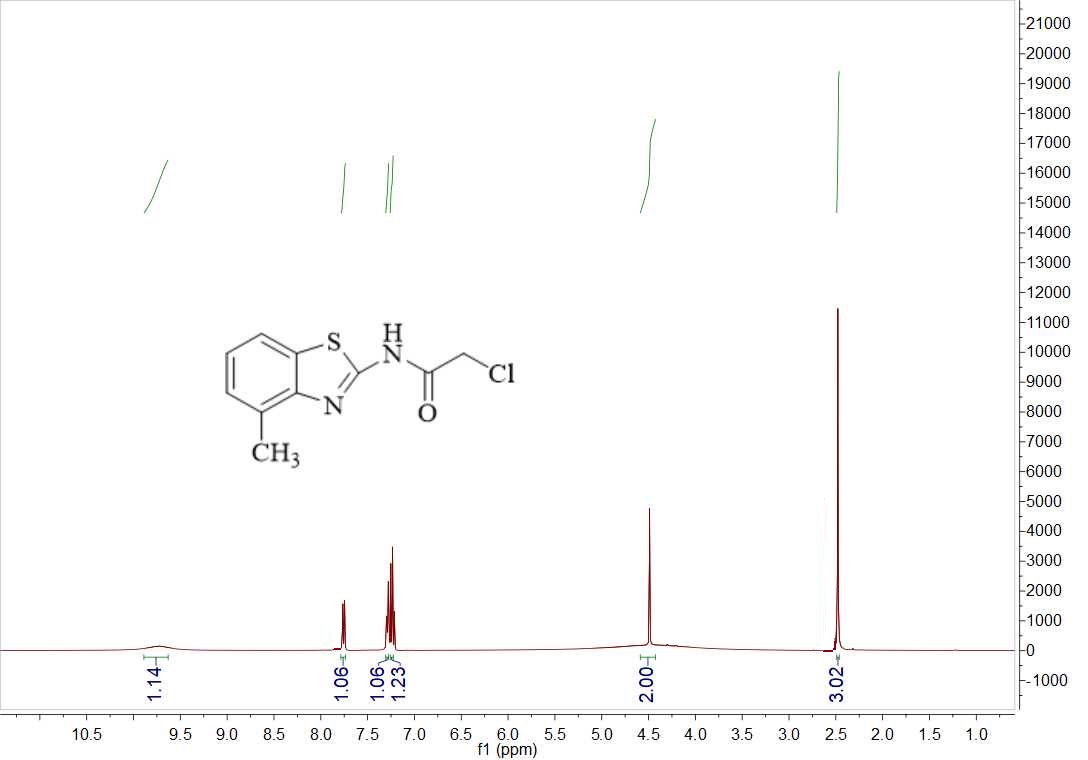
**

**Fig. S13** ^1^H NMR for compound **BTL-5**

**
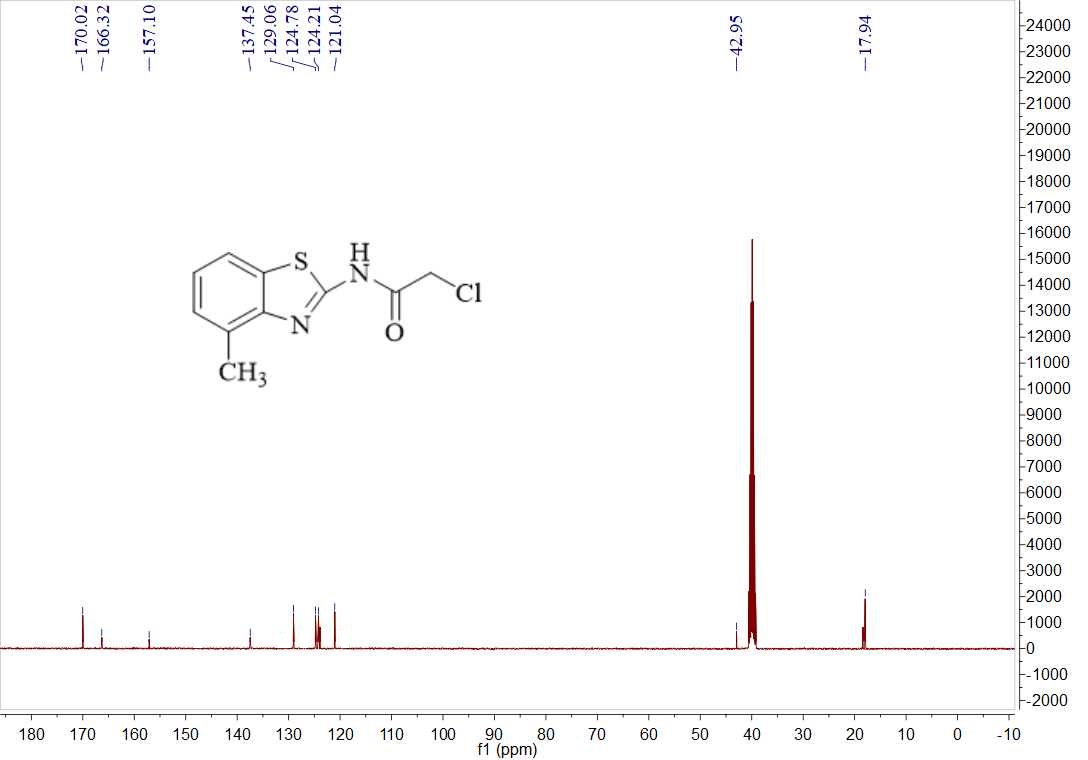
**

**Fig. S14** ^13^C NMR for compound **BTL-5**

**
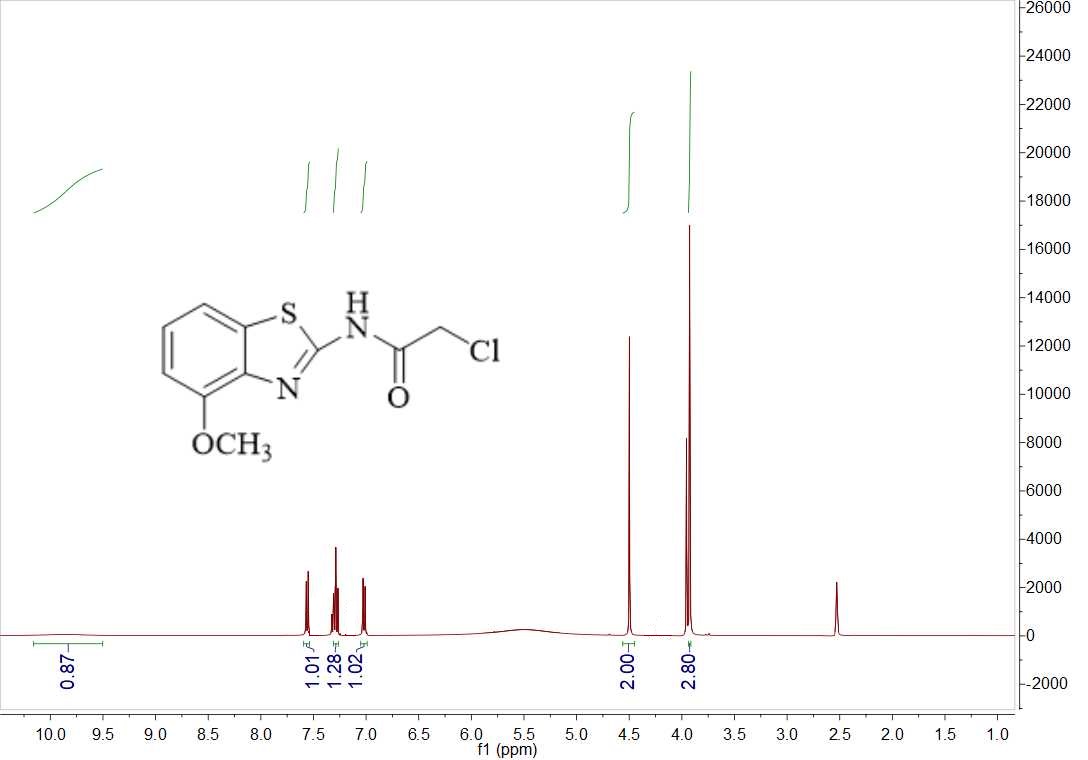
**

**Fig. S15** ^1^H NMR for compound **BTL-6**

**
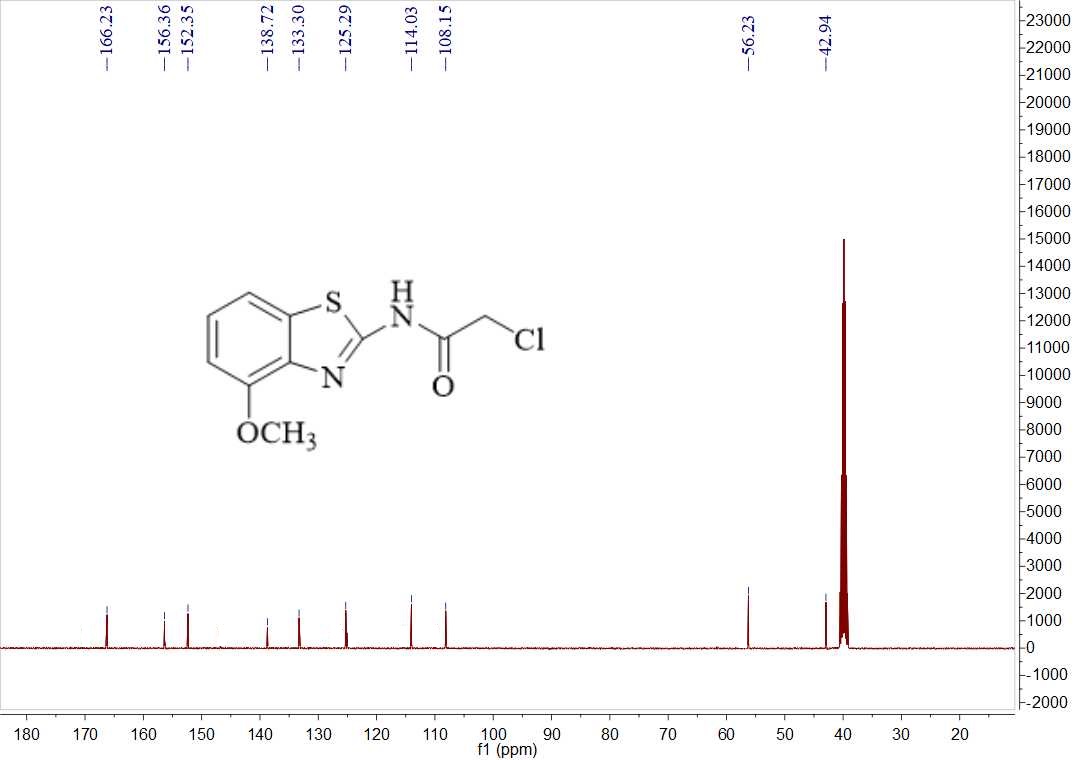
**

**Fig. S16** ^13^C NMR for compound **BTL-6**

**
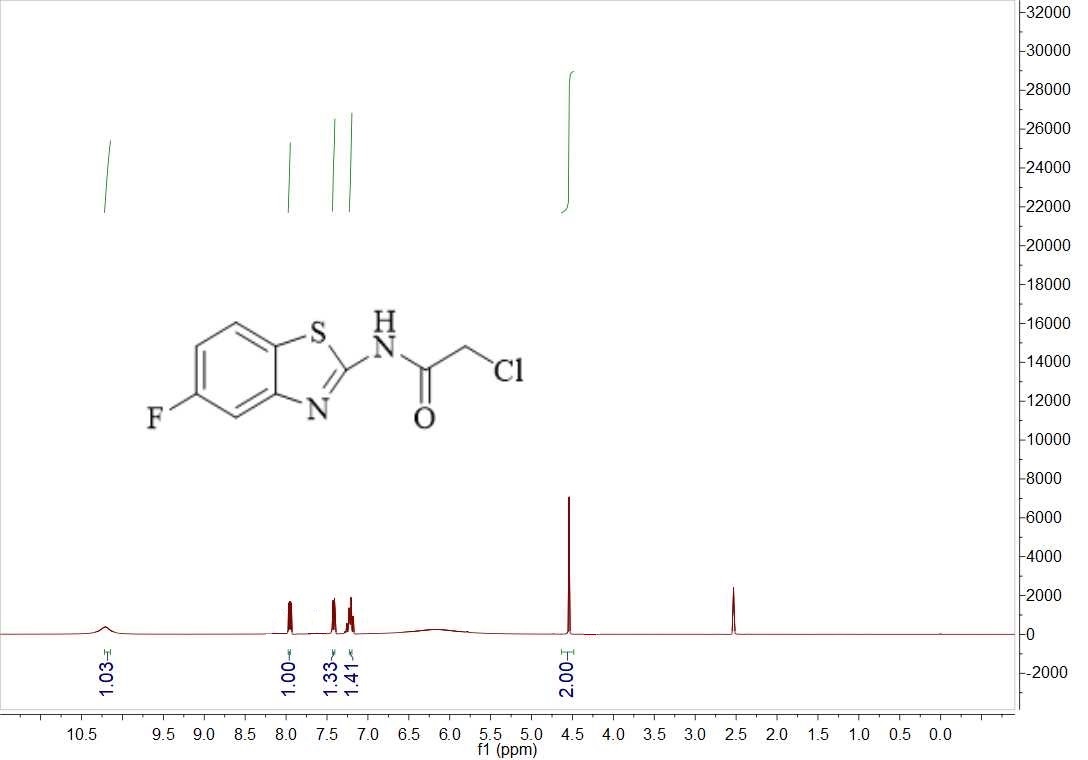
**

**Fig. S17** ^1^H NMR for compound **BTL-7**

**
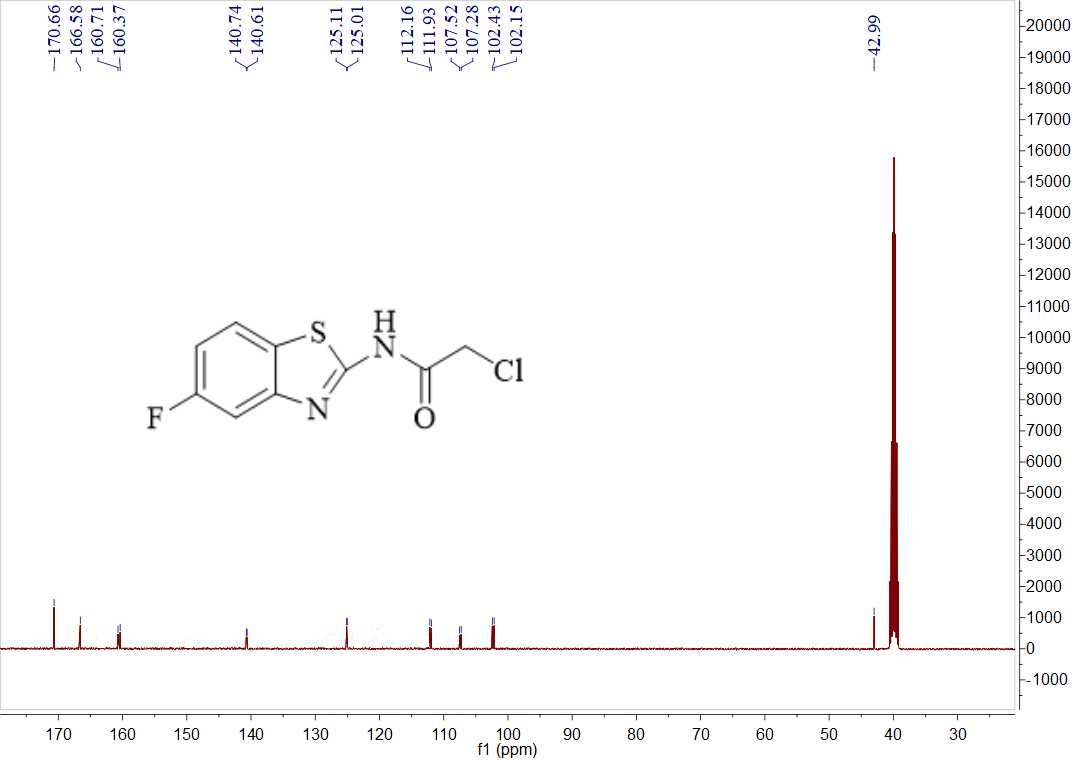
**

**Fig. S18** ^13^C NMR for compound **BTL-7**

**
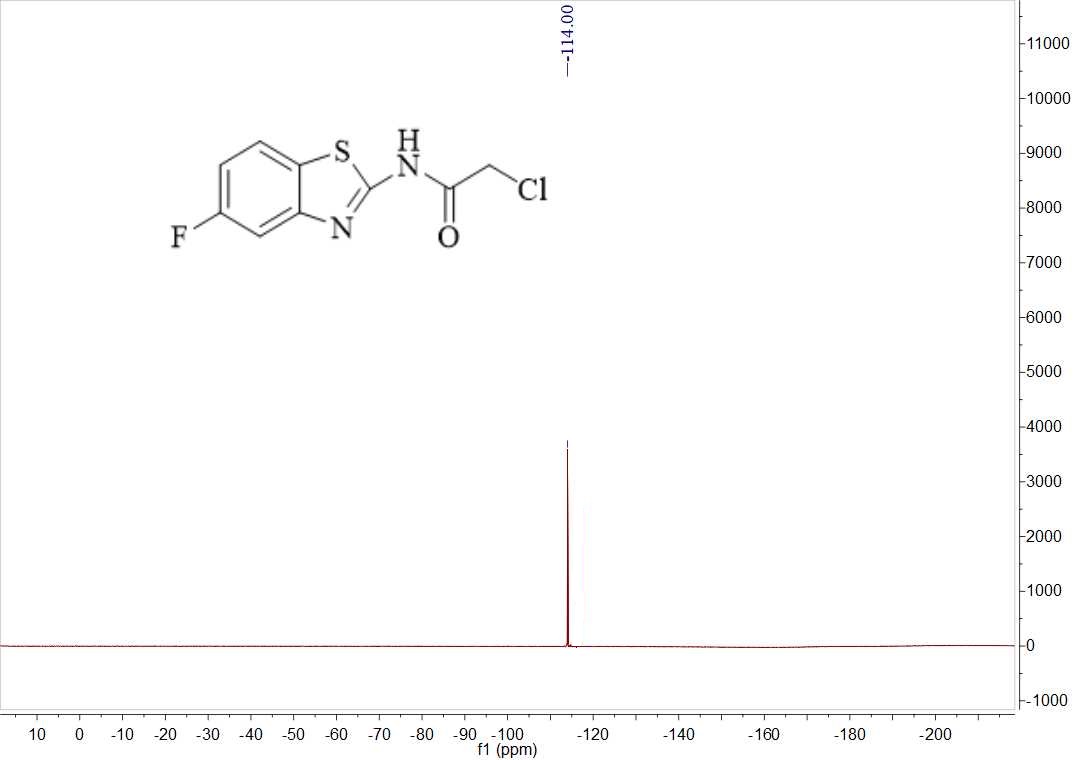
**

**Fig. S19** ^19^F NMR for compound **BTL-7**

**
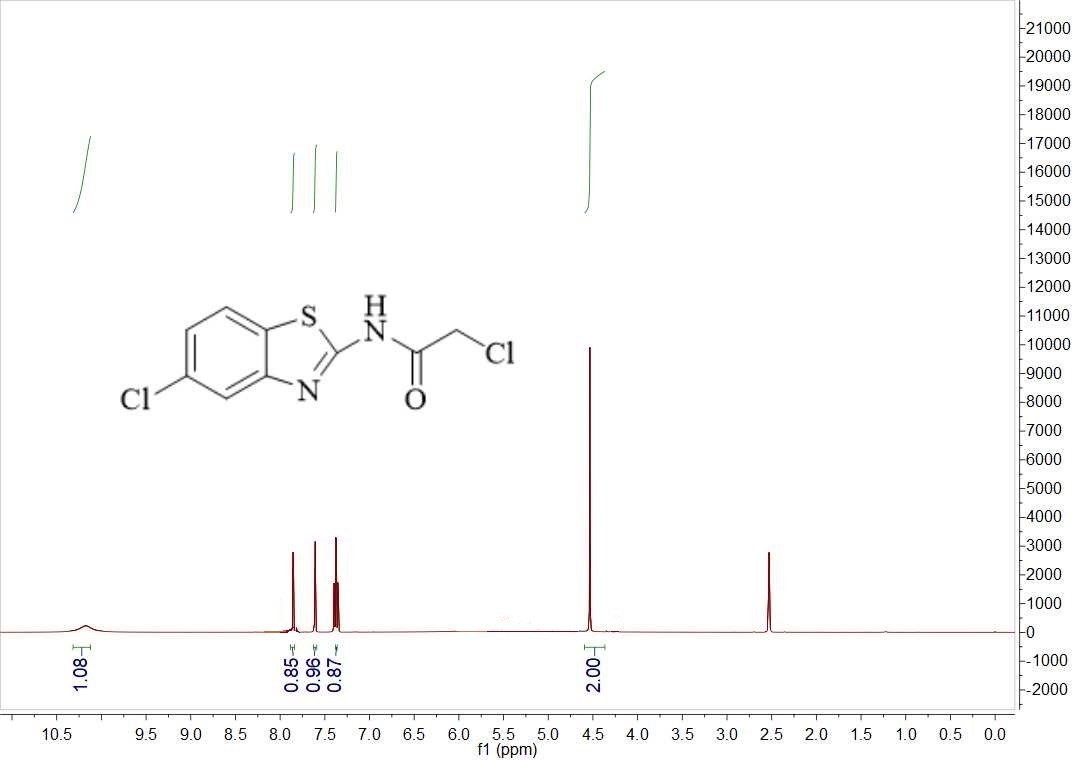
**

**Fig. S20** ^1^H NMR for compound **BTL-8**

**
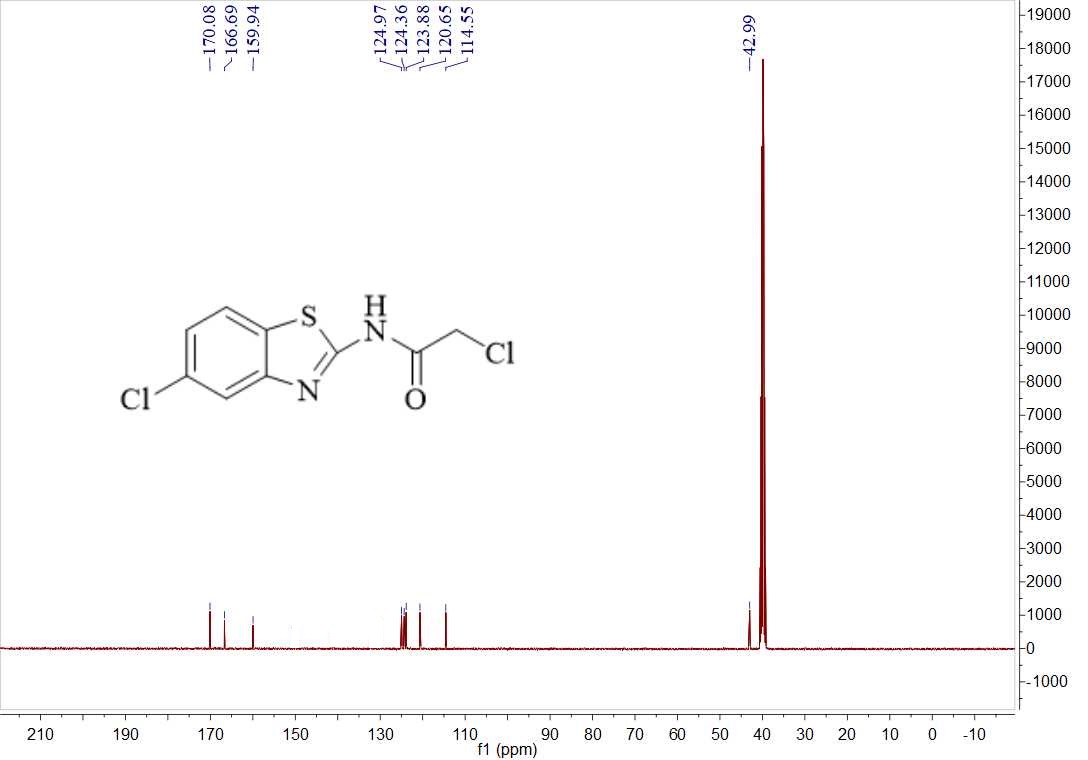
**

**Fig. S21** ^13^C NMR for compound **BTL-8**

**
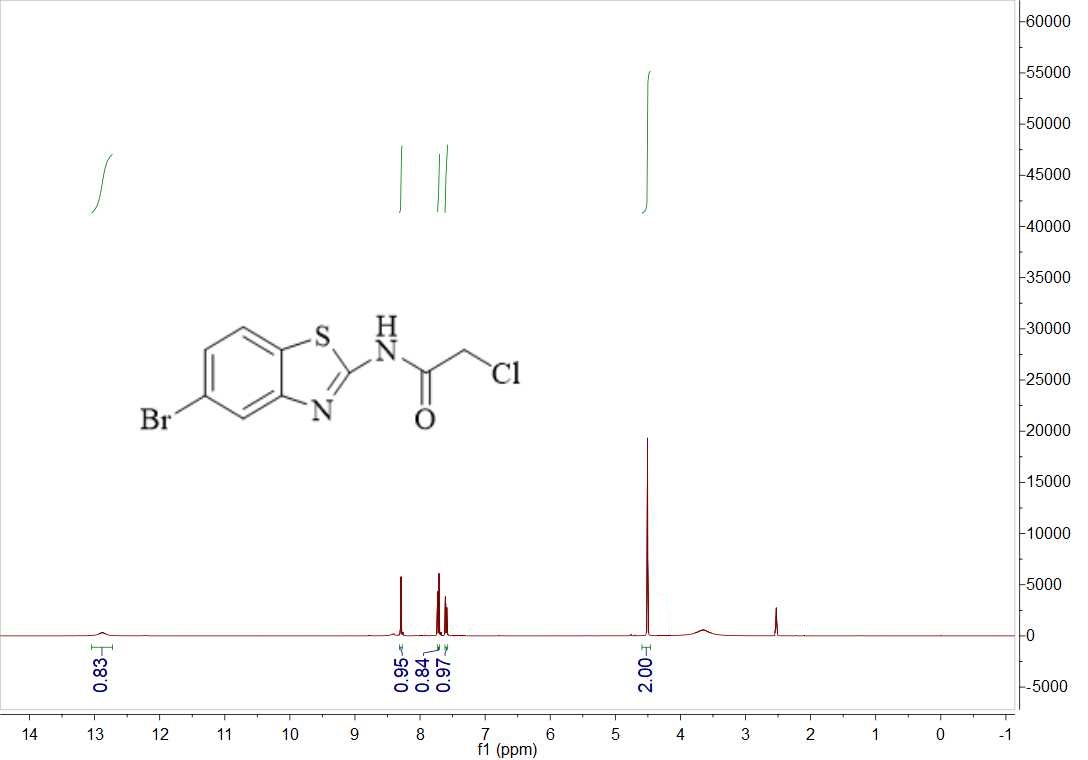
**

**Fig. S22** ^1^H NMR for compound **BTL-9**

**
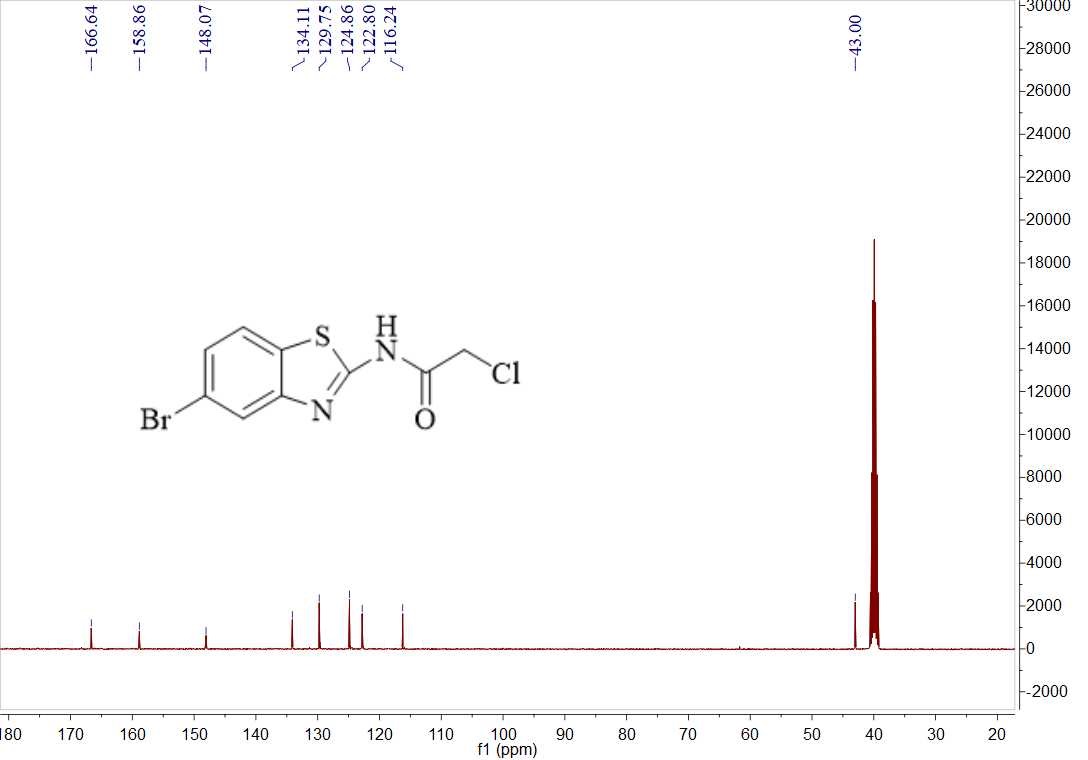
**

**Fig. S23** ^13^C NMR for compound **BTL-9**

**
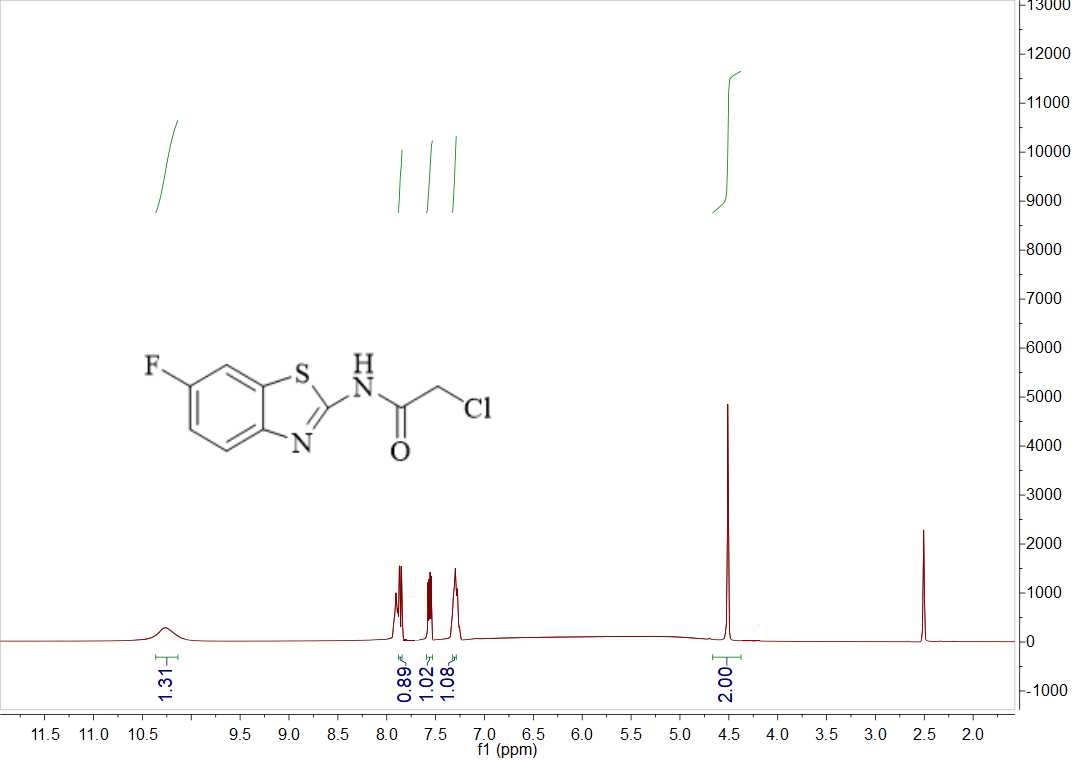
**

**Fig. S24** ^1^H NMR for compound **BTL-10**

**
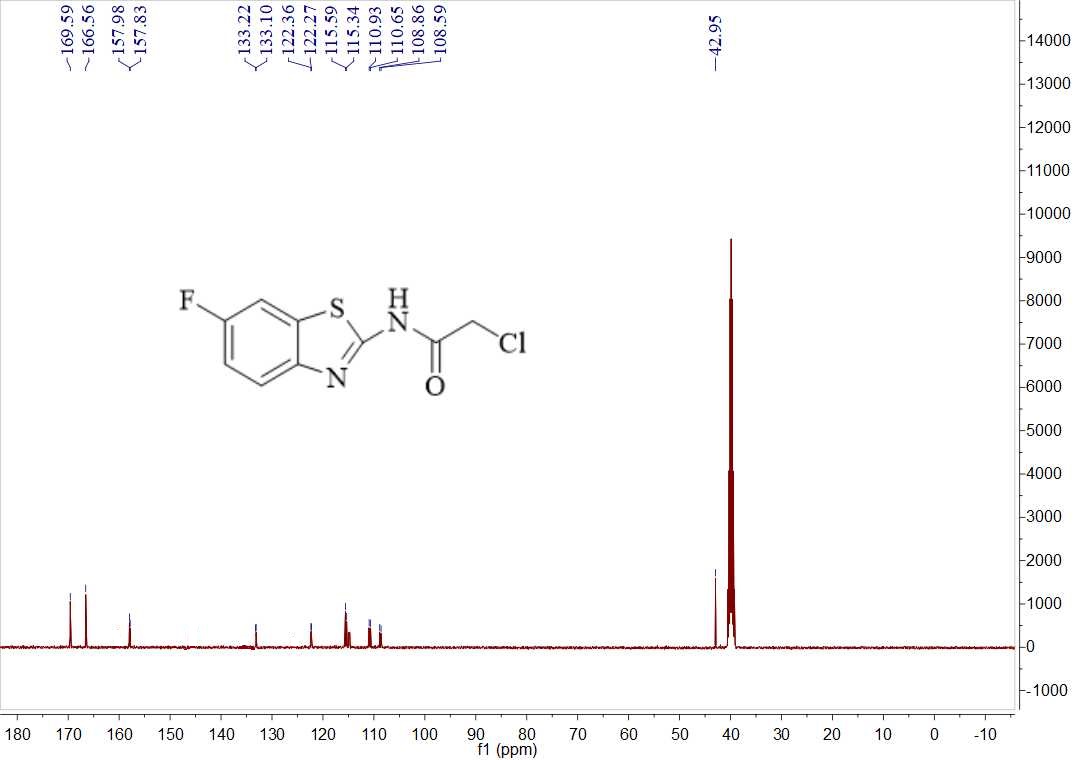
**

**Fig. S25** ^13^C NMR for compound **BTL-10**

**
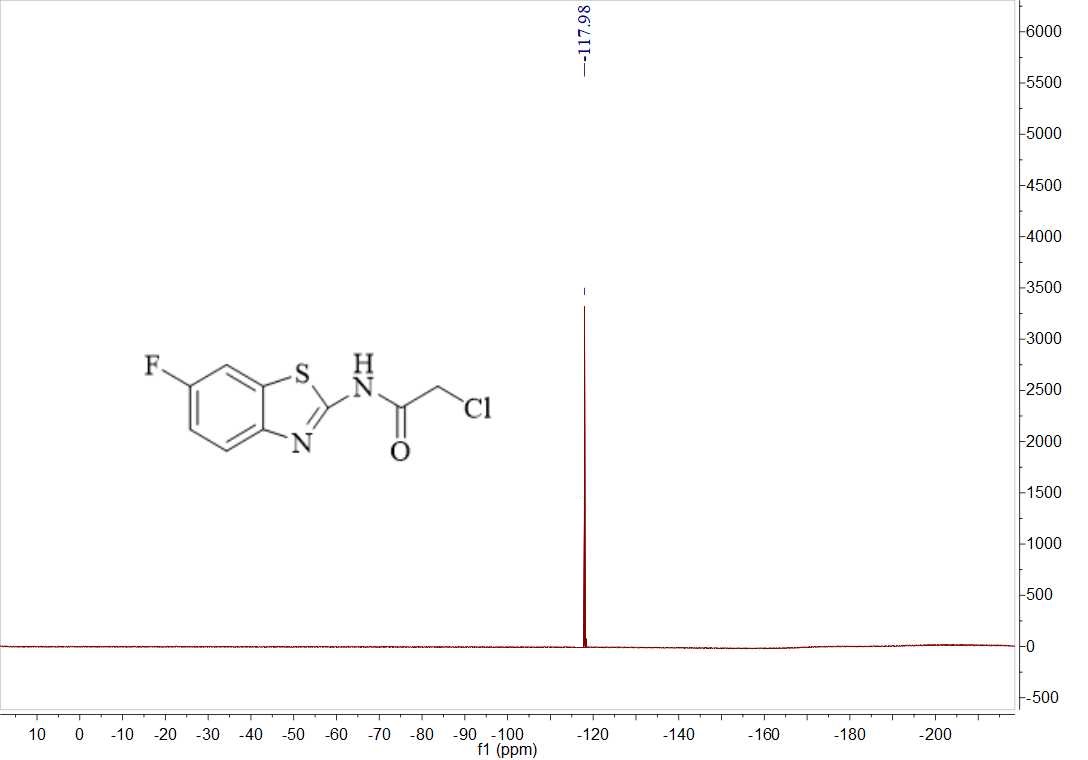
**

**Fig. S26** ^19^F NMR for compound **BTL-10**

**
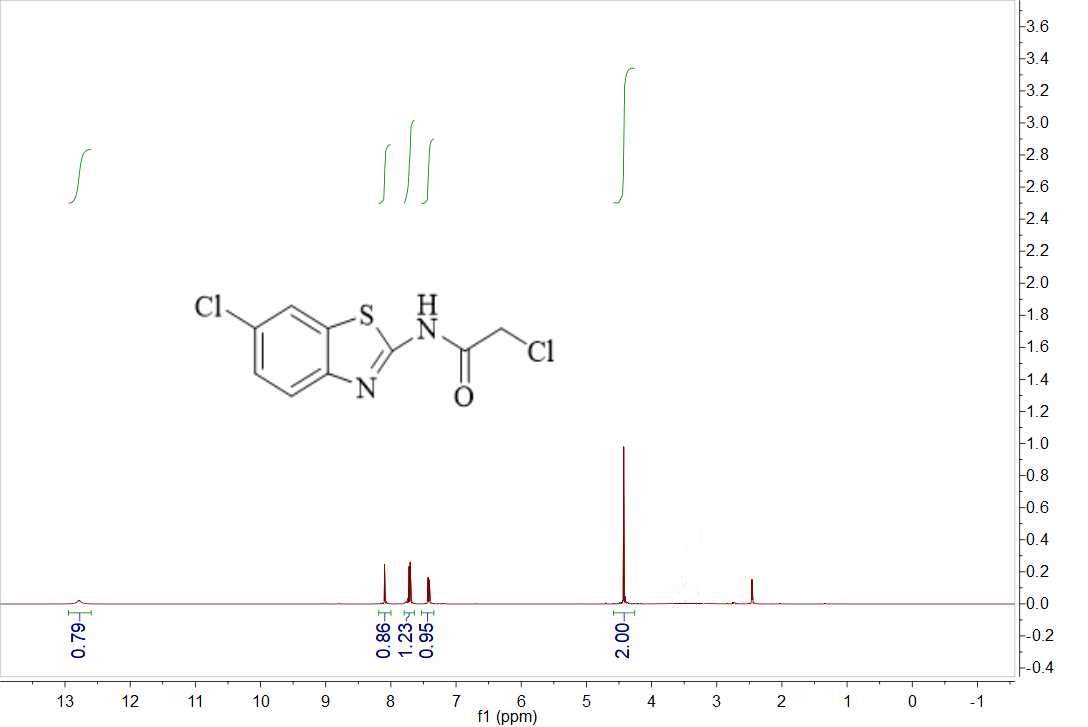
**

**Fig. S27** ^1^H NMR for compound **BTL-11**

**
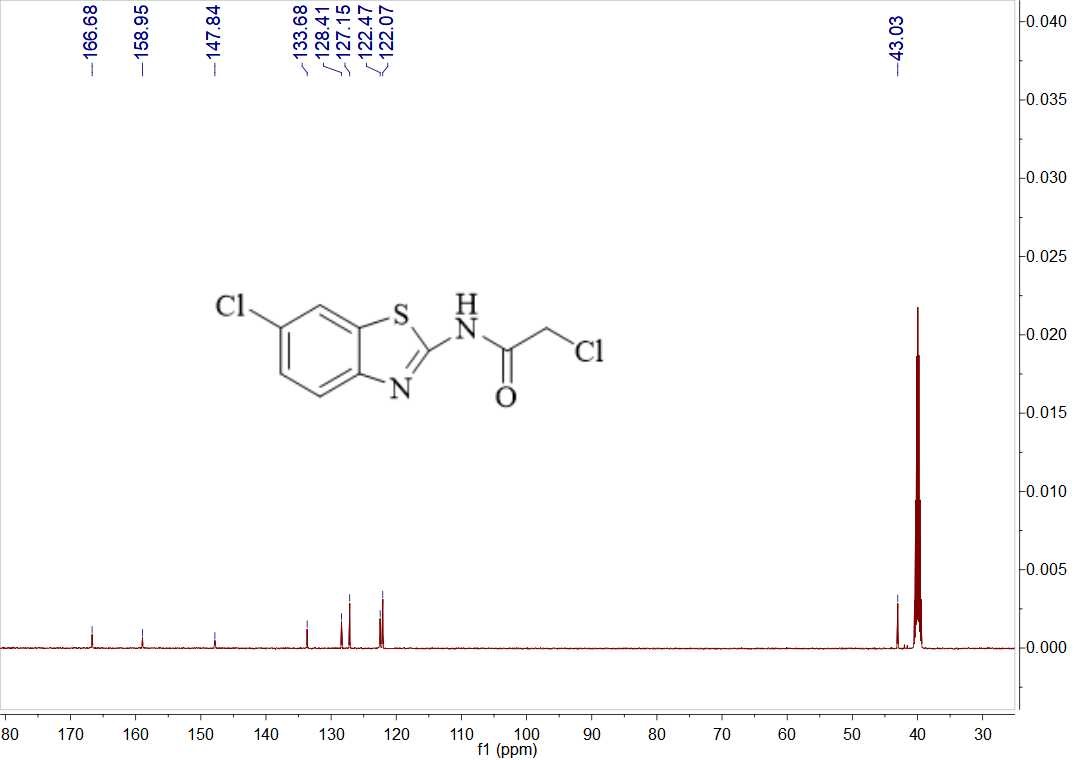
**

**Fig. S28** ^13^C NMR for compound **BTL-11**

**
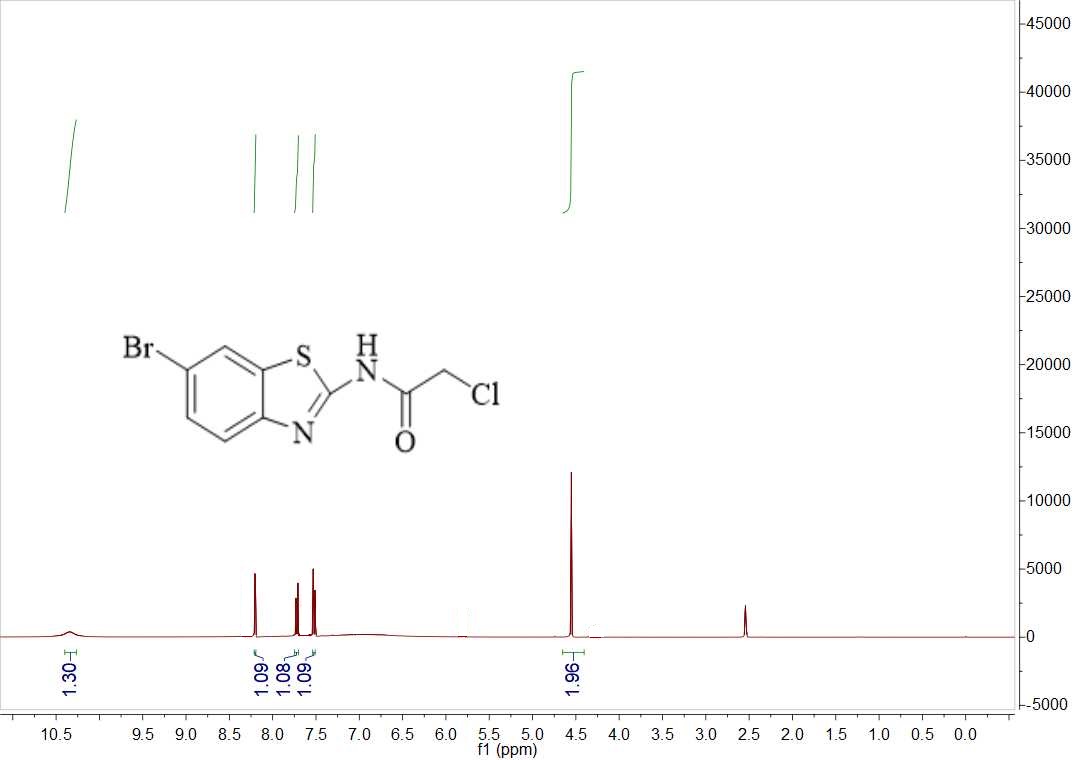
**

**Fig. S29** ^1^H NMR for compound **BTL-12**

**
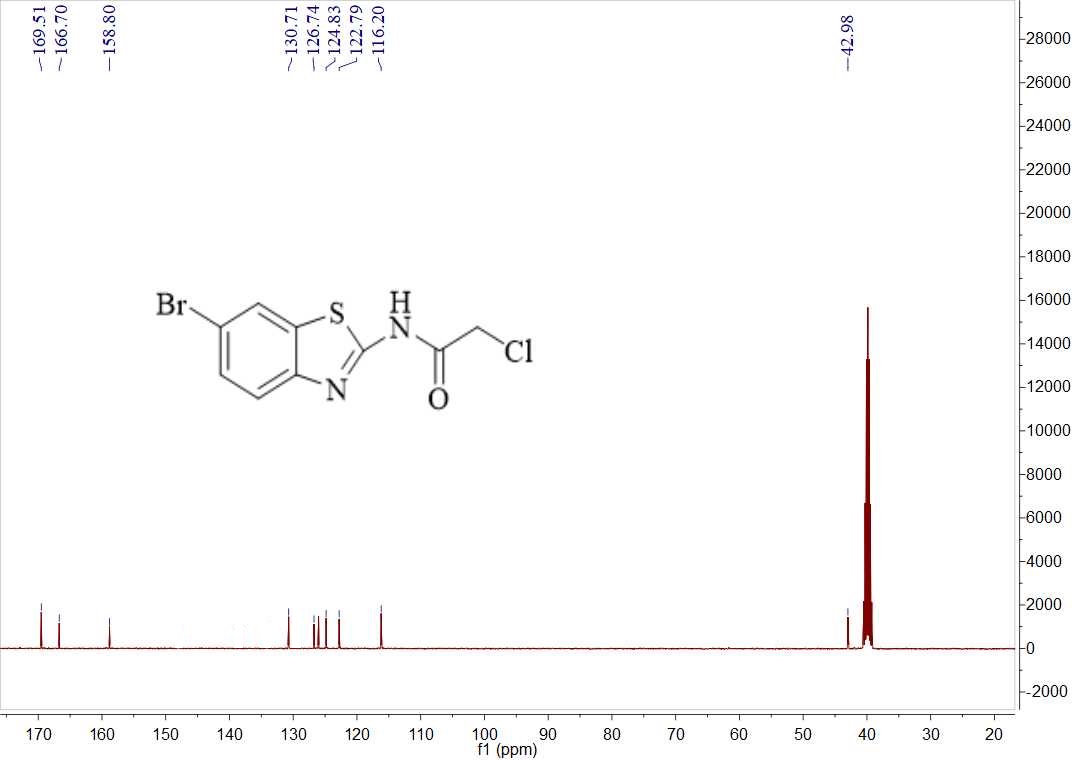
**

**Fig. S30** ^13^C NMR for compound **BTL-12**

**
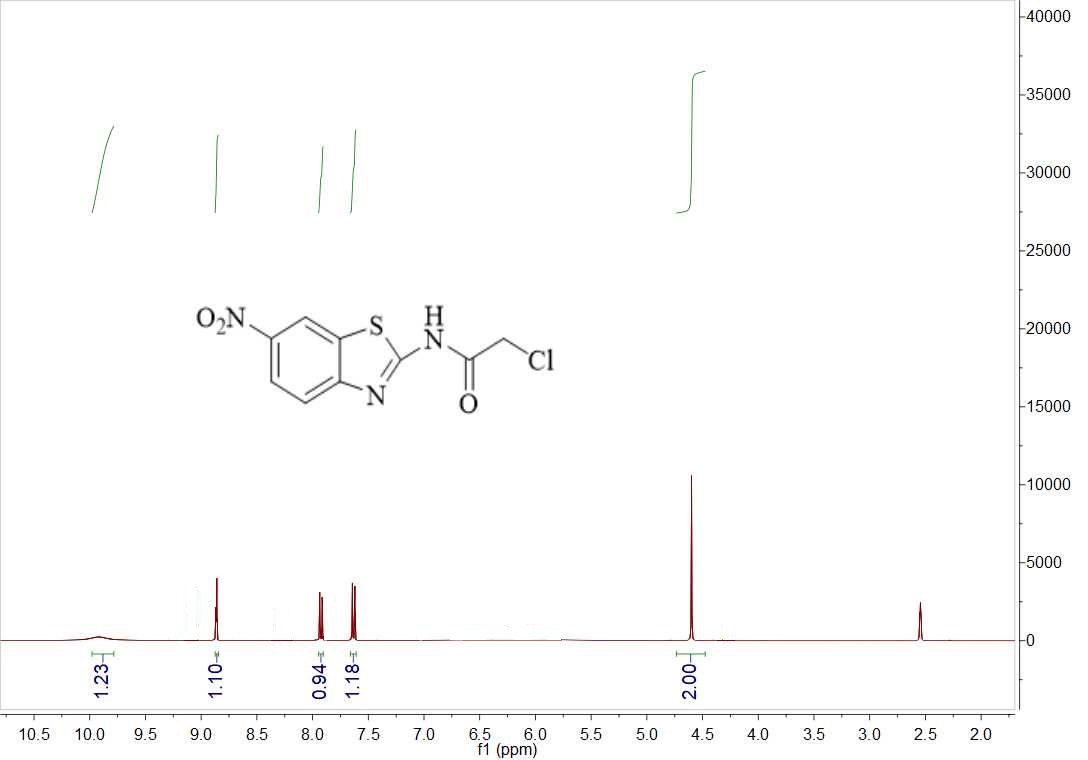
**

**Fig. S31** ^1^H NMR for compound **BTL-13**

**
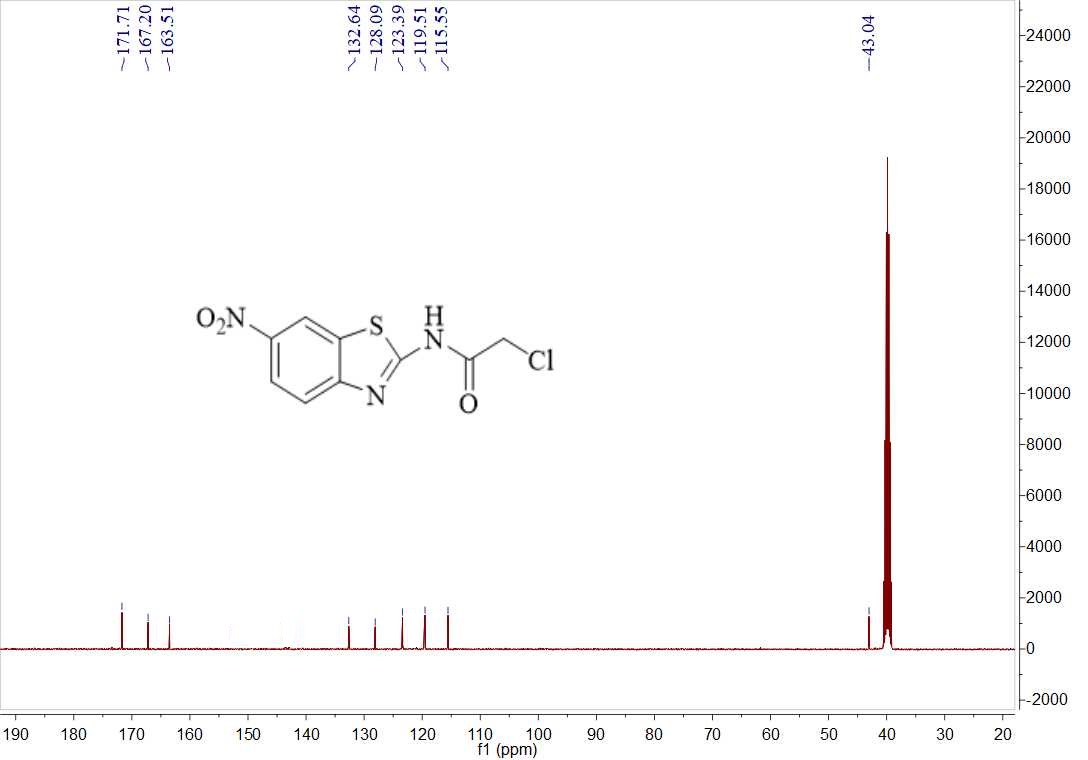
**

**Fig. S32** ^13^C NMR for compound **BTL-13**

**
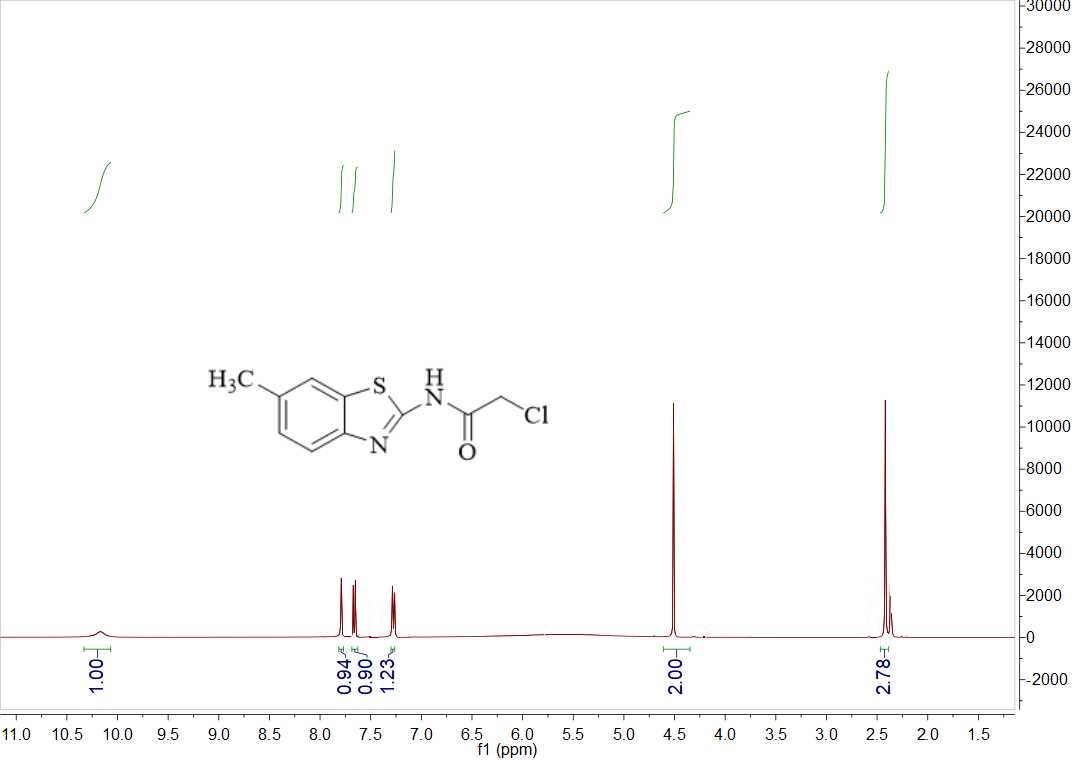
**

**Fig. S33** ^1^H NMR for compound **BTL-14**

**
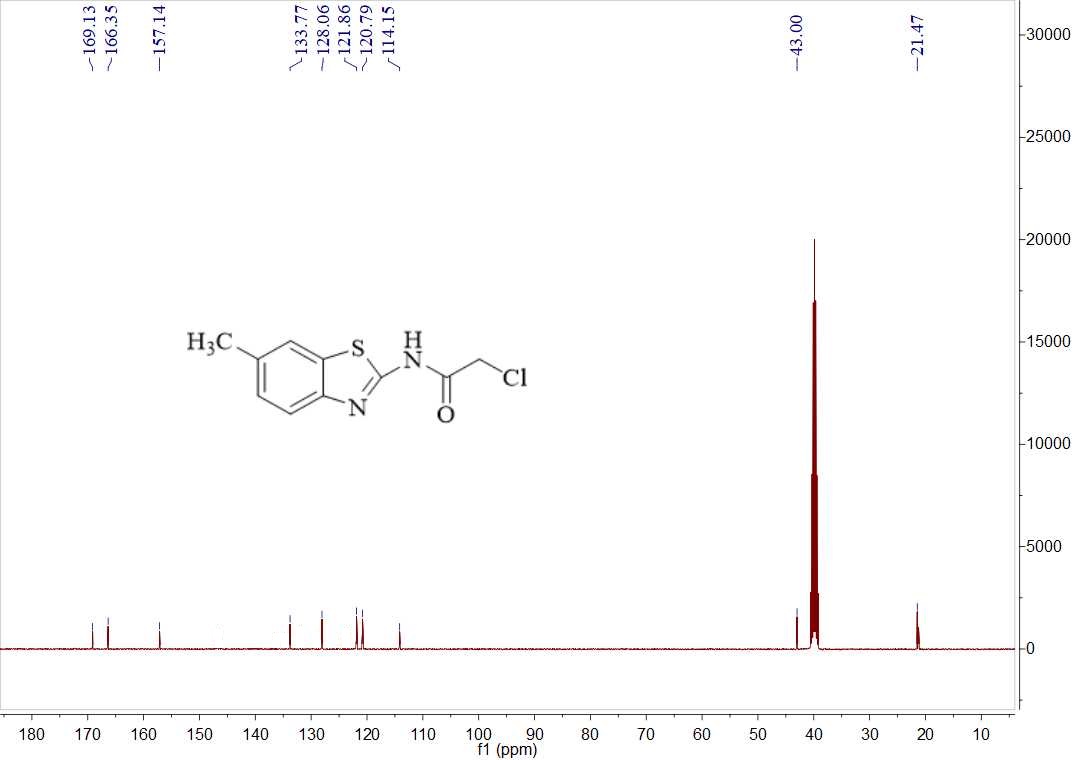
**

**Fig. S34** ^13^C NMR for compound **BTL-14**

**
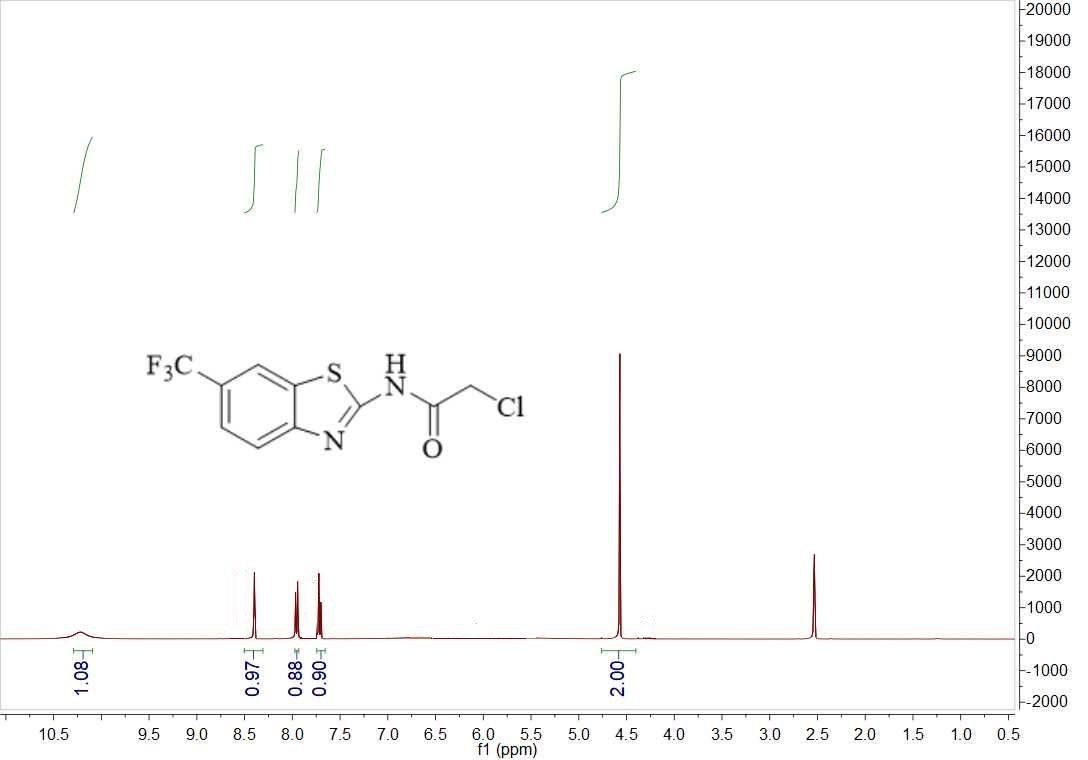
**

**Fig. S35** ^1^H NMR for compound **BTL-15**

**
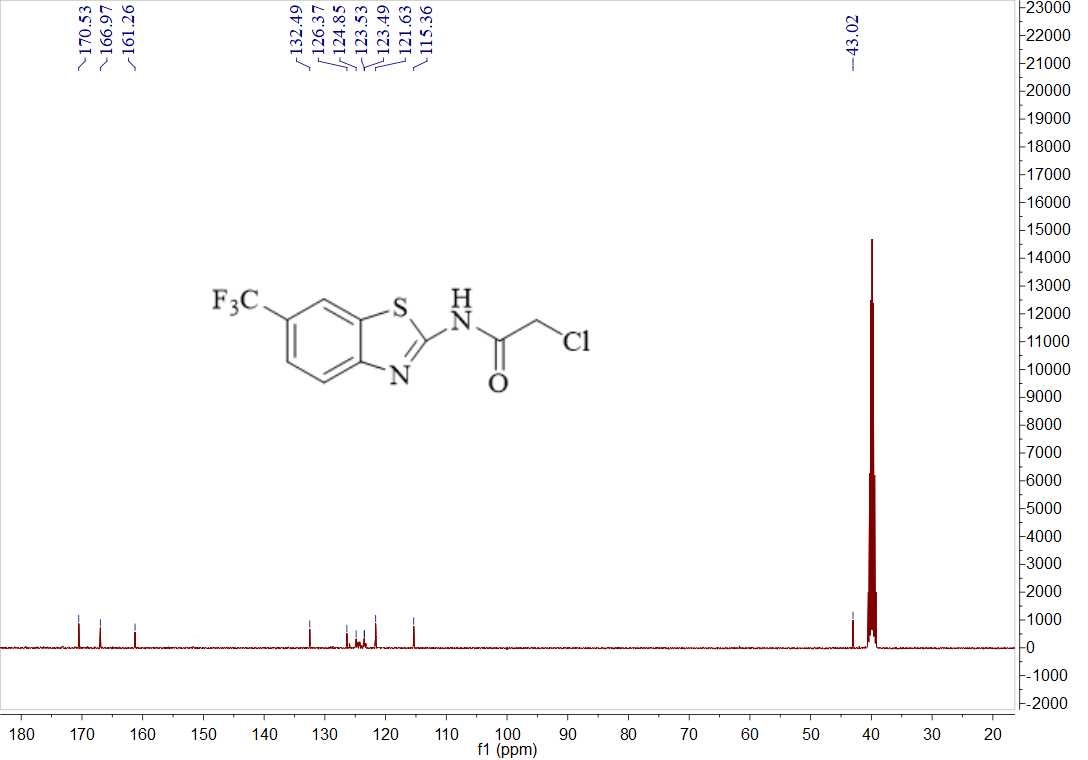
**

**Fig. S36** ^13^C NMR for compound **BTL-15**

**
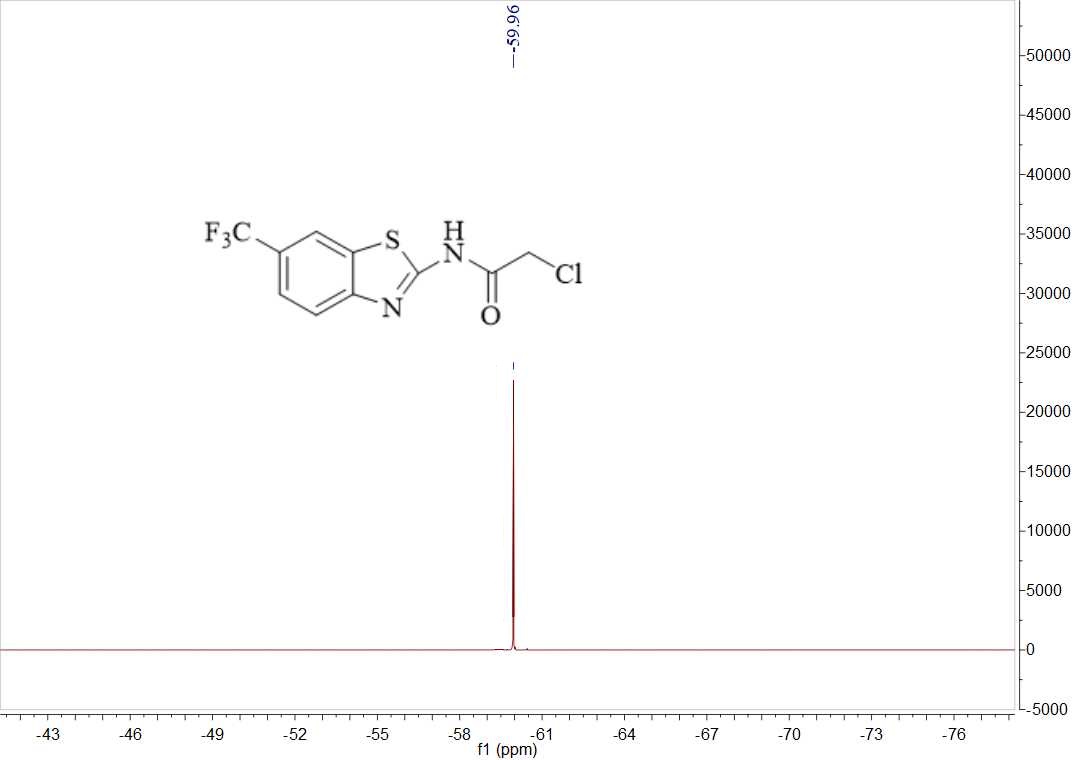
**

**Fig. S37** ^19^F NMR for compound **BTL-15**

**
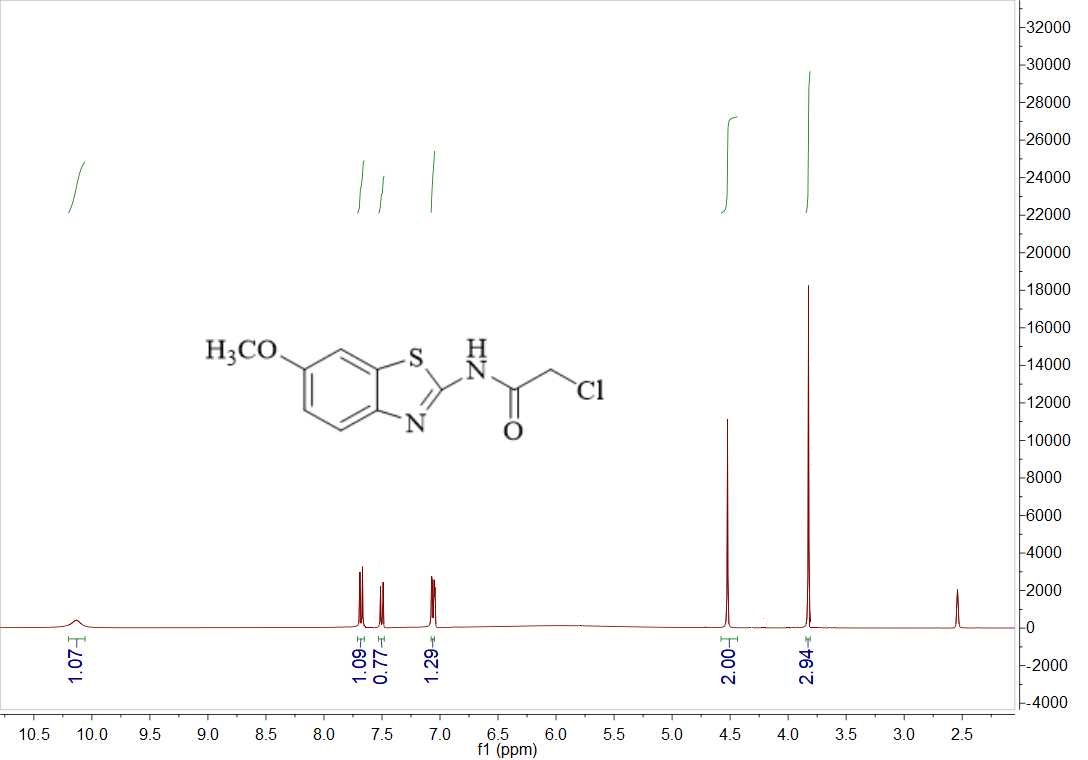
**

**Fig. S38** ^1^H NMR for compound **BTL-16**

**
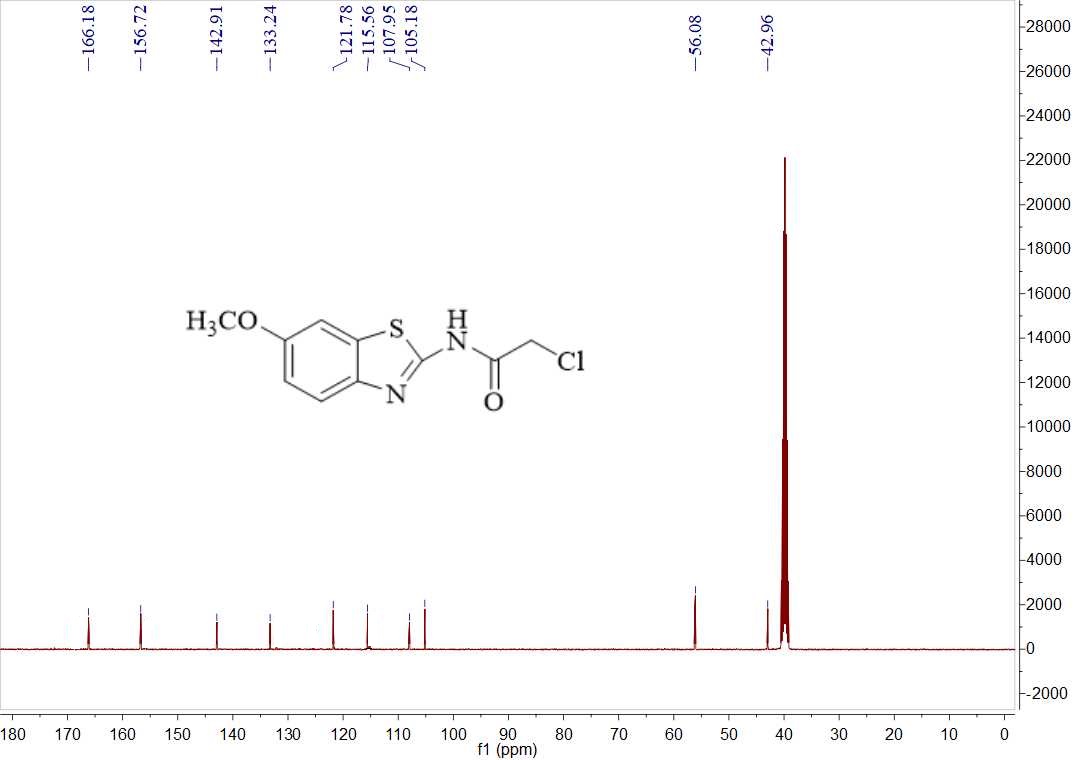
**

**Fig. S39** ^13^C NMR for compound **BTL-16**

**
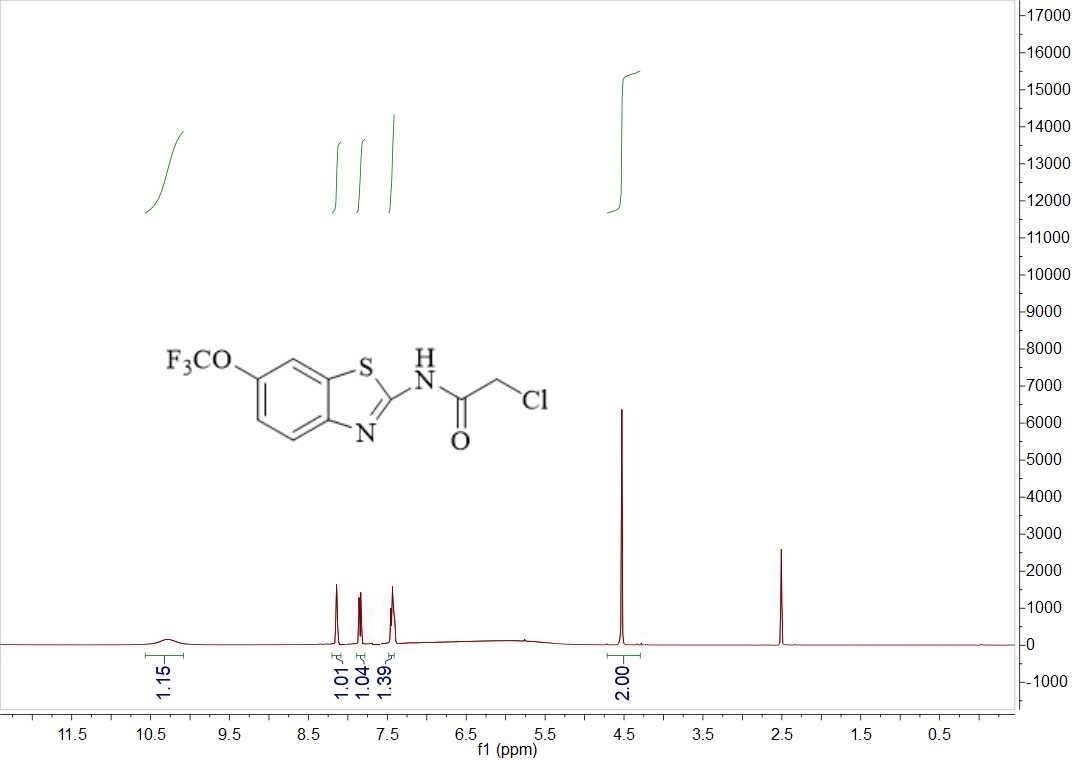
**

**Fig. S40** ^1^H NMR for compound **BTL-17**

**
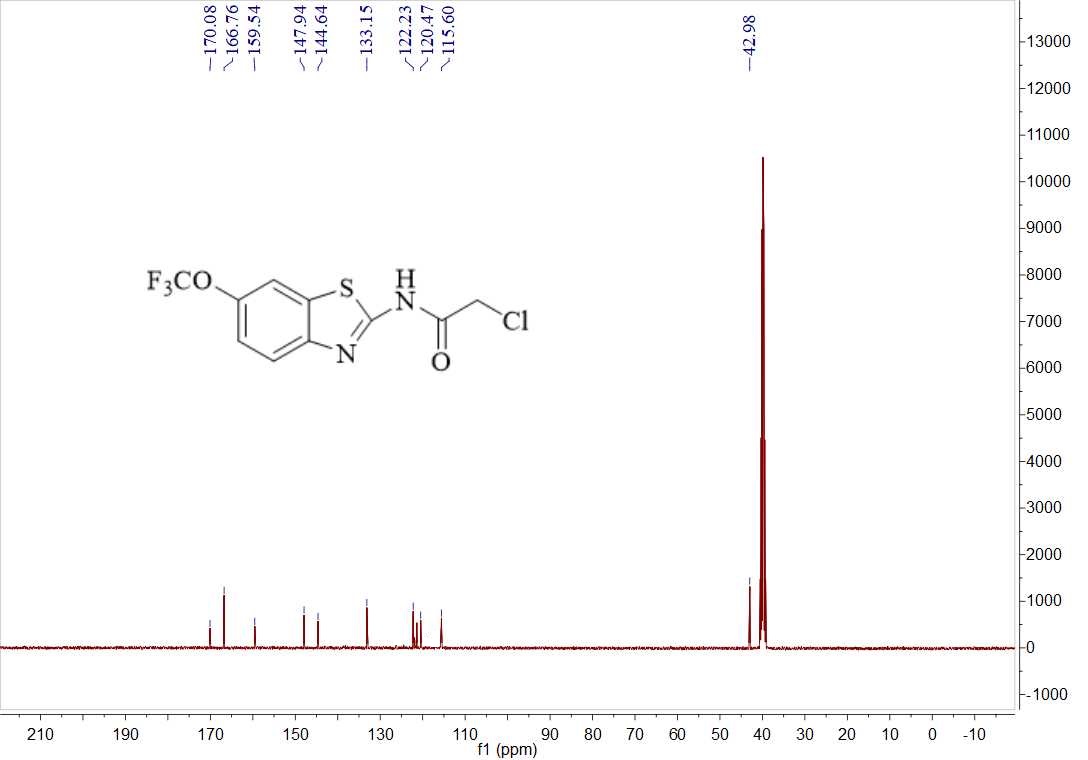
**

**Fig. S41** ^13^C NMR for compound **BTL-17**

**
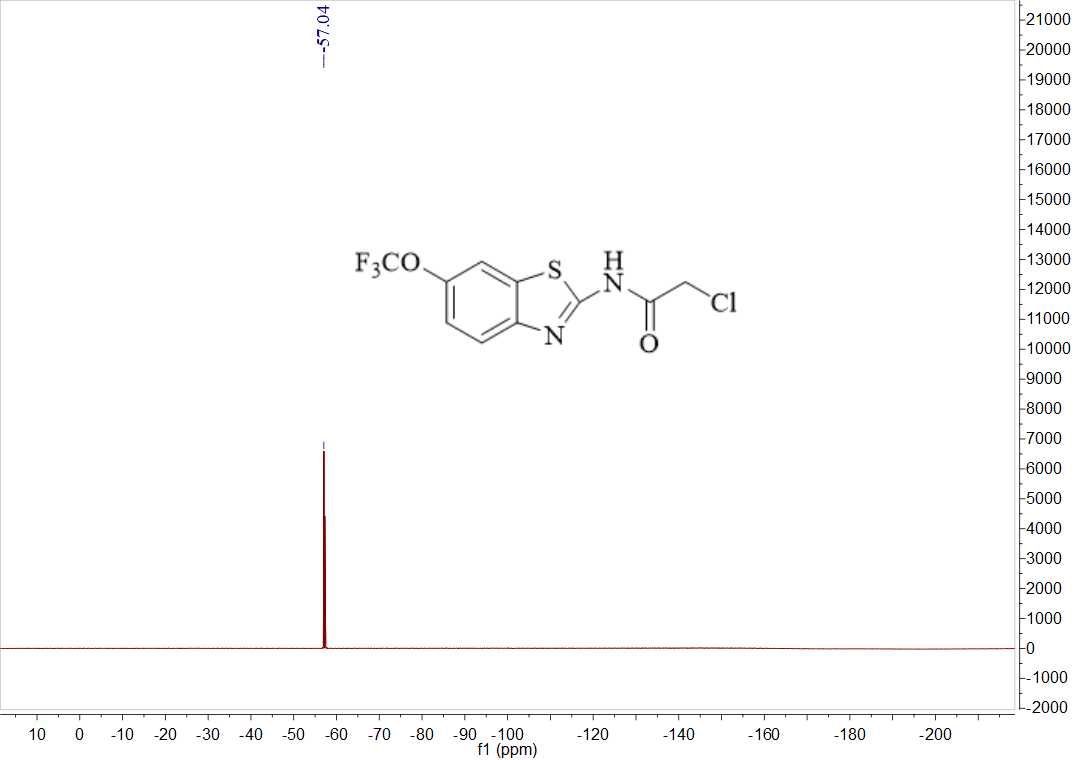
**

**Fig. S42** ^19^F NMR for compound **BTL-17**

**
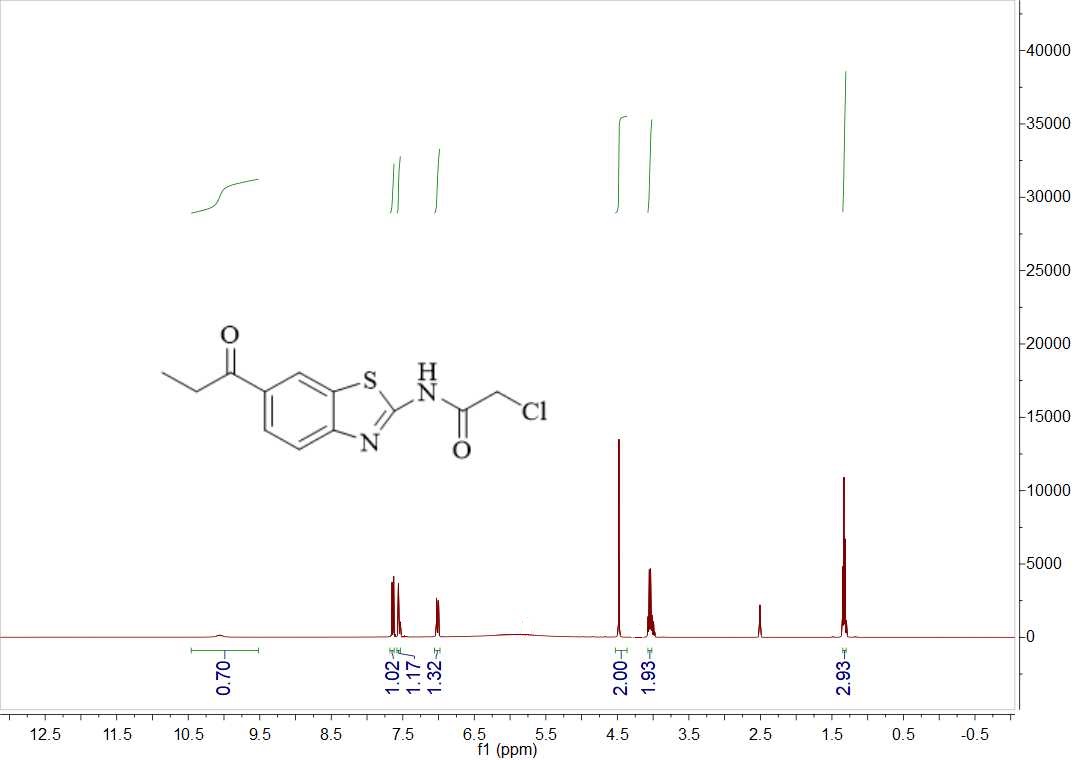
**

**Fig. S43** ^1^H NMR for compound **BTL-18**

**
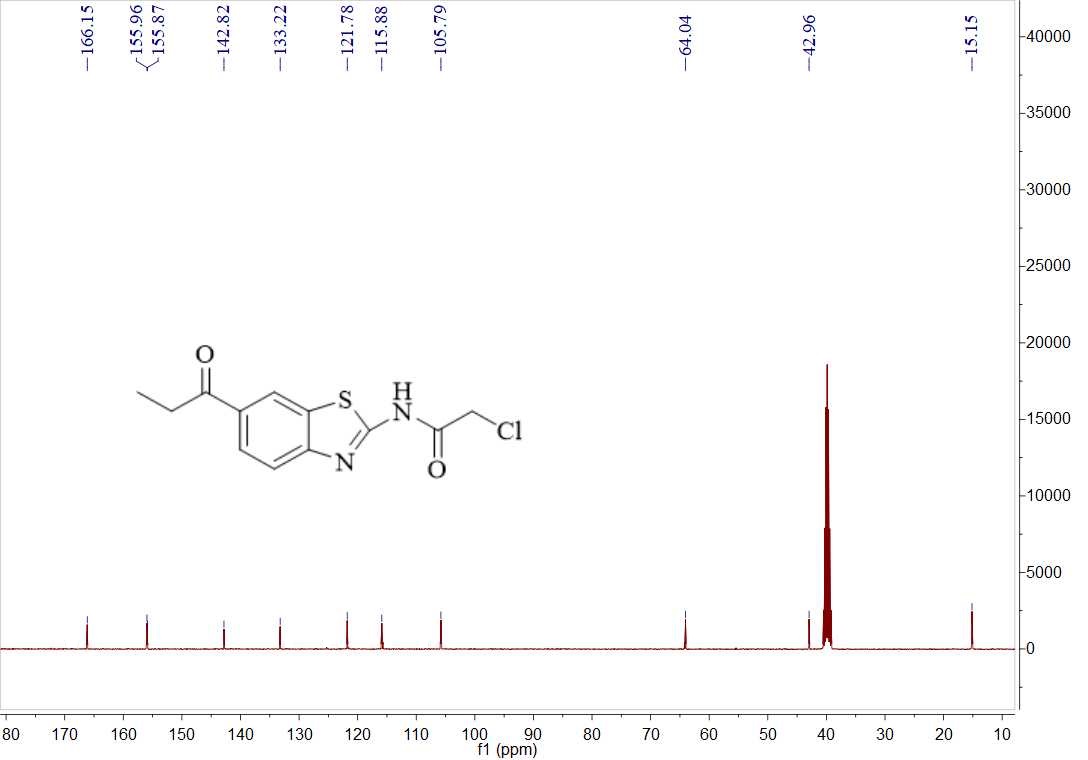
**

**Fig. S44** ^13^C NMR for compound **BTL-18**

**
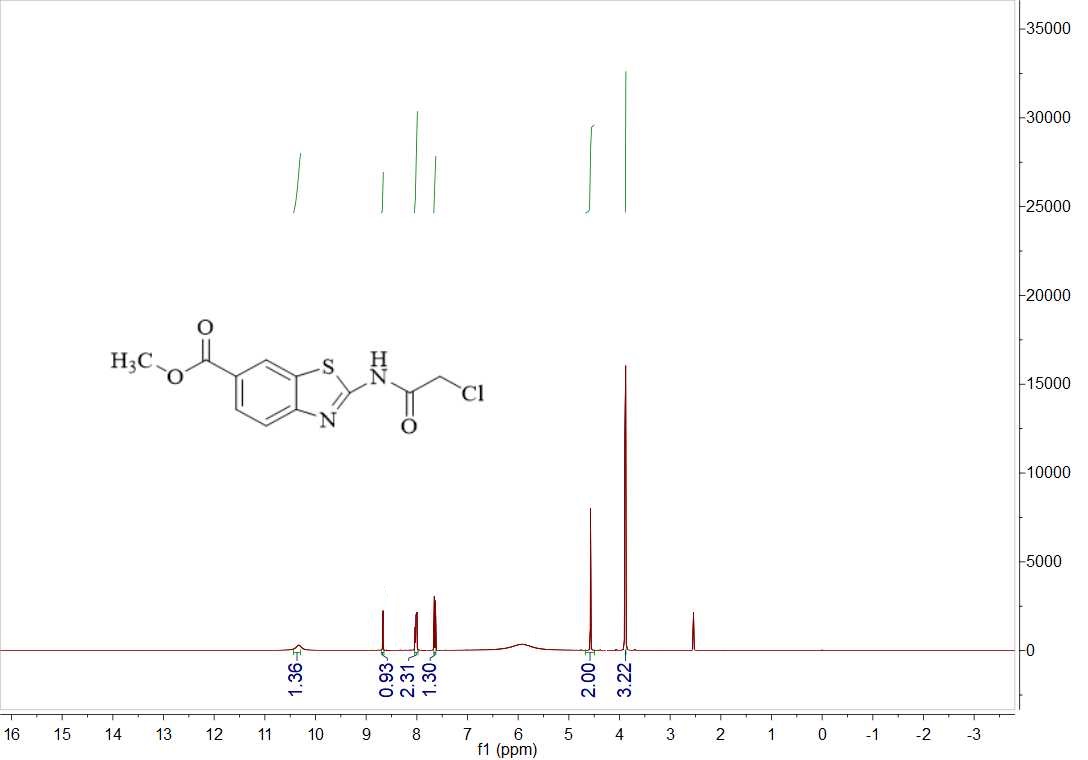
**

**Fig. S45** ^1^H NMR for compound **BTL-19**

**
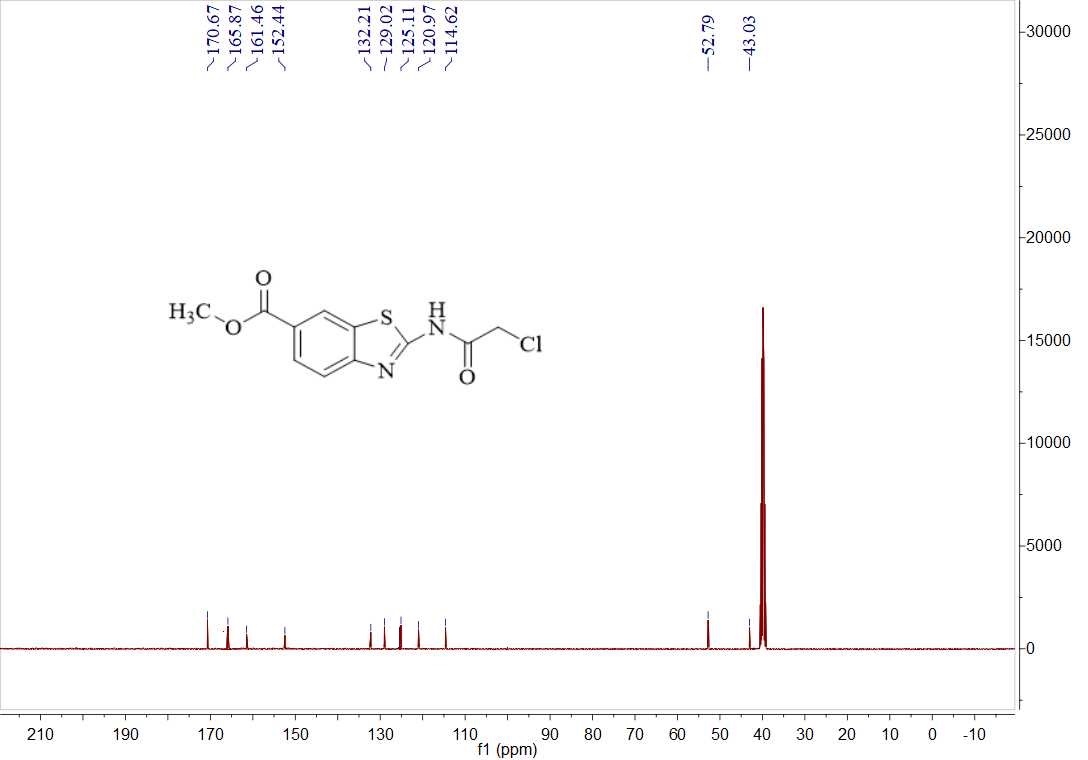
**

**Fig. S46** ^13^C NMR for compound **BTL-19**

**
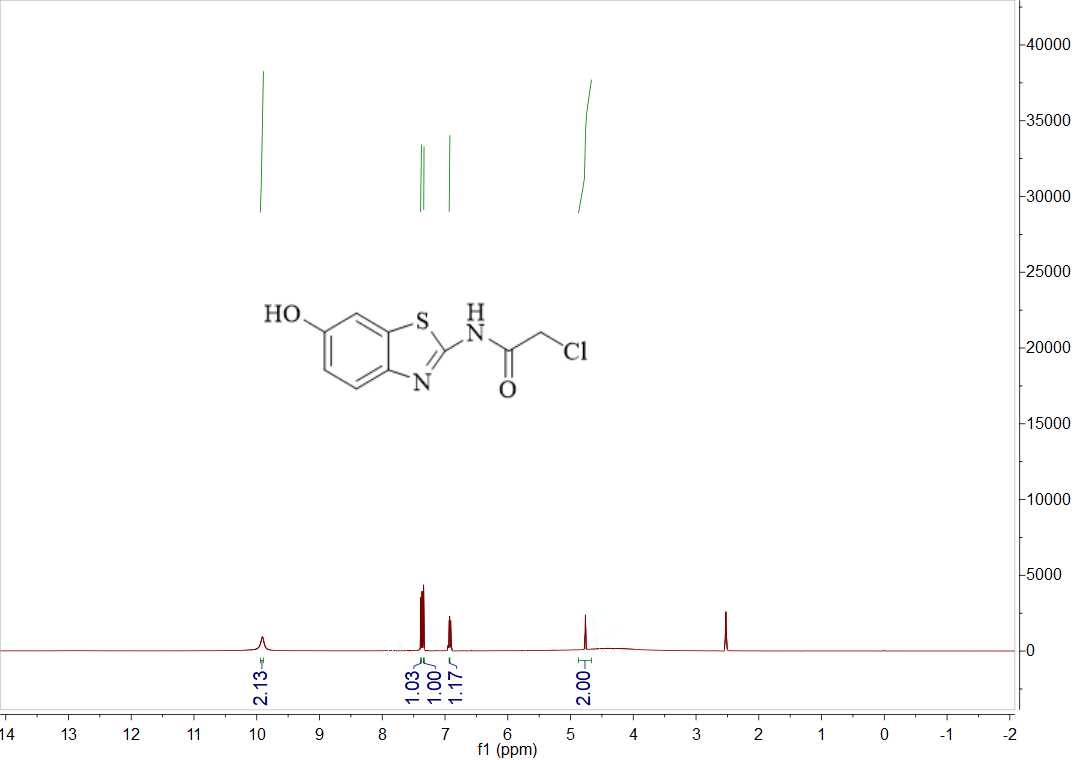
**

**Fig. S47** ^1^H NMR for compound **BTL-20**

**
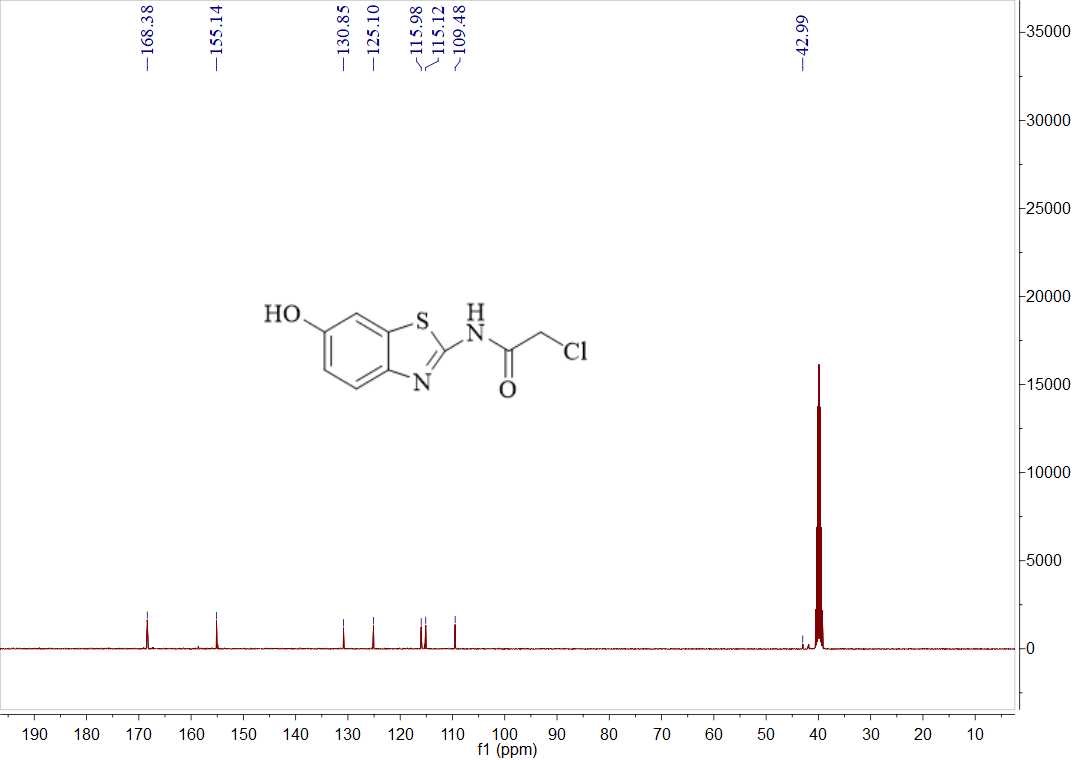
**

**Fig. S48** ^13^C NMR for compound **BTL-20**

**
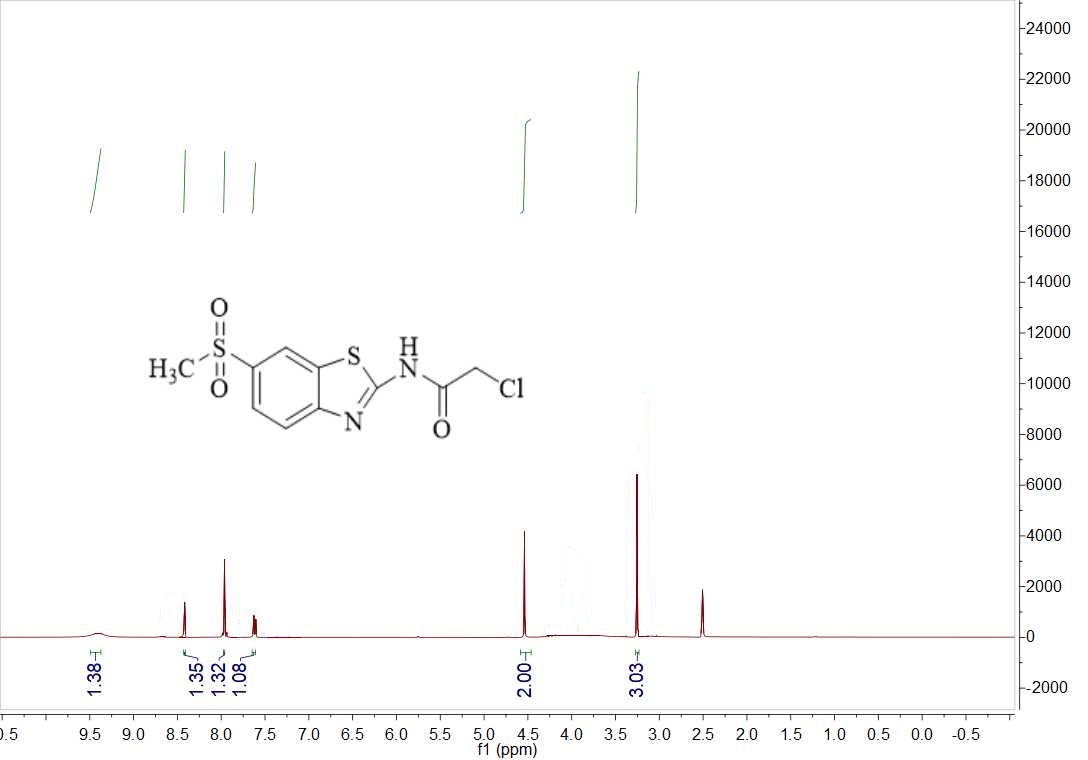
**

**Fig. S49** ^1^H NMR for compound **BTL-21**

**
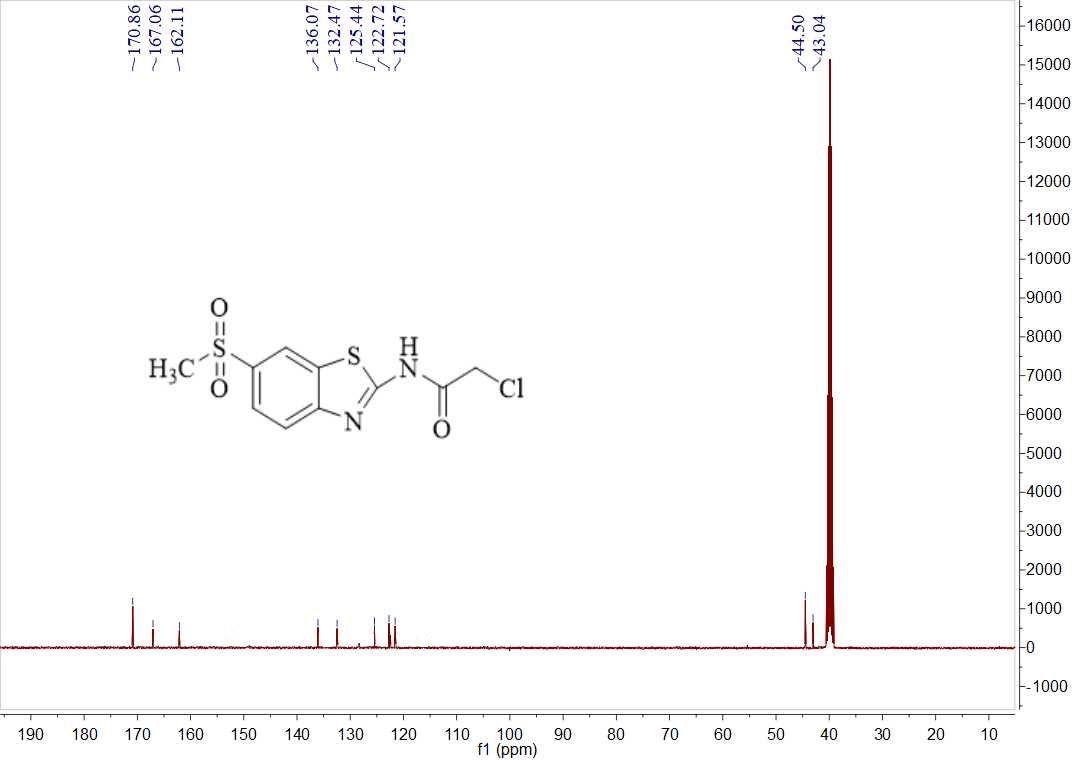
**

**Fig. S50** ^13^C NMR for compound **BTL-21**

**
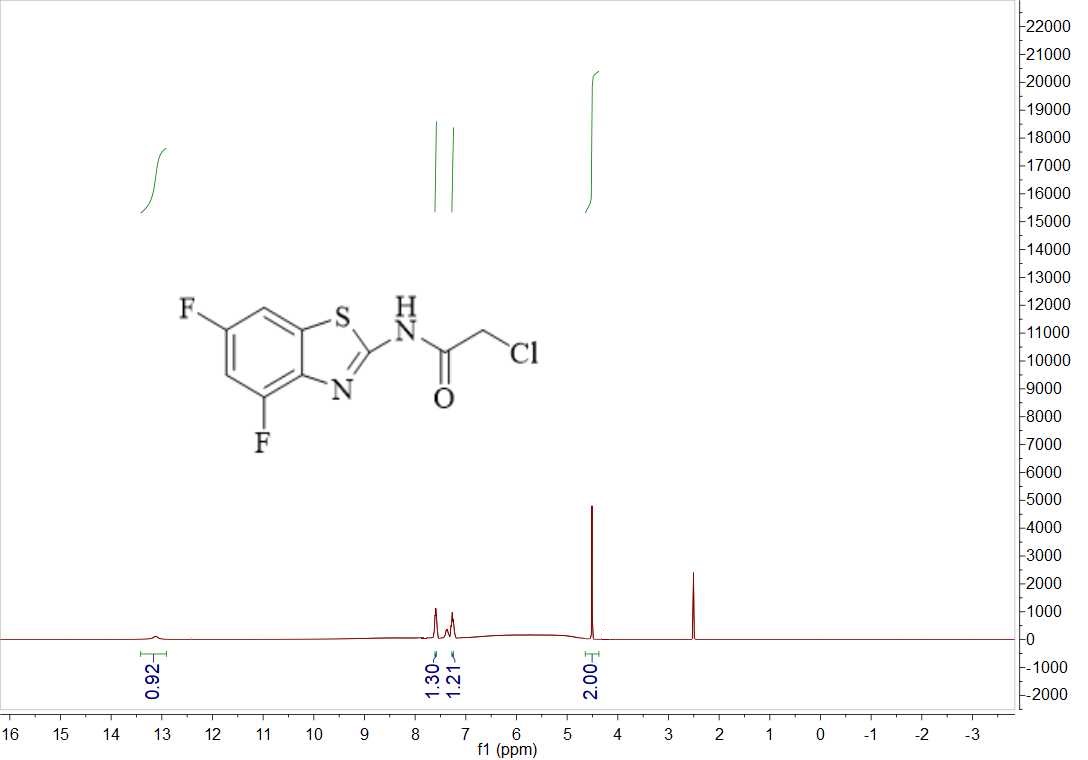
**

**Fig. S51** ^1^H NMR for compound **BTL-22**

**
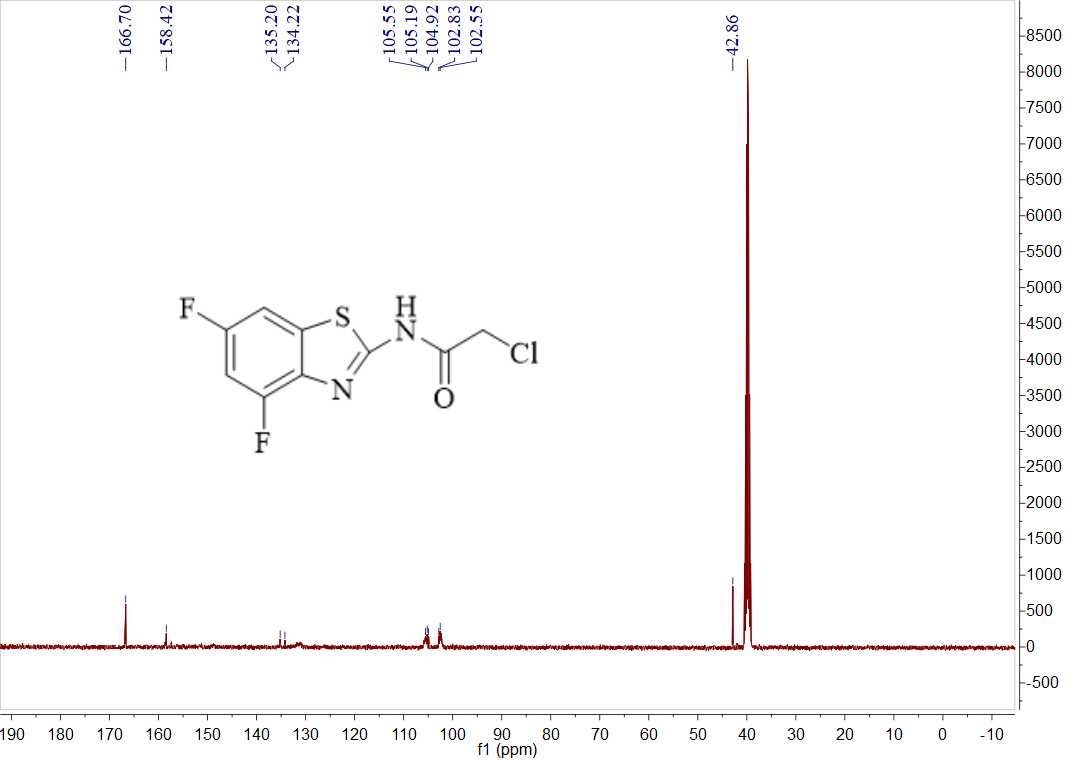
**

**Fig. S52** ^13^C NMR for compound **BTL-22**

**
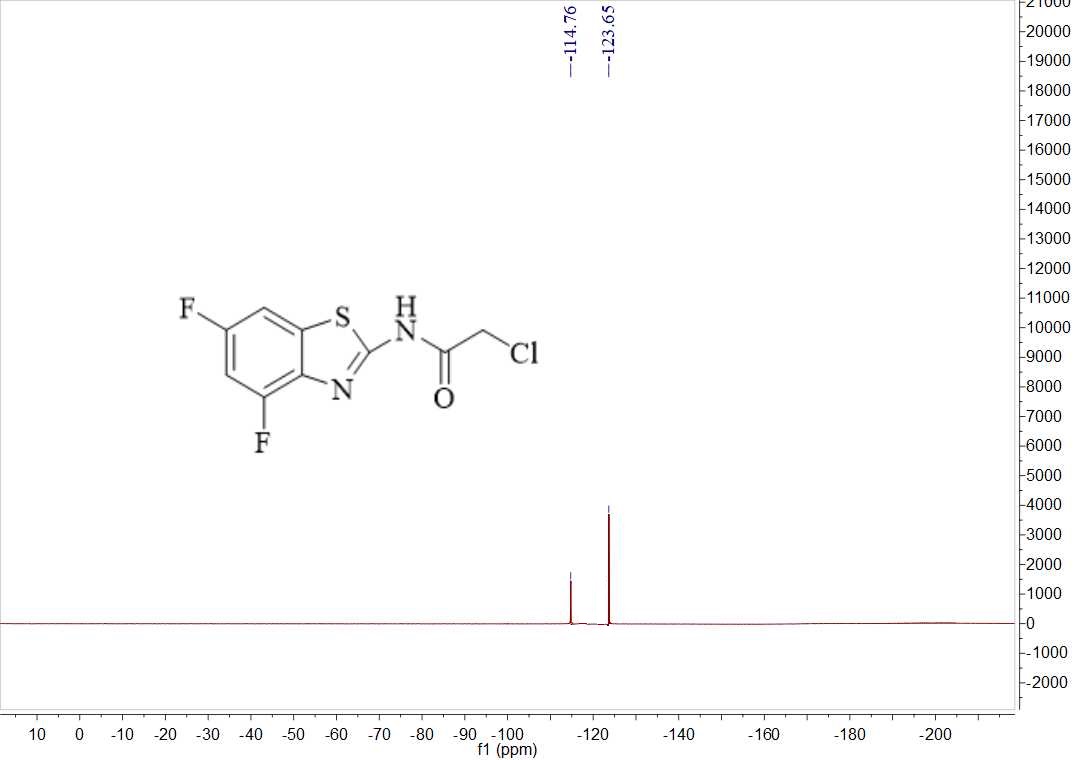
**

**Fig. S53** ^19^F NMR for compound **BTL-22**

**
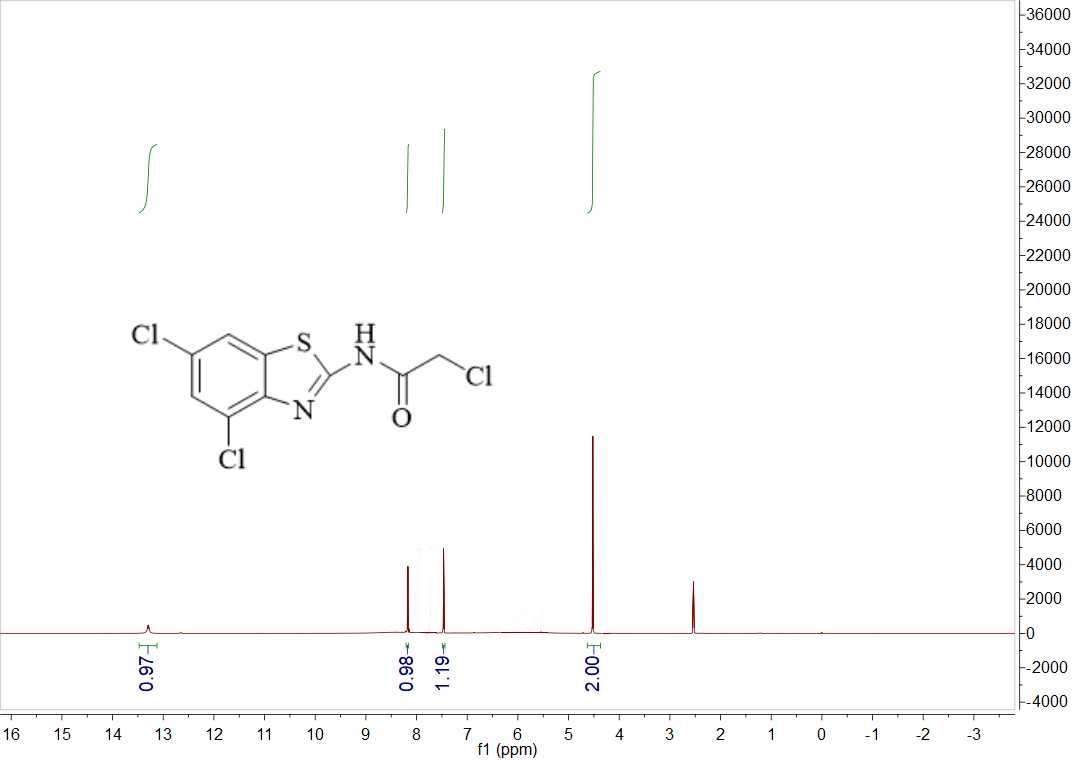
**

**Fig. S54** ^1^H NMR for compound **BTL-23**

**
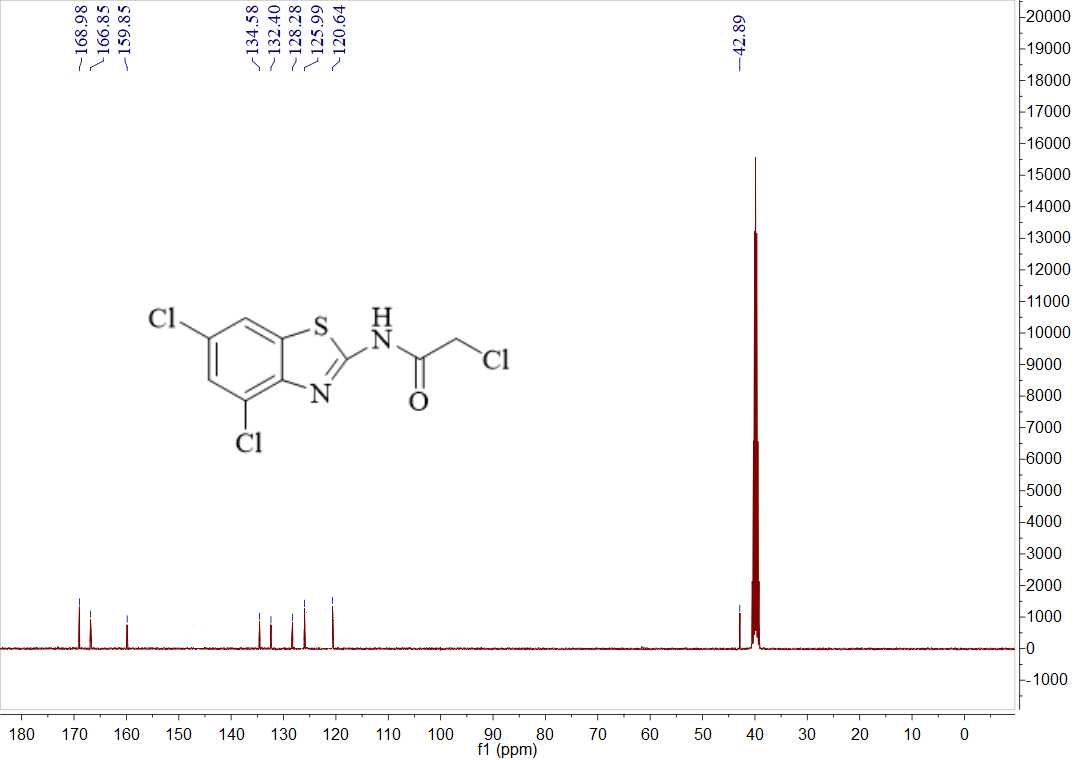
**

**Fig. S55** ^13^C NMR for compound **BTL-23**

**
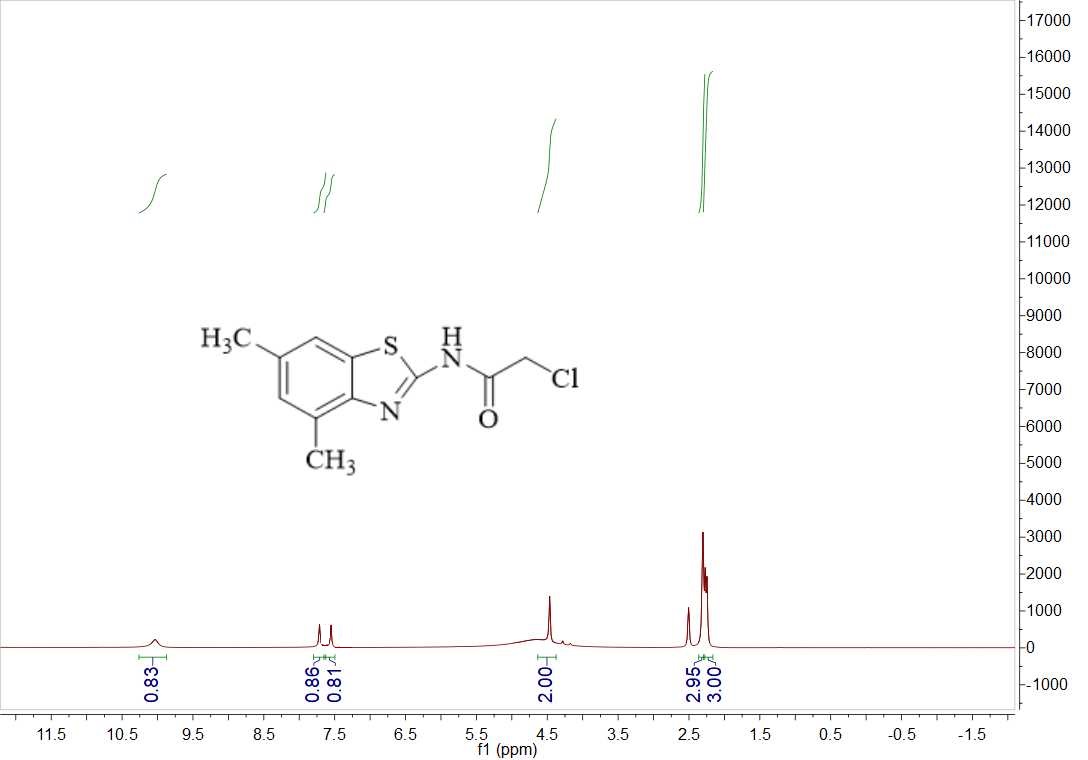
**

**Fig. S56** ^1^H NMR for compound **BTL-24**

**
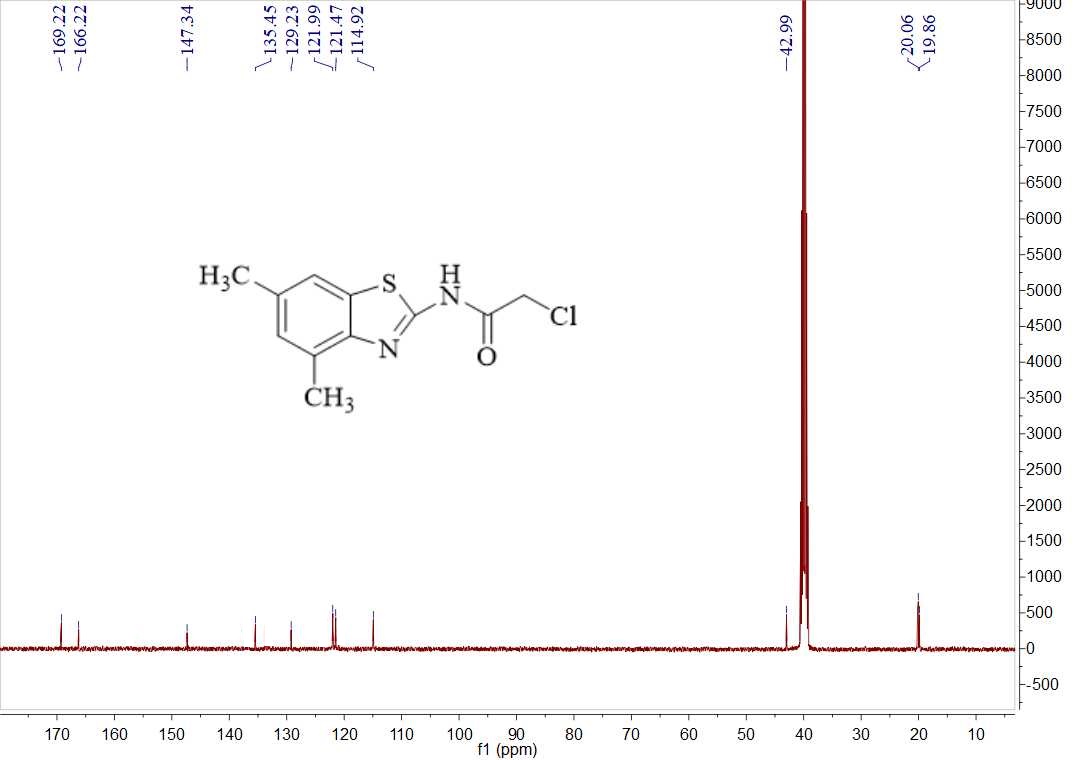
**

**Fig. S57** ^13^C NMR for compound **BTL-24**
